# Supplementary material for: Results from a cross-specialty consensus on optimal management of patients with chronic kidney disease (CKD): from screening to complications
Source: BMJ Open. 2024 Mar 7;14(3):e080891. doi: 10.1136/bmjopen-2023-080891 (PMC10921537; doi:10.1136/bmjopen-2023-080891)
Supplement: Supplementary data [file bmjopen-2023-080891supp002.pdf]

No:	Statement:	Strongly agree	Tend to agree	Tend to disagree	Strongly disagree	Overall
**Earlier identification & screening of CKD**						
**1.**	Late diagnosis is a lost opportunity for early management of CKD which can slow disease progression	77%	16%	3%	4%	**93%**
**2.**	National screening and diagnostic programmes for CKD are essential to identifying patients at earlier stages of CKD	69%	27%	3%	2%	**96%**
**3.**	A simple and practical definition of 'high-risk' should be established to support cost-effective screening	65%	32%	2%	1%	**97%**
**4.**	Early screening for CKD in high-risk groups is cost-effective for the health system (where resources are in place to support intervention)	71%	27%	1%	1%	**97%**
**5.**	Primary care physicians and specialists (cardiologists, endocrinologists, nephrologists, diabetologists, etc.) should routinely screen for CKD in patients with risk factors	76%	21%	1%	1%	**97%**
**6.**	Patients with risk factors (e.g., elderly, diabetes, hypertension, dyslipidaemia, obesity, family history of CKD) should be screened at least annually for CKD	81%	16%	2%	1%	**97%**
**7.**	Patients with CKD should be screened for heart failure (including those with preserved ejection fraction (HFpEF))	46%	46%	6%	2%	**92%**
**8.**	GFR estimated by the CKD-EPI Creatinine Equation (2021) and albuminuria (using albumin-to-creatinine ratio) should be the screening method of choice for CKD	55%	41%	4%	1%	**95%**
**9.**	Confirmatory testing of abnormalities of kidney function or structure at 3 months is required to establish a CKD diagnosis	48%	44%	7%	1%	**92%**
**Risk factors for CKD in cardio-renal-metabolic patients**						
**10.**	A holistic view of cardio-renal-metabolic disease states is critical to provide integrated patient-centred care to individuals with CKD	74%	25%	0%	1%	**99%**
**11.**	CKD progressively increases the risk of CVD, including heart failure and overall mortality	75%	24%	0%	1%	**99%**
**12.**	Frequency of hyperkalaemia increases as CKD progresses	62%	34%	3%	1%	**97%**
**13.**	AKI is a risk factor for CKD and chronic heart failure	57%	37%	5%	1%	**93%**
**14.**	Infection, including COVID-19, can cause AKI and is therefore a risk factor for CKD	42%	43%	14%	1%	**85%**
**15.**	Nephrotoxic medications should be modified, and risk/benefit profiles should be assessed in patients with, or at risk of CKD	72%	26%	1%	1%	**98%**
**Holistic management of CKD in cardio-renal-metabolic patients**						
**16.**	Lifestyle factors such as diet, smoking, exercise must be optimised for CKD patients	83%	17%	0%	0%	**100%**
**17.**	Disease modifying therapies for CKD (e.g., SGLT2i, RAASi, MRA) should be used where indicated to manage progression of CKD	69%	29%	1%	0%	**99%**
**18.**	SGLT2i and RAASi have a complementary cardio-renal protective action	61%	37%	1%	0%	**98%**
**19.**	Early use of SGLT2i could prevent the development and progression of CKD patients with type 2 diabetes and heart failure	62%	34%	3%	0%	**96%**
**20.**	Early use of SGLT2i can slow progression of CKD in patients without diabetes	45%	45%	9%	1%	**91%**
**21.**	A small decrease of eGFR (~10%) may be expected on initiation of CKD disease-modifying therapies	39%	54%	7%	0%	**93%**
**22.**	De-escalation or discontinuation of RAASi therapy is associated with worse cardiovascular and renal outcomes in the CKD patient	33%	57%	9%	1%	**90%**
**23.**	Disease modifying therapies for CKD should only be stopped as a last resort	33%	50%	15%	3%	**82%**
**24.**	Novel K binders (i.e., patiromer and sodium zirconium cyclosilicate) are an option to manage hyperkalaemia and prevent de-escalation or downtitration of RAASi	27%	65%	8%	0%	**91%**
**25.**	Action to manage hyperkalaemia in the CKD patient should be taken when serum potassium level reaches 5.0mmol/L	22%	54%	20%	5%	**75%**
**26.**	Action to manage hyperkalaemia in the CKD patient should be taken when serum potassium level reaches 5.5 mmol/L	53%	40%	5%	1%	**93%**

**27.**	Treatment targets such as blood pressure, blood glucose, lipid levels should be maintained for both kidney and cardiac outcomes in CKD patients	81%	17%	1%	0%	**98%**
**Guidelines**						
**28.**	Implementation of evidence-based CKD guidelines is suboptimal and should be improved	29%	48%	20%	2%	**77%**
**29.**	Cardiology, nephrology, and endocrinology guidelines for CKD should be aligned	62%	36%	2%	0%	**98%**
**30.**	Primary care physicians have an important role in implementing guidelines	66%	30%	4%	1%	**95%**
**31.**	Guidelines should be practical with an executive summary / checklist to assist implementation by non-specialist HCPs	65%	31%	3%	0%	**97%**
**32.**	Guidelines should include clear criteria for when and how to refer to other specialists/MDT	69%	30%	2%	0%	**98%**
**33.**	Guidelines should reflect differences in population phenotypes and be locally adapted where needed	58%	35%	6%	0%	**94%**
**34.**	Patient perspective is crucial in developing guidelines	42%	46%	11%	1%	**88%**
**35.**	Investment is needed to shift the approach to primary prevention rather than end stage kidney disease management	69%	26%	3%	1%	**96%**
**Cross-specialty alignment (Cardiology, Nephrology, Endocrinology, Primary Care & Policy Makers)**						
**36.**	Patients with CKD should be managed by a multi-disciplinary team (MDT) where possible with an agreed management plan	67%	31%	1%	0%	**99%**
**37.**	The CKD multi-disciplinary team (MDT) should include the primary care physician to improve early intervention and decision making	65%	32%	3%	0%	**97%**
**38.**	The care pathway for CKD should be designed to minimise the impact of organisational barriers	58%	39%	2%	0%	**98%**
**39.**	Clinicians, professional associations, academic institutions, and patient representative organisations need to engage with policy makers to ensure appropriate plans and funding are in place to deliver optimal CKD care	67%	31%	2%	0%	**98%**
**Education of clinicians and patients**						
**40.**	Up-to-date healthcare professional (HCP) education on the management of patients with CKD and associated guidelines is needed	72%	27%	1%	0%	**99%**
**41.**	Structured education of primary care physicians on screening and early detection improves patient outcomes	76%	24%	0%	0%	**100%**
**42.**	Therapeutic patient education is key for them to understand the consequences of CKD and how to manage it through lifestyle modification and appropriate use of therapies	73%	26%	1%	0%	**99%**
