# Supplementary figures and images for: Results from a cross-specialty consensus on optimal management of patients with chronic kidney disease (CKD): from screening to complications
Source: BMJ Open. 2024 Mar 7;14(3):e080891. doi: 10.1136/bmjopen-2023-080891 (PMC10921537; doi:10.1136/bmjopen-2023-080891)

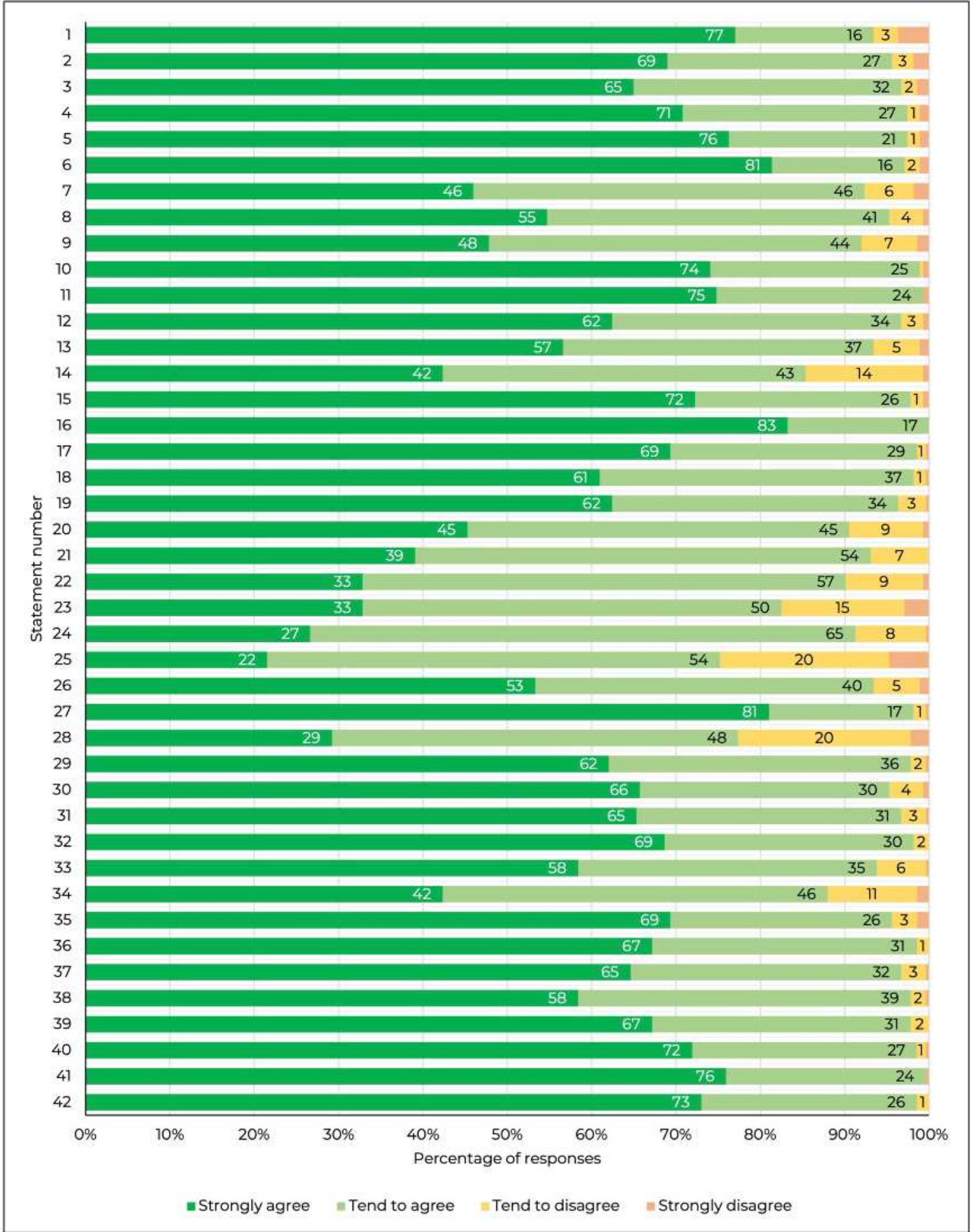

Supplement: Supplementary data [file bmjopen-2023-080891supp001.pdf]
