# Supplementary material for: Results from a cross-specialty consensus on optimal management of patients with chronic kidney disease (CKD): from screening to complications
Source: BMJ Open. 2024 Mar 7;14(3):e080891. doi: 10.1136/bmjopen-2023-080891 (PMC10921537; doi:10.1136/bmjopen-2023-080891)
Supplement: Author's manuscript [file bmjopen-2023-080891.draft_revisions.pdf]

BMJ Open is committed to open peer review. As part of this commitment we make the peer review history of every article we publish publicly available.

When an article is published we post the peer reviewers' comments and the authors' responses online. We also post the versions of the paper that were used during peer review. These are the versions that the peer review comments apply to.

The versions of the paper that follow are the versions that were submitted during the peer review process. They are not the versions of record or the final published versions. They should not be cited or distributed as the published version of this manuscript.

BMJ Open is an open access journal and the full, final, typeset and author-corrected version of record of the manuscript is available on our site with no access controls, subscription charges or pay-per-view fees (http://bmjopen.bmj.com).

If you have any questions on BMJ Open's open peer review process please email info.bmjopen@bmj.com

BMJ Open

Results from a cross-specialty consensus on optimal management of the chronic kidney disease (CKD) patient: from screening to complications

Journal:	*BMJ Open*
Manuscript ID	bmjopen-2023-080891
Article Type:	Original research
Date Submitted by the Author:	13-Oct-2023
Complete List of Authors:	Arici, Mustafa ; Hacettepe Universitesi, ; Hacettepe University Faculty of Medicine<br>Assaad – Khalil, Samir; Alexandria University Faculty of Medicine, Unit of Diabetes, Lipidology & Metabolism<br>Bertoluci, Marcello; Universidade Federal do Rio Grande do Sul Instituto de Biociencias, Endocrinology Division, Hospital de Clínicas de Porto Alegre<br>Choo, Jason; Singapore General Hospital, Department of Renal Medicine<br>Lee, Yau-Jiunn; Lee's Clinic<br>Madero, Magdalena; National Heart Institute of Mexico<br>Rosa Diez, Guillermo ; Hospital Italiano de Buenos Aires, Servicio de Nefrología<br>Sánchez Polo, Vicente ; Instituto Guatemalteco de Seguridad Social, Servicio de Nefrología y Trasplante Renal<br>Chung, Sungjin; The Catholic University of Korea College of Medicine, Division of Nephrology<br>Thanachayanont, Teerawat; Bhumirajanagarindra Kidney Institute, Nephrology<br>Pollock, Carol; University of Sydney
Keywords:	Nephrology < INTERNAL MEDICINE, Heart failure < CARDIOLOGY, Hypertension < CARDIOLOGY, Chronic renal failure < NEPHROLOGY, Primary Care < Primary Health Care, Surveys and Questionnaires

SCHOLARONE™
Manuscripts

I, the Submitting Author has the right to grant and does grant on behalf of all authors of the Work (as defined in the below author licence), an exclusive licence and/or a non-exclusive licence for contributions from authors who are: i) UK Crown employees; ii) where BMJ has agreed a CC-BY licence shall apply, and/or iii) in accordance with the terms applicable for US Federal Government officers or employees acting as part of their official duties; on a worldwide, perpetual, irrevocable, royalty-free basis to BMJ Publishing Group Ltd ("BMJ") its licensees and where the relevant Journal is co-owned by BMJ to the co-owners of the Journal, to publish the Work in this journal and any other BMJ products and to exploit all rights, as set out in our licence.

The Submitting Author accepts and understands that any supply made under these terms is made by BMJ to the Submitting Author unless you are acting as an employee on behalf of your employer or a postgraduate student of an affiliated institution which is paying any applicable article publishing charge ("APC") for Open Access articles. Where the Submitting Author wishes to make the Work available on an Open Access basis (and intends to pay the relevant APC), the terms of reuse of such Open Access shall be governed by a Creative Commons licence – details of these licences and which Creative Commons licence will apply to this Work are set out in our licence referred to above.

Other than as permitted in any relevant BMJ Author's Self Archiving Policies, I confirm this Work has not been accepted for publication elsewhere, is not being considered for publication elsewhere and does not duplicate material already published. I confirm all authors consent to publication of this Work and authorise the granting of this licence.

For peer review only

Results from a cross-specialty consensus on optimal management of the chronic kidney disease (CKD) patient: from screening to complications

Arici M M.D.[1], Assaad-Khalil S M.D.[2], Bertoluci MC M.D.[3], Choo JCJ M.B.B.S.[4], Lee Y-J M.D.[5], Madero M M.D.[6], Rosa-Diez G M.D.[7], Sánchez-Polo V M.D.[8], Chung S M.D.[9], Thanachayanont T M.D.[10], Pollock C M.B.B.S.[11]

1. Division of Nephrology, Department of Internal Medicine, Faculty of Medicine, Hacettepe University, Ankara, Turkey
2. Unit of Diabetes, Lipidology & Metabolism; Department of Internal Medicine/ Alexandria Faculty of Medicine, Alexandria University, Alexandria, Egypt
3. Endocrinology Division, Hospital de Clínicas de Porto Alegre, Universidade Federal do Rio Grande do Sul, Porto Alegre, Brazil
4. Department of Renal Medicine, Singapore General Hospital, Singapore
5. Lee's Endocrinology Clinic, Pingtung 90000, Taiwan
6. Division of Nephrology, Department of Medicine, National Heart Institute, Mexico City, Mexico
7. Servicio de Nefrología, Hospital Italiano de Buenos Aires, Buenos Aires, Argentina
8. Nephrology Department, Guatemalan Social Security Institute (IGSS)
9. Department of Internal Medicine, College of Medicine, The Catholic University of Korea, Seoul, Republic of Korea; Clinical Practice Guideline Committee, The Korean Society of Nephrology
10. Bhumirajanagarindra Kidney Institute, Bangkok Thailand
11. Kolling Institute of Medical Research, Sydney Medical School, University of Sydney, Royal North Shore Hospital, St Leonards, NSW, Australia

Correspondence to:

Professor M Arici

Division of Nephrology, Department of Internal Medicine, Faculty of Medicine,
Hacettepe University, Istanbul, Turkey
Division of Nephrology, Department of Internal Medicine,

Faculty of Medicine, Hacettepe University,

Ankara, Turkey

marici@hacettepe.edu.tr

Main text = 2,964 words

For peer review only

KEYWORDS

- Chronic Kidney Disease
- Heart Failure
- Hypertension
- Hyperkalaemia
- Primary Care
- Multidisciplinary Care Team

ABSTRACT (297 WORDS)

Background: Chronic kidney disease (CKD) affects around 10% of the global population and has been estimated to affect around 50% of individuals with type 2 diabetes and 50% of those with heart failure. The guideline recommended approach is to manage with disease-modifying therapies, but real-world data suggests that prescribing rates do not reflect this in practice.

Objective: To develop a cross-specialty consensus on optimal management of the CKD patient using a modified Delphi method.

Design: An international steering group of experts specialising in internal medicine, endocrinology/diabetology, nephrology and primary care medicine developed 42 statements on aspects of CKD management including identification and screening, risk factors, holistic management, guidelines, cross-specialty alignment, and education. Consensus was determined by agreement using an online survey.

Participants: The survey was distributed to cardiologists, nephrologists, endocrinologists, and primary care physicians across 11 countries.

Main outcomes and measures: The threshold for consensus agreement was established *a priori* by the steering group at 75%. Stopping criteria were defined as a target of 25 responses from each country (N=275), and a four-week survey period.

Results: 274 responses were received in December 2022, 25 responses from Argentina, Australia, Brazil, Guatemala, Mexico, Singapore, South Korea, Taiwan, Thailand, Turkey, and 24 responses from Egypt. 53 responses were received from cardiologists, 52 from nephrologists, 55 from endocrinologists, and 114 from primary care physicians. 37 statements attained very high agreement (≥90%) and 5 attained high agreement (≥75% and

For peer review only

<90%). Strong alignment between roles was seen across the statements, and different levels of experience (2-5 years or 5+ years), some variation was observed between countries.

Conclusions: There is a high degree of consensus regarding aspects of CKD management among healthcare professionals. Based on these strong levels of agreement, the steering group derived twelve key recommendations focused on diagnosis and management of CKD.

STRENGTHS AND LIMITATIONS OF THIS STUDY

- 274 responses received with good representation from experienced physicians across 11 different countries is a strong basis for consensus
- Good representation from different roles providing insight on the opinions held by experienced practitioners across the spectrum of CKD care
- Very high levels of agreement achieved which may suggest that the statements were constructed as to achieve agreement (confirmation bias), although this was not the intention
- Differences in health service provision (including availability of therapies) could have limited agreement in some countries

FUNDING

The study was initiated and funded by AstraZeneca. All authors received funding from AstraZeneca while undertaking this study. AstraZeneca commissioned Triducive Partners Limited to facilitate the project and analyse the responses to the consensus statements in line with the Delphi methodology.

CONFLICTS OF INTEREST

M Arici has received payment honoraria for lectures, presentations, speaker bureaus, publication writing or educational events from: Amgen, AstraZeneca, Bayer, Boehringer Ingelheim, Menarini, MSD, Novo Nordisk, Sandoz, and Sanofi. M Arici has also participated in a Data Safety Monitoring Board or Advisory Boards for*:* AstraZeneca, Bayer, Boehringer Ingelheim, and Novo Nordisk. MC Bertoluci has worked as speaker for: Aché, AstraZeneca, Bayer, Boehringer-Ingelheim, Novo-Nordisk, and Servier. JCJ Choo has consulted for, or been on advisory boards for: AstraZeneca, ATC, Bayer, GSK, Nitto Denko, and Pfizer. JCJ Choo has been a speaker for: Abbott, AstraZeneca, Bayer, and Boehringer Ingelheim. JCJ Choo has been on Steering Committees or Data Safety Monitoring Boards for: AstraZeneca, Boehringer Ingelheim, and Novartis, and has received scientific grant funding from: AstraZeneca, ATC, and Nitto Denko. Y-J Lee has been a speaker and Advisory Board member for: AstraZeneca Taiwan. M Madero was the International Society for Nephrology Deputy Chair for Latin America and

For peer review only

Caribbean (2019-2023) and is currently the on the council for the International Society for Peritoneal Disease (2022-2024). M Madero has been a consultant for: AstraZeneca, Bayer, and Boehringer Ingelheim, and provided expert testimony for: AstraZeneca, Bayer, and Boehringer Ingelheim. M Madero has received grants from or has pending grants with: AstraZeneca*, Bayer*, Boehringer, Renal Research Institute*, and Tricida*. M Madero has received travel grants from: AstraZeneca and Medcom. GJ Rosa Diez has been a speaker or Advisory Board member for: Astra Zeneca, Boehringer Ingelheim, Bayer, Fresenius, and Medtronic. S Chung has received honoraria as a speaker or advisory board member from: AstraZeneca, Boehringer Ingelheim, Daewon, Daewoong, Eli Lilly, Korea Pharma, Myoung Poom Medical, Otsuka, Sanofi-Aventis, and Yuhan. C Pollock has been a speaker or participated in advisory boards for Astra Zeneca, Boehringer Ingelheim, Eli Lilly, GlaxoSmithKline, Otsuka, Vifor-CSL. S Assad-Khalil, V Sánchez-Polo, and T Thanachayanont declare no conflict of interest in regard to this study.

For peer review only

WORD COUNT = 2964

BACKGROUND

Chronic kidney disease (CKD) is defined as abnormalities of kidney structure or function, which are present for >3 months[1]. This is indicated by a glomerular filtration rate (GFR) below 60 mL/min/1.73 m², or the presence of one or more markers of kidney damage. CKD is classified based on cause, glomerular filtration rate category, and albuminuria category[1]. Diabetes is the leading cause of CKD, followed by hypertension, suggesting a close relationship between the cardio-renal-metabolic systems[2]. The interplay between CKD, cardiovascular disease (CVD), and metabolic diseases (such as type 2 diabetes mellitus (T2DM)) is significant and has been termed 'cardio-renal-metabolic' (CRM) disease[3]. It has been suggested that CKD affects around 50% of people with T2DM[4], around 50% of heart failure (HF) patients[5], and up to 38% of those with hypertension[6], suggesting the need for a holistic approach to management of CKD patients.

Between 1990 and 2017, the prevalence of CKD was estimated to rise to 9.1% of the global population, with associated mortality increasing by 41.5%[7]. This is closely associated with an increase in populations with risk factors such as diabetes, hypertension, and prediabetes[2]. The individual burden of CKD falls disproportionately upon those with lower socioeconomic status. This population is associated with a higher prevalence of CKD and variable access to treatment in some health economies, and ultimately poorer outcomes. In low- and middle-income countries, an estimated 188 million people experience catastrophic health expenditure annually due to kidney diseases, the greatest of any disease group[8].

Early diagnosis and referral of CKD are key to enabling treatment to delay or halt progression to kidney failure, providing education to the patient and reducing morbidity and mortality. The absence of symptoms in early stages of CKD requires that clinicians maintain an adequate suspicion in all patients and especially those with risk-factors[9]. Accurate screening, diagnosis, and risk stratification need to be in place to support the aim of 'earliest diagnosis'.

A diagnosis of CKD is determined by laboratory confirmation of proteinuria or haematuria, and/or a reduction in the glomerular filtration rate (GFR), for more than 3 months' duration[10]. Two key markers of CKD include albuminuria (defined as a urinary albumin-to-creatine ratio [uACR] of $\geq$30 mg/g) and reduction in estimated GFR (eGFR), as calculated by the CKD-EPI based on creatinine alone ($eGFR_{cr}$) or based on creatinine and cystatin C ($eGFR_{cr\text{-}cys}$)[10,11]. In practice, these methods may not be utilised effectively, an analysis of over 123,000 patient records

For peer review only - http://bmjopen.bmj.com/site/about/guidelines.xhtml

found low frequency of uACR testing in patients with CKD, despite strong epidemiological evidence linking increased albuminuria with disease progression, kidney failure, cardiovascular events, and premature mortality[12].

Besides lifestyle modifications, disease-modifying therapies (DMTs) for CKD including renin-angiotensin-aldosterone system inhibitors (RAASi), and sodium-glucose co-transporter-2 inhibitors (SGLT2i), as recommended by KDIGO for use in CKD patients with hypertension or diabetes, and non-steroidal mineralocorticoid antagonists (ns-MRAs) as add-on therapy in patients with T2DM and residual albuminuria[1,13]. Despite the availability of these therapies, analysis of 2 large US healthcare systems showed that even though nearly two-thirds of the adults with CKD had diabetes, hypertension, or prediabetes, rates of prescribing RAASi were low. Not only was DMT use below guideline directed levels, but potentially nephrotoxic agents (such as non-steroidal anti-inflammatory drugs and proton pump inhibitors) were used more commonly than RAASi[2].

There is clearly some disconnect between guideline recommendations and real-world practice. Guidelines for the management of CKD tend to be produced by disease specialists but may not be clear or made known to primary care practitioners (PCPs). Furthermore, applicability or ease of implementation may be questionable in low to middle income economies[14,15].

The objective of this project is to use a modified Delphi technique to examine the opinions of healthcare professionals towards aspects of CKD management across 11 countries, report these findings, and develop practical recommendations for diagnosis and management of CKD.

METHODS

A steering group of experts from 11 different countries convened in September 2022 to discuss current challenges in CKD management through a nominal group technique. The experts were defined as specialists in nephrology, endocrinology/diabetology, internal medicine, and primary care who had achieved an appropriate level of seniority within their field (for example professors or clinical directors), or had published papers related to the management of CKF/HF/Diabetes, or had been involved in guideline development. The steering group were selected from outside of Europe and North America to avoid replication of previous published work.[16]

Using a modified Delphi methodology guided by an independent facilitator (Triducive Partners Limited), the process outlined in Figure 1 was used.

For peer review only - http://bmjopen.bmj.com/site/about/guidelines.xhtml

Figure 1: Study flow diagram

<<Figure 1>>

The following themes were agreed during Steering Group Meeting 1:

1. Earlier identification & screening of CKD
2. Risk factors for CKD in cardio-renal-metabolic patients
3. Holistic management of CKD in cardio-renal-metabolic patients
4. Guidelines
5. Cross-specialty alignment (Cardiology, Nephrology/Diabetology, Endocrinology, Primary Care & Policy Makers)
6. Education of clinicians and patients

These domains were discussed in detail and statements were suggested by the Steering Group working collaboratively, these statements were then collated, and the steering group independently rated the statements as either (accept, remove, or reword) with suggested changes as needed. The final statement set was then This constituted the initial round of consensus. The resulting agreed 42 *Likert* scale statements were developed for testing, the identity of respondents was not known to the steering group or the independent facilitator.

For each statement, respondents were offered a 4-point scale ('strongly disagree', 'tend to disagree', 'tend to agree', and 'strongly agree') to allow them to indicate their level of agreement with each statement. The survey captured some demographic data for further analyses including country, specialty, length of time in role, and average number of CKD patients managed over 3 months. Stopping criteria were defined as a target of 25 responses from each country (N=275), and a four-week survey period. The threshold for consensus agreement was defined *a priori* at 75%, a widely accepted threshold[17]. This was further defined as 'high' at ≥75% and 'very high' at ≥90%. Completed surveys were collated and analysed to produce an arithmetic agreement score for each statement, in line with Delphi methodology[18].

As this study only sought the anonymous opinions of physicians and no patient-specific data were captured, ethical approval was not sought. Respondents provided consent to have their responses included in this study through their inclusion to the panel held by Sermo. Patients and public were not involved in this research as it is primarily concerned with healthcare policy and practice.

RESULTS

Completed surveys from 274 responders were analysed to define the total level of agreement with each of the 42 statements. Respondents were either PCPs or consultants in cardiology, nephrology and endocrinology (including diabetology) (Figure 1). Most responders had more than 10- years' experience in role (159/274) and only 13% (n=35) had less then 5 years in role. When asked to estimate the number of patients seen in a 3-month period, 177 responders (65%) stated that they saw more than 50 patients (either as inpatients or outpatients). This suggests the respondent cohort was sufficiently experienced to provide insight into aspects of CKD.

Figure 2: Respondents by role

<<Figure 2>>

Consensus was achieved for all statements, with very high agreement (≥90%) in 37 (88%) statements, and high agreement (≥75 & ≤89%) in 5 (12%) statements. Overall consensus agreement by statement is shown in Figure 3, responses according to topic are shown in Tables 1. The range of responses at each agreement level for all statements is shown in the appendix (Figure S1).

Figure 3: Overall consensus agreement levels by statement[a]

<<Figure 3>>

[a]The green horizontal line represents the 75% threshold for consensus agreement and the blue line indicates the threshold for very high consensus (90%).

Table 1. Agreement to each statement, divided by topic.

No:	Statement:	Agreement:
	**Earlier identification & screening of CKD**	
**1.**	Late diagnosis is a lost opportunity for early management of CKD which can slow disease progression	**93%**
**2.**	National screening and diagnostic programmes for CKD are essential to identifying patients at earlier stages of CKD	**96%**
**3.**	A simple and practical definition of 'high-risk' should be established to support cost-effective screening	**97%**
**4.**	Early screening for CKD in high-risk groups is cost-effective for the health system (where resources are in place to support intervention)	**97%**
**5.**	Primary care physicians and specialists (cardiologists, endocrinologists, nephrologists, diabetologists, etc.) should routinely screen for CKD in patients with risk factors	**97%**
**6.**	Patients with risk factors (e.g., elderly, diabetes, hypertension, dyslipidaemia, obesity, family history of CKD) should be screened at least annually for CKD	**97%**

For peer review o

**7.**	Patients with CKD should be screened for heart failure (including those with preserved ejection fraction (HFpEF))	**92%**
**8.**	GFR estimated by the CKD-EPI Creatinine Equation (2021) and albuminuria (using albumin-to-creatinine ratio) should be the screening method of choice for CKD	**95%**
**9.**	Confirmatory testing of abnormalities of kidney function or structure at 3 months is required to establish a CKD diagnosis	**92%**
**Risk factors for CKD in cardio-renal-metabolic patients**		
**10.**	A holistic view of cardio-renal-metabolic disease states is critical to provide integrated patient-centred care to individuals with CKD	**99%**
**11.**	CKD progressively increases the risk of CVD, including heart failure and overall mortality	**99%**
**12.**	Frequency of hyperkalemia increases as CKD progresses	**97%**
**13.**	AKI is a risk factor for CKD and chronic heart failure	**93%**
**14.**	Infection, including COVID-19, can cause AKI and is therefore a risk factor for CKD	**85%**
**15.**	Nephrotoxic medications should be modified, and risk/benefit profiles should be assessed in patients with, or at risk of CKD	**98%**
**Holistic management of CKD in cardio-renal-metabolic patients**		
**16.**	Lifestyle factors such as diet, smoking, exercise must be optimised for CKD patients	**100%**
**17.**	Disease modifying therapies for CKD (e.g., SGLT2i, RAASi, MRA) should be used where indicated to manage progression of CKD	**99%**
**18.**	SGLT2i and RAASi have a complementary cardio-renal protective action	**98%**
**19.**	Early use of SGLT2i could prevent the development and progression of CKD patients with type 2 diabetes and heart failure	**96%**
**20.**	Early use of SGLT2i can slow progression of CKD in patients without diabetes	**91%**
**21.**	A small decrease of eGFR (~10%) may be expected on initiation of CKD disease-modifying therapies	**93%**
**22.**	De-escalation or discontinuation of RAASi therapy is associated with worse cardiovascular and renal outcomes in the CKD patient	**90%**
**23.**	Disease modifying therapies for CKD should only be stopped as a last resort	**82%**
**24.**	Novel K binders (i.e., patiromer and sodium zirconium cyclosilicate) are an option to manage hyperkalaemia and prevent de-escalation or downtitration of RAASi	**91%**
**25.**	Action to manage hyperkalaemia in the CKD patient should be taken when serum potassium level reaches 5.0mmol/L	**75%**
**26.**	Action to manage hyperkalaemia in the CKD patient should be taken when serum potassium level reaches 5.5 mmol/L	**93%**
**27.**	Treatment targets such as blood pressure, blood glucose, lipid levels should be maintained for both kidney and cardiac outcomes in CKD patients	**98%**
**Guidelines**		
**28.**	Implementation of evidence-based CKD guidelines is suboptimal and should be improved	**77%**

**29.**	Cardiology, nephrology, and endocrinology guidelines for CKD should be aligned	**98%**
**30.**	Primary care physicians have an important role in implementing guidelines	**95%**
**31.**	Guidelines should be practical with an executive summary / checklist to assist implementation by non-specialist HCPs	**97%**
**32.**	Guidelines should include clear criteria for when and how to refer to other specialists/MDT	**98%**
**33.**	Guidelines should reflect differences in population phenotypes and be locally adapted where needed	**94%**
**34.**	Patient perspective is crucial in developing guidelines	**88%**
**35.**	Investment is needed to shift the approach to primary prevention rather than end stage kidney disease management	**96%**
**Cross-specialty alignment (Cardiology, Nephrology, Endocrinology, Primary Care & Policy Makers)**		
**36.**	Patients with CKD should be managed by a multi-disciplinary team (MDT) where possible with an agreed management plan	**99%**
**37.**	The CKD multi-disciplinary team (MDT) should include the primary care physician to improve early intervention and decision making	**97%**
**38.**	The care pathway for CKD should be designed to minimise the impact of organisational barriers	**98%**
**39.**	Clinicians, professional associations, academic institutions, and patient representative organisations need to engage with policy makers to ensure appropriate plans and funding are in place to deliver optimal CKD care	**98%**
**Education of clinicians and patients**		
**40.**	Up-to-date healthcare professional (HCP) education on the management of patients with CKD and associated guidelines is needed	**99%**
**41.**	Structured education of primary care physicians on screening and early detection improves patient outcomes	**100%**
**42.**	Therapeutic patient education is key for them to understand the consequences of CKD and how to manage it through lifestyle modification and appropriate use of therapies	**99%**

As none of the statements failed to reach the predetermined threshold of 75%, only one round of survey was required. The results of the survey represent current opinions of the respondents and are not intended to contradict the established evidence base.

DISCUSSION **(Note, in the discussion below, 'S' is used to denote 'statement'.)**

Earlier identification & screening of CKD (S1-9)

There was very high agreement (>90%) with all statements in this topic, and the key principles that early diagnosis of CKD is key to implementing strategies to slow disease progression. To this end, national kidney health screening

For peer review only

and diagnostic programmes should be implemented. Universal screening of the general population has been found not to be cost-effective[19], but systematic review has shown screening to be cost-effective in patients with hypertension or diabetes[20]. Indeed, a KDIGO Controversies Conference concluded that targeted groups, such as those with hypertension, diabetes, or cardiovascular disease should be screened for CKD, and that an individualised approach should be taken to screen others based on a range of factors[8].

Patients with risk factors should be screened for CKD at least annually by using eGFR and uACR.
The definition of 'high-risk' is not universal, and this may vary between populations, but there is a clear need for such a definition to be established. A minimal-resource pre-screening tool has been developed and globally validated for CKD in people with T2DM. This demonstrates that age, gender, body mass index, duration of diabetes, and blood pressure information can be used to identify those at a higher risk of a reduced GFR (and therefore increased risk of CKD)[21]. This model does not require sophisticated diagnostics and can be used to guide cost-effective screening for CKD where resources are limited.

Risk factors for CKD in cardio-renal-metabolic patients (S10-15)

All statements in this topic achieved >90% consensus apart from statement 14 (85%). This is interesting and could be due to the wording of the statement and the specific use of COVID-19 as an example. Analysis by country found that Taiwan agreement was at 68% for this statement, noticeably lower than for other countries. Although 93% of respondents agree that AKI is a risk-factor for CKD and HF, it is interesting that 7% do not agree given the evidence base[22,23].

Given the established interplay between the cardiac, renal and endocrine systems, it is heartening to see that 99% of respondents agree that a holistic approach is needed to provide personalised care for individuals with CKD. Models of care should be developed to deliver integrated multidisciplinary care to patients with cardio-renal-metabolic disease as described by the CardioMetabolic Care Alliance to use comprehensive, patient-centred, team-based approaches for aggressive secondary prevention[24]. Implementing combined clinics to deliver medical care for patients with kidney disease and diabetes or CVD may reduce outpatient healthcare costs without compromising health outcomes[25]. The use of combined multidisciplinary clinics in managing patients with diabetes, CKD, and CVD is also associated with a slower decline in GFR than usual care, and a significant reduction in the risk for all-cause mortality[26].

Holistic management of CKD in cardio-renal-metabolic patients (S16-27)

For peer review only

Very high agreement was observed for 10 of the statements in this topic, but two statements achieved lower levels of agreement (S23 and S25, 82% and 75%, respectively). Response to statement 23 is interesting, given the current debate regarding the benefits of stopping RAASi in advanced CKD. Results from a 52-week open-label UK study in patients with CKD stages 4-5 concluded ‘The STOP-ACEi trial did not find any benefit by stopping RAASi in advanced CKD’, and that ‘a knee jerk response of stopping RAASi in advanced CKD at an arbitrary GFR threshold is not the optimal approach’. Large observational studies have confirmed the cardioprotective benefits of RAASi in advanced CKD (including patients with concomitant T2DM)[27-29]. Linked to this is statement 21, a small decrease in eGFR is to be expected upon initiation of DMT for CKD. During initiation and up-titration of RAASi treatment, a decline in kidney function of up to 30% within 4 weeks can be acceptable[1,30] and it is important to avoid a knee-jerk response and reduce or stop DMT, and consultation with a Nephrologist or multidisciplinary team (MDT) is recommended.

Hyperkalaemia (HK) is potentially life-threatening; it may be acute or chronic and individuals with CKD are at an increased risk, which increases with the later stages of CKD[31]. HK treatment is often stratified by serum potassium level, with borderline levels warranting modification of dietary potassium intake, followed by pharmaceutical management for larger or more sustained increases[32]. Evidence to support recommending low potassium diets to patients with advanced CKD or ESRD is weak. Observational studies report weak associations between dietary potassium intake and potassium concentration and this approach may deprive patients of the beneficial cardiovascular effects associated with potassium-rich diets[33].

When HK occurs, RAASi dose reduction or discontinuation are the most common utilized therapeutic options, but this approach is associated with worse cardiorenal outcomes and increased mortality[34-36]. Once stopped, RAASi treatment is rarely reinitiated[37]. In cases of mild-to-moderate hyperkalaemia, DMT should be maintained where possible[1,13], and an option for achieving this is using a potassium lowering therapy such as patiromer or sodium zirconium cyclosilicate (S24, 91%).

Whilst the threshold for intervention in HK may vary by country and even individual physician, we can conclude that action may be considered when K^+ concentration reaches 5.0 mmol/L (S25, 75%) and certainly when at 5.5 mmol/L (S26, 93%) but with a degree of personalisation (in consultation with a nephrologist). Statements 25 and 26 were included to try and understand where the weight of opinion fell regarding interventions to manage hyperkalaemia, both statements achieved consensus, but the strongest agreement was for statement 26. On reflection, these

For peer review only

statements could have been worded better to understand what would constitute appropriate action in these circumstances.

Respondents very strongly agree that SGLT2i and RAASi therapies have a complementary cardio-renal protective action (S18, 98%) and that in patients with T2DM and HF, early SGLT2i use can prevent the development and progression of CKD (S19, 96%), in fact, SGLT2i may slow progression of CKD in patients without T2DM (S20, 91%). It is therefore recommended that SGLT2i and RAASi therapies are initiated as early as possible to both delay CKD progression and to therapeutically address the full cardio-renal-metabolic continuum – including T2DM and HF.

Guidelines (S28-35)

The interplay between CKD, diabetes, and CVD have been discussed, but CKD is associated with a number of comorbidities. A prospective UK cohort study of 1,741 people with CKD stage 3 found that isolated CKD (without comorbidities) was present in only 4% of patients, and that common comorbidities include 'painful condition' (30%), anaemia (24%), thyroid disorders (12%), cerebrovascular disease (12%), and respiratory conditions (10%). These comorbidities should be identified and managed as part of a multidisciplinary approach[38].

Whilst all statements in this topic achieved consensus threshold, the steering group were surprised that 23% of respondents did not agree that implementation of CKD guidelines is suboptimal. The lowest agreement levels were observed in South Korea (68%) and Guatemala (72%). This could be either to a genuine strength in implementation of guidelines in these countries which should be investigated and replicated elsewhere, or the need for continuing professional development (CPD) even amongst experienced physicians.

Due to the vital role of primary care must play in identifying and referring suspected CKD patients, clear guidelines for referral of patients are essential (S32, 98%). PCPs should be provided with clear criteria for referral and followed through with continuing education from an appropriate specialist (ideally a nephrologist). The focus on primary prevention rather than advanced CKD management can only be achieved through a whole-system approach in conjunction with PCPs and with appropriate investment in awareness, screening, and diagnosis programmes (S35, 96%).

Cross-specialty alignment (Cardiology, Nephrology, Endocrinology, Primary Care & Policy Makers) (S36-39)

For peer review only

Very high levels of agreement suggest strong support for multidisciplinary involvement in CKD management, and that PCPs should form part of the MDT. Given the prominent status of CKD as an emerging global health threat which impacts multiple organ systems and is associated with markedly increased risk of cardiovascular morbidity and mortality[38], there is a need to ensure that plans and funding are in place to deliver optimal care. Stakeholders should engage with both local and national policymakers to ensure that CKD is appropriately prioritised.

Education of clinicians and patients (S40-42)

Most responders agree on the need for up-to-date education for all healthcare professional roles involved in CKD management, but PCPs require structured education.

For peer review only

RECOMMENDATIONS

1. Early screening for CKD in high-risk groups is cost-effective for the health system (where resources are in place to support intervention)
2. GFR (estimated by the CKD-EPI Creatinine Equation) and uACR (using albumin-to creatinine ratio) should be the screening method of choice for CKD
3. The CKD multi-disciplinary team (MDT) should include the primary care physician to improve early intervention and decision making
4. Intervene early in CKD patients with proven therapies (i.e., SGLT2i, RAASi, DMTs) to delay/prevent progression to kidney failure
5. RAASi therapies should be optimised in all CKD and HF patients
6. Patients with CKD should have their hyperkalaemia managed appropriately when serum potassium level rises above >5.0 mmol/L
7. Chronic hyperkalaemia should be treated with a potassium binder (e.g., patorimer, sodium zirconium cyclosilicate) to allow for the maintenance of disease-modifying therapies
8. Guidelines should be practical with an executive summary/checklist to assist implementation by non-specialist HCPs
9. Guidelines should include clear criteria for when and how to refer to other specialists/MDT
10. Nephrologists should work together to deliver consistent and clear education regarding CKD management
11. Clinicians, professional associations, academic institutions, and patient representative organisations need to engage with policymakers to ensure appropriate plans and funding are in place to deliver optimal CKD care

12. National kidney health programmes should be implemented to drive improvements to screening and diagnosis]

CONCLUSION

The Steering Group was able to form a strong set of recommendations based on the high levels of agreement achieved for most statements. Implementation of these recommendations has the potential to improve detection of CKD at an earlier stage in patients with risk factors. Earlier diagnosis provides the opportunity for early intervention with DMTs that can slow or halt the progression of CKD, in addition to reducing associated morbidity and mortality.

DECLARATIONS

Ethics approval and consent to participate:

Not applicable

Author contributions: All views expressed by the authors are those of the individual and do not represent those of their organisation/place of work. All authors contributed equally to the development, editing and review of the final document.

Acknowledgements: The authors would like to thank Ian Walker and Dal Singh of Triducive Partners Limited for their assistance in analysing the results, writing the manuscript, and reviewing the final draft.

For peer review only

REFERENCES

1. Kidney Disease: Improving Global Outcomes (KDIGO) 2022 Clinical Practice Guideline for Diabetes Management in Chronic Kidney Disease Kidney International (2022) 102 (Suppl 5S), S1–S127 S1.
2. Tuttle KR, Alicic RZ, Duru OK, et al. Clinical Characteristics of and Risk Factors for Chronic Kidney Disease Among Adults and Children: An Analysis of the CURE-CKD Registry. JAMA Netw Open. 2019 Dec 2;2(12):e1918169. doi: 10.1001/jamanetworkopen.2019.18169. PMID: 31860111; PMCID: PMC6991307.
3. Kadowaki T, Maegawa H, Watada H, et al. Interconnection between cardiovascular, renal and metabolic disorders: A narrative review with a focus on Japan. Diabetes Obes Metab. 2022 Dec;24(12):2283-2296. doi: 10.1111/dom.14829. Epub 2022 Aug 25. PMID: 35929483; PMCID: PMC9804928.
4. Thomas MC, Cooper ME, Zimmet P. Changing epidemiology of type 2 diabetes mellitus and associated chronic kidney disease. Nat Rev Nephrol. 2016 Feb;12(2):73-81. doi: 10.1038/nrneph.2015.173. Epub 2015 Nov 10. PMID: 26553517.
5. van de Wouw J, Broekhuizen M, Sorop O, et al. Chronic Kidney Disease as a Risk Factor for Heart Failure With Preserved Ejection Fraction: A Focus on Microcirculatory Factors and Therapeutic Targets. Front Physiol. 2019 Sep 4;10:1108. doi: 10.3389/fphys.2019.01108. PMID: 31551803; PMCID: PMC6737277.
6. da Silva LS, Cotta RMM, Moreira TR, et al. Hidden prevalence of chronic kidney disease in hypertensive patients: the strategic role of primary health care. Public Health. 2016 Nov;140:250-257. doi: 10.1016/j.puhe.2016.02.029. Epub 2016 Mar 29. PMID: 27036982.
7. Bikbov B, Purcell C, Levey A, et al. (2020). Global, regional, and national burden of chronic kidney disease, 1990–2017: a systematic analysis for the Global Burden of Disease Study 2017. The Lancet. 395. 709-733. 10.1016/S0140-6736(20)30045-3.
8. Shlipak MG, Tummalapalli SL, Boulware LE, et al. The case for early identification and intervention of chronic kidney disease: conclusions from a Kidney Disease: Improving Global Outcomes (KDIGO) Controversies Conference. Kidney Int. 2021 Jan;99(1):34-47. doi: 10.1016/j.kint.2020.10.012. Epub 2020 Oct 27. PMID: 33127436.
9. Bastos MG, Kirsztajn GM. Chronic kidney disease: importance of early diagnosis, immediate referral and structured interdisciplinary approach to improve outcomes in patients not yet on dialysis. J Bras Nefrol. 2011 Mar;33(1):93-108. English, Portuguese. PMID: 21541469.
10. Kidney Disease: Improving Global Outcomes (KDIGO) CKD Work Group. KDIGO 2012 Clinical Practice Guideline for the Evaluation and Management of Chronic Kidney Disease. Kidney inter., Suppl. 2013; 3: 1–150.
11. Inker LA, Eneanya ND, Coresh J, et al. New Creatinine- and Cystatin C-Based Equations to Estimate GFR without Race. N Engl J Med. 2021 Nov 4;385(19):1737-1749. doi: 10.1056/NEJMoa2102953. Epub 2021 Sep 23. PMID: 34554658; PMCID: PMC8822996.
12. James G, Garcia Sanchez JJ, Carrero JJ, et al. Low Adherence to Kidney Disease: Improving Global Outcomes 2012 CKD Clinical Practice Guidelines Despite Clear Evidence of Utility. Kidney Int Rep. 2022 Jun 8;7(9):2059-2070. doi: 10.1016/j.ekir.2022.05.033. PMID: 36090504; PMCID: PMC9458998.
13. Kidney Disease: Improving Global Outcomes (KDIGO) Blood Pressure Work Group. Kidney Int. 2021 Mar;99(3S):S1-S87. Doi: 10.1016/j.kint.2020.11.003. PMID: 33637192.
14. Sperati CJ, Soman S, Agrawal V, et al; National Kidney Foundation Education Committee. Primary care physicians' perceptions of barriers and facilitators to management of chronic kidney disease: A mixed methods study. PLoS One. 2019 Aug 22;14(8):e0221325. doi: 10.1371/journal.pone.0221325. PMID: 31437198; PMCID: PMC6705804.
15. Lamprea-Montealegre JA, Joshi P, Shapiro AS, et al. Improving chronic kidney disease detection and treatment in the United States: the chronic kidney disease cascade of care (C3) study protocol. BMC Nephrol. 2022 Oct 12;23(1):331. doi: 10.1186/s12882-022-02943-z. PMID: 36224528; PMCID: PMC9554861.
16. Burton JO, Coats AJS, Kovesdy CP, et al. An international Delphi consensus regarding best practice recommendations for hyperkalaemia across the cardiorenal spectrum. Eur J Heart Fail. 2022 Sep;24(9):1467-1477. doi: 10.1002/ejhf.2612. Epub 2022 Aug 4. Erratum in: Eur J Heart Fail. 2023 Mar;25(3):444. PMID: 35791065; PMCID: PMC9804940.
17. Diamond IR, Grant RC, Feldman BM, et al. Defining consensus: a systematic review recommends methodologic criteria for reporting of Delphi studies. J Clin Epidemiol. 2014 Apr;67(4):401-9. doi: 10.1016/j.jclinepi.2013.12.002. PMID: 24581294.
18. Dalkey N, Helmer O. An experimental application of the Delphi method to the use of experts. Management sci. 1963;9:458-467.
19. Li PK, Garcia-Garcia G, Lui SF, et al. Kidney health for everyone everywhere - from prevention to detection and equitable access to care. Clin Nephrol. 2020 Mar;93(3):111-122. doi: 10.5414/CNWKDEditorial. PMID: 32017699.
20. Komenda P, Ferguson TW, Macdonald K, et al. Cost-effectiveness of primary screening for CKD: a systematic review. Am J Kidney Dis. 2014 May;63(5):789-97. doi: 10.1053/j.ajkd.2013.12.012. Epub 2014 Feb 12. PMID: 24529536.
21. Sammut-Powell C, Sisk R, Budd J, Patel N, Edge M, Cameron R. Development of minimal resource pre-screening tools for chronic kidney disease in people with type 2 diabetes. Future Healthc J. 2022 Nov;9(3):305-309. doi: 10.7861/fhj.2022-0020. PMID: 36561833; PMCID: PMC9761456.
22. Kellum JA, Romagnani P, Ashuntantang G, Ronco C, Zarbock A, Anders HJ. Acute kidney injury. Nat Rev Dis Primers. 2021 Jul 15;7(1):52. doi: 10.1038/s41572-021-00284-z. PMID: 34267223.
23. Schytz PA, Blanche P, Nissen AB, et al. Acute kidney injury and risk of cardiovascular outcomes: A nationwide cohort study. Nefrologia (Engl Ed). 2021 Sep 3:S0211-6995(21)00140-5. English, Spanish. doi: 10.1016/j.nefro.2021.06.007. Epub ahead of print. PMID: 34489123.
24. Thomas M, Magwire M, Gosch K, et al. Cardiometabolic Center of Excellence: A Novel Care Delivery Model for Secondary Prevention of Cardiovascular Disease in Type 2 Diabetes. Circ Cardiovasc Qual Outcomes. 2021 Oct;14(10):e007682. doi: 10.1161/CIRCOUTCOMES.120.007682. Epub 2021 Sep 30. PMID: 34587753; PMCID: PMC8530947.
25. Weber C, Beaulieu M, Djurdjev O, et al. Towards rational approaches of health care utilization in complex patients: an exploratory randomized trial comparing a novel combined clinic to multiple specialty clinics in patients with renal disease-cardiovascular disease-diabetes. Nephrol Dial Transplant. 2012 Oct;27 Suppl 3:iii104-10. doi: 10.1093/ndt/gfr292. Epub 2011 Jun 30. PMID: 21719715.

For peer review only - http://bmjopen.bmj.com/site/about/guidelines.xhtml

26. Spaak J. Novel combined management approaches to patients with diabetes, chronic kidney disease and cardiovascular disease. J R Coll Physicians Edinb. 2017 Mar;47(1):83-87. doi: 10.4997/JRCPE.2017.118. PMID: 28569290.

27. Yang A, Shi M, Lau ESH, et al. Clinical outcomes following discontinuation of renin-angiotensin-system inhibitors in patients with type 2 diabetes and advanced chronic kidney disease: A prospective cohort study. EClinicalMedicine. 2022 Nov 24;55:101751. doi: 10.1016/j.eclinm.2022.101751. PMID: 36457651; PMCID: PMC9706514.

28. Fu EL, Evans M, Clase CM, et al. Stopping Renin-Angiotensin System Inhibitors in Patients with Advanced CKD and Risk of Adverse Outcomes: A Nationwide Study. J Am Soc Nephrol. 2021 Feb;32(2):424-435. doi: 10.1681/ASN.2020050682. Epub 2020 Dec 28. PMID: 33372009; PMCID: PMC8054897.

29. Qiao Y, Shin J, Chen TK, et al. Association Between Renin-Angiotensin System Blockade Discontinuation and All-Cause Mortality Among Persons With Low Estimated Glomerular Filtration Rate. JAMA Intern Med. 2020;180(5):718–726. doi:10.1001/jamainternmed.2020.0193.

30. Clark AL, Kalra PR, Petrie MC, Mark PB, Tomlinson LA, Tomson CR. Change in renal function associated with drug treatment in heart failure: national guidance. Heart. 2019 Jun;105(12):904-910. doi: 10.1136/heartjnl-2018-314158. PMID: 31118203; PMCID: PMC6582720.

31. Morales E, Cravedi P, Manrique J. Management of Chronic Hyperkalemia in Patients With Chronic Kidney Disease: An Old Problem With News Options. Front Med (Lausanne). 2021 Jun 4;8:653634. doi: 10.3389/fmed.2021.653634. PMID: 34150795; PMCID: PMC8213200.

32. Kim GH. Pharmacologic Treatment of Chronic Hyperkalemia in Patients with Chronic Kidney Disease. Electrolyte Blood Press. 2019 Jun;17(1):1-6. doi: 10.5049/EBP.2019.17.1.1. Epub 2019 Jun 30. PMID: 31338108; PMCID: PMC6629599.

33. Clase CM, Carrero JJ, Ellison DH, et al. Potassium homeostasis and management of dyskalemia in kidney diseases: conclusions from a Kidney Disease: Improving Global Outcomes (KDIGO) Controversies Conference. Kidney Int. 2020 Jan;97(1):42-61. doi: 10.1016/j.kint.2019.09.018. Epub 2019 Oct 10. PMID: 31706619.

34. Wang AY. Optimally managing hyperkalemia in patients with cardiorenal syndrome. Nephrol Dial Transplant. 2019 Dec 1;34(Suppl 3):iii36-iii44. Doi: 10.1093/ndt/gfz225. PMID: 31800079.

35. Silva-Cardoso J, Brito D, Frazão JM, et al. Management of RAASi-associated hyperkalemia in patients with cardiovascular disease. Heart Fail Rev. 2021 Jul;26(4):891-896. Doi: 10.1007/s10741-020-10069-3. Epub 2021 Feb 18. PMID: 33599908; PMCID: PMC8149346.

36. Epstein M, Reaven NL, Funk SE, McGaughey KJ, Oestreicher N, Knispel J. Evaluation of the treatment gap between clinical guidelines and the utilization of renin-angiotensin-aldosterone system inhibitors. Am J Manag Care. 2015 Sep;21(11 Suppl):S212-20. PMID: 26619183.

37. Trevisan M, de Deco P, Xu H, et al. Incidence, predictors, and clinical management of hyperkalaemia in new users of mineralocorticoid receptor antagonists. Eur J Heart Fail. 2018 Aug;20(8):1217-1226. Doi: 10.1002/ejhf.1199. Epub 2018 Apr 18. Erratum in: Eur J Heart Fail. 2019 Apr;21(4):540. PMID: 29667759; PMCID: PMC6607478.

38. Fraser SD, Roderick PJ, May CR, et al. The burden of comorbidity in people with chronic kidney disease stage 3: a cohort study. BMC Nephrol. 2015 Dec 1;16:193. doi: 10.1186/s12882-015-0189-z. Erratum in: BMC Nephrol. 2020 Dec 21;21(1):543. PMID: 26620131; PMCID: PMC4666158.

39. Lee WC, Lee YT, Li LC, et al. The Number of Comorbidities Predicts Renal Outcomes in Patients with Stage 3⁻5 Chronic Kidney Disease. J Clin Med. 2018 Nov 28;7(12):493. doi: 10.3390/jcm7120493. PMID: 30486496; PMCID: PMC6306906.

For peer review only

Appendix: Supplementary Data

Figure S1: Proportion of responses for each agreement level by statement

<<Figure S1>>

For peer review only

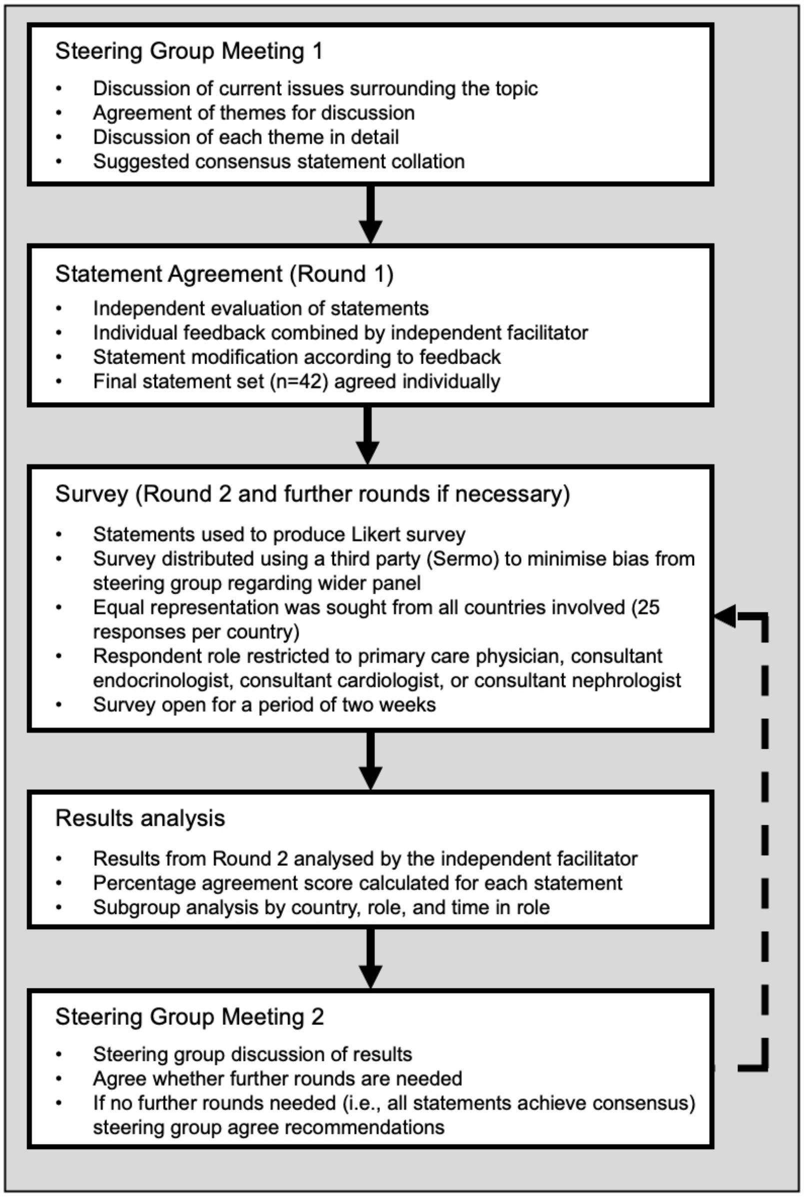

Figure 1: Study flow diagram

282x418mm (72 x 72 DPI)

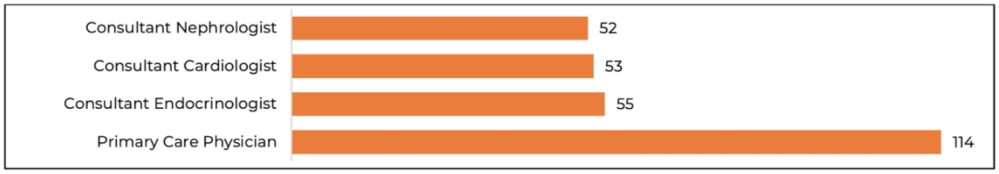

Figure 2: Respondents by role

525x91mm (72 x 72 DPI)

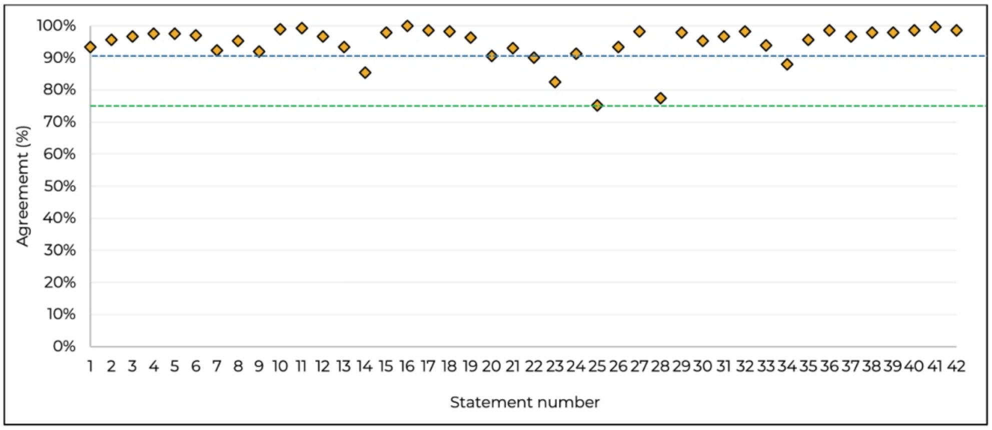

Figure 3: Overall consensus agreement levels by statementa

521x225mm (72 x 72 DPI)

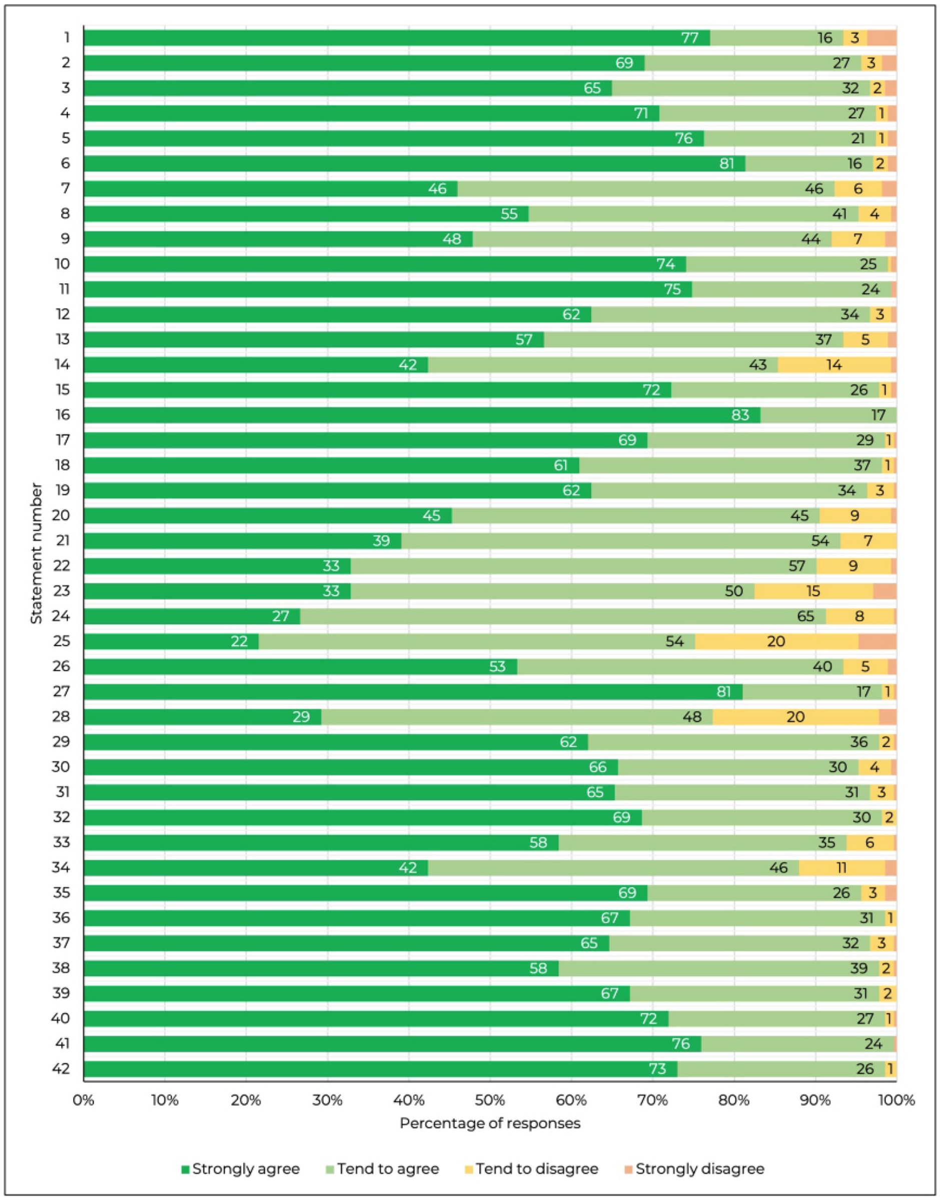

Figure S1: Proportion of responses for each agreement level by statement

306x390mm (72 x 72 DPI)

BMJ Open

Results from a cross-specialty consensus on optimal management of the chronic kidney disease (CKD) patient: from screening to complications

Journal:	*BMJ Open*
Manuscript ID	bmjopen-2023-080891.R1
Article Type:	Original research
Date Submitted by the Author:	11-Jan-2024
Complete List of Authors:	Arici, Mustafa ; Hacettepe Universitesi, ; Hacettepe University Faculty of Medicine<br>Assaad – Khalil, Samir; Alexandria University Faculty of Medicine, Unit of Diabetes, Lipidology & Metabolism<br>Bertoluci, Marcello; Universidade Federal do Rio Grande do Sul Instituto de Biociencias, Endocrinology Division, Hospital de Clínicas de Porto Alegre<br>Choo, Jason; Singapore General Hospital, Department of Renal Medicine<br>Lee, Yau-Jiunn; Lee's Clinic<br>Madero, Magdalena; National Heart Institute of Mexico<br>Rosa Diez, Guillermo ; Hospital Italiano de Buenos Aires, Servicio de Nefrología<br>Sánchez Polo, Vicente ; Instituto Guatemalteco de Seguridad Social, Servicio de Nefrología y Trasplante Renal<br>Chung, Sungjin; The Catholic University of Korea College of Medicine, Division of Nephrology<br>Thanachayanont, Teerawat; Bhumirajanagarindra Kidney Institute, Nephrology<br>Pollock, Carol; University of Sydney
**Primary Subject Heading**:	Renal medicine
Secondary Subject Heading:	Cardiovascular medicine, General practice / Family practice, Global health
Keywords:	Nephrology < INTERNAL MEDICINE, Heart failure < CARDIOLOGY, Hypertension < CARDIOLOGY, Chronic renal failure < NEPHROLOGY, Primary Care < Primary Health Care, Surveys and Questionnaires

SCHOLARONE™
Manuscripts

I, the Submitting Author has the right to grant and does grant on behalf of all authors of the Work (as defined in the below author licence), an exclusive licence and/or a non-exclusive licence for contributions from authors who are: i) UK Crown employees; ii) where BMJ has agreed a CC-BY licence shall apply, and/or iii) in accordance with the terms applicable for US Federal Government officers or employees acting as part of their official duties; on a worldwide, perpetual, irrevocable, royalty-free basis to BMJ Publishing Group Ltd ("BMJ") its licensees and where the relevant Journal is co-owned by BMJ to the co-owners of the Journal, to publish the Work in this journal and any other BMJ products and to exploit all rights, as set out in our licence.

The Submitting Author accepts and understands that any supply made under these terms is made by BMJ to the Submitting Author unless you are acting as an employee on behalf of your employer or a postgraduate student of an affiliated institution which is paying any applicable article publishing charge ("APC") for Open Access articles. Where the Submitting Author wishes to make the Work available on an Open Access basis (and intends to pay the relevant APC), the terms of reuse of such Open Access shall be governed by a Creative Commons licence – details of these licences and which Creative Commons licence will apply to this Work are set out in our licence referred to above.

Other than as permitted in any relevant BMJ Author's Self Archiving Policies, I confirm this Work has not been accepted for publication elsewhere, is not being considered for publication elsewhere and does not duplicate material already published. I confirm all authors consent to publication of this Work and authorise the granting of this licence.

For peer review only

Results from a cross-specialty consensus on optimal management of the chronic kidney disease (CKD) patient: from screening to complications

Arici M M.D.[1], Assaad-Khalil S M.D.[2], Bertoluci MC M.D.[3], Choo JCJ M.B.B.S.[4], Lee Y-J M.D.[5], Madero M M.D.[6], Rosa-Diez G M.D.[7], Sánchez-Polo V M.D.[8], Chung S M.D.[9], Thanachayanont T M.D.[10], Pollock C M.B.B.S.[11]

1. Division of Nephrology, Department of Internal Medicine, Faculty of Medicine, Hacettepe University, Ankara, Turkey
2. Unit of Diabetes, Lipidology & Metabolism; Department of Internal Medicine/ Alexandria Faculty of Medicine, Alexandria University, Alexandria, Egypt
3. Endocrinology Division, Hospital de Clínicas de Porto Alegre, Universidade Federal do Rio Grande do Sul, Porto Alegre, Brazil
4. Department of Renal Medicine, Singapore General Hospital, Singapore
5. Lee's Endocrinology Clinic, Pingtung 90000, Taiwan
6. Division of Nephrology, Department of Medicine, National Heart Institute, Mexico City, Mexico
7. Servicio de Nefrología, Hospital Italiano de Buenos Aires, Buenos Aires, Argentina
8. Nephrology Department, Guatemalan Social Security Institute (IGSS)
9. Department of Internal Medicine, College of Medicine, The Catholic University of Korea, Seoul, Republic of Korea; Clinical Practice Guideline Committee, The Korean Society of Nephrology
10. Bhumirajanagarindra Kidney Institute, Bangkok Thailand
11. Kolling Institute of Medical Research, Sydney Medical School, University of Sydney, Royal North Shore Hospital, St Leonards, NSW, Australia

Correspondence to:

Professor M Arici

Division of Nephrology, Department of Internal Medicine, Faculty of Medicine,
Hacettepe University, Istanbul, Turkey
Division of Nephrology, Department of Internal Medicine,

Faculty of Medicine, Hacettepe University,

Ankara, Turkey

marici@hacettepe.edu.tr

Main text = 4,044 words

For peer review only

KEYWORDS

- Chronic Kidney Disease
- Heart Failure
- Hypertension
- Hyperkalaemia
- Primary Care
- Multidisciplinary Care Team

For peer review only

ABSTRACT (297 WORDS)

Background: Chronic kidney disease (CKD) affects around 10% of the global population and has been estimated to affect around 50% of individuals with type 2 diabetes and 50% of those with heart failure. The guideline recommended approach is to manage with disease-modifying therapies, but real-world data suggests that prescribing rates do not reflect this in practice.

Objective: To develop a cross-specialty consensus on optimal management of the CKD patient using a modified Delphi method.

Design: An international steering group of experts specialising in internal medicine, endocrinology/diabetology, nephrology and primary care medicine developed 42 statements on aspects of CKD management including identification and screening, risk factors, holistic management, guidelines, cross-specialty alignment, and education. Consensus was determined by agreement using an online survey.

Participants: The survey was distributed to cardiologists, nephrologists, endocrinologists, and primary care physicians across 11 countries.

Main outcomes and measures: The threshold for consensus agreement was established *a priori* by the steering group at 75%. Stopping criteria were defined as a target of 25 responses from each country (N=275), and a four-week survey period.

Results: 274 responses were received in December 2022, 25 responses from Argentina, Australia, Brazil, Guatemala, Mexico, Singapore, South Korea, Taiwan, Thailand, Turkey, and 24 responses from Egypt. 53 responses were received from cardiologists, 52 from nephrologists, 55 from endocrinologists, and 114 from primary care physicians. 37 statements attained very high agreement (≥90%) and 5 attained high agreement (≥75% and

<90%). Strong alignment between roles was seen across the statements, and different levels of experience (2-5 years or 5+ years), some variation was observed between countries.

Conclusions: There is a high degree of consensus regarding aspects of CKD management among healthcare professionals. Based on these strong levels of agreement, the steering group derived twelve key recommendations focused on diagnosis and management of CKD.

STRENGTHS AND LIMITATIONS OF THIS STUDY

- 274 responses received with good representation from experienced physicians across 11 different countries
- Majority of responses from physician with more than ten years' experience in role, suggesting that the results represent the views of experienced physicians.
- Very high levels of agreement may suggest either that the statements were constructed to achieve agreement (confirmation bias), or that they represent already recognised good practice.
- Lack of representation from low-income countries, e.g., sub-Saharan Africa is a limitation.
- Aspects of patient choice and empowerment and consideration of the patient experience (outside of treatment outcomes) have not been discussed.

For peer review only

WORD COUNT = 4044

BACKGROUND

Chronic kidney disease (CKD) is defined as abnormalities of kidney structure or function, which are present for >3 months.[1] This is indicated by a glomerular filtration rate (GFR) below 60 mL/min/1.73 m², or the presence of one or more markers of kidney damage. CKD is classified based on cause, glomerular filtration rate category, and albuminuria category.[1] Diabetes is the leading cause of CKD, followed by hypertension, suggesting a close relationship between the cardio-renal-metabolic systems.[2] The interplay between CKD, cardiovascular disease (CVD), and metabolic diseases such as type 2 diabetes mellitus (T2DM) is significant and has been termed 'cardio-renal-metabolic' (CRM) disease.[3] It has been suggested that CKD affects around 50% of people with T2DM [4], around 50% of heart failure (HF) patients [5], and up to 38% of those with hypertension [6], suggesting the need for a holistic approach to management of CKD patients.

Between 1990 and 2017, the prevalence of CKD was estimated to rise to 9.1% of the global population, with associated mortality increasing by 41.5%.[7] This is closely associated with an increase in populations with risk factors such as diabetes, hypertension, and prediabetes.[2] The individual burden of CKD falls disproportionately upon those with lower socioeconomic status. This population is associated with a higher prevalence of CKD and variable access to treatment in some health economies, and ultimately poorer outcomes. In low- and middle-income countries (LMICS), an estimated 188 million people experience catastrophic health expenditure annually due to kidney diseases, the greatest of any disease group.[8] Once diagnosed, individuals with CKD in LMICs may have limited access to health care due to lack of public funding, insurance coverage, and affordability. As a consequence, in 2014 it was estimated that globally only 10% of individuals with end stage kidney disease receive renal replacement therapy, and over 1 million people die annually from untreated kidney disease.[9]

Early diagnosis and referral of CKD are key to reducing or avoiding progression to kidney failure, and reducing morbidity and mortality. The absence of symptoms in early stages of CKD requires that clinicians maintain suspicion in all patients and especially those with risk-factors.[10] Accurate screening, diagnosis, and risk stratification need to be in place to support the aim of 'earliest diagnosis'.

A diagnosis of CKD is determined by laboratory confirmation of proteinuria or haematuria, and/or a reduction in the glomerular filtration rate (GFR), for more than 3 months' duration.[11] Two key markers of CKD include albuminuria (defined as a urinary albumin-to-creatine ratio [uACR] of ≥30 mg/g) and reduction in estimated GFR (eGFR), as calculated by the CKD-EPI based on creatinine alone ($eGFR_{cr}$) or on creatinine and cystatin C ($eGFR_{cr\text{-}cys}$).[11,12]

For peer review only - http://bmjopen.bmj.com/site/about/guidelines.xhtml

In practice, these methods may not be utilised effectively, an analysis of over 123,000 patient records found low frequency of uACR testing in patients with CKD, despite strong epidemiological evidence linking increased albuminuria with disease progression, kidney failure, cardiovascular events, and premature mortality.[13]

Besides lifestyle modifications, disease-modifying therapies (DMTs) for CKD including renin-angiotensin-aldosterone system inhibitors (RAASi), and sodium-glucose co-transporter-2 inhibitors (SGLT2i), as recommended by Kidney Disease Improving Global Outcomes (KDIGO) for use in CKD patients with hypertension or diabetes, and non-steroidal mineralocorticoid antagonists (ns-MRAs) as add-on therapy in patients with T2DM and residual albuminuria.[1,14] Despite the availability of these therapies, analysis of 2 large US healthcare systems showed that even though nearly two-thirds of the adults with CKD had diabetes, hypertension, or prediabetes, rates of prescribing RAASi were low. Not only was DMT use below guideline directed levels, but potentially nephrotoxic agents (such as non-steroidal anti-inflammatory drugs and proton pump inhibitors) were used more commonly than RAASi.[2]

There is clearly some disconnect between guideline recommendations and real-world practice. Guidelines for the management of CKD may not be clear or made known to primary care practitioners (PCPs). Furthermore, applicability or ease of implementation may be questionable in LMICs.[15,16]

The objective of this project is to use a modified Delphi technique to examine the opinions of healthcare professionals towards aspects of CKD management across 11 countries, report these findings, and develop practical recommendations for diagnosis and management of CKD.

METHODS

A steering group of experts (see author list) from 11 different countries convened in September 2022 to discuss current challenges in CKD management. The experts were defined as specialists in nephrology, endocrinology/diabetology, internal medicine, and primary care medicine who had achieved an appropriate level of seniority within their field (e.g., professors or associate professors), or had published papers related to the management of CKF/HF/Diabetes, or had been involved in guidelines development.[17-25] The steering group members were selected to provide representation across a range of development indicator levels external to Europe and North America (to avoid replication of previous published work [26]).

For peer review only

The Delphi technique used in this study was guided by Guidance on Conducting and REporting DElphi Studies (CREDES). Whilst guidelines exist, outcomes for CKD vary globally, and are particularly poor in LMICs, to understand the barriers and opportunities, a modified Delphi approach was employed to understand where common care processes differ in local use, and how the attitudes of healthcare professionals to elements of CKD management differ between countries. The overall process is outlined in Figure 1.

Figure 1: Study flow diagram

<<Figure 1>>

Six themes for focus for statement development were agreed (Table 1), these were discussed further, and statements developed by the Steering Group working collaboratively. The statements were then collated, and the steering group independently rated the statements as either “accept”, “remove”, or “reword” with suggested changes (as determined by a simple majority) with the potential for further group consultation for any significant differences of opinion on the fundamental principles of any statement. Once finalised, the steering group were agreed the final set of statements for testing. This constituted the initial round of consensus.

The resulting 42 statements were developed into a *Likert* survey, this was then distributed by a third party (Sermo) in Round 2 of the process.

Panel members were recruited based on the following criteria:

- Employed within one of the 11 target countries.
- 25 respondents from each country in a broadly 2:1:1:1 ratio of Primary Care Physician, Consultant Endocrinologist, Consultant Cardiologist, Consultant Nephrologist (or local equivalent).

The identity of respondents was not known to the steering group or the independent facilitator. For each statement, respondents were offered a 4-point scale (‘strongly disagree’, ‘tend to disagree’, ‘tend to agree’, and ‘strongly agree’) to indicate their level of agreement with each statement. The survey also captured country, specialty, length of time in role, and average number of CKD patients managed over 3 months. Stopping criteria for data collection were defined as a target of 25 responses from each country (N=275), and a four-week survey period.

The target countries were chosen to reflect the Steering Group demographic, this would enable each Steering Group member to provide insight into results from their respective country. Panel members were recruited from the

For peer review only

following countries: Argentina (UMI), Australia (HI), Brazil (UMI), Egypt (LMI), Guatemala (UMI), Mexico (UMI), Singapore (HI), South Korea (HI), Taiwan (HI), Thailand (UMI), and Turkey (UMI)[i].

The closing criteria for the study was defined *a priori* in line with best practice principles as: 90% of the final statement set achieving consensus threshold (defined at 75%, a widely accepted threshold).[27] If these criteria were not met, statements would be modified, and the survey re-issued as necessary for a maximum of three rounds. Consensus was further categorised as 'high' at ≥75% and 'very high' at ≥90%. Completed surveys were analysed using Microsoft Excel software. The responses were aggregated to provide an overall agreement level (i.e., the number of responded expressing agreement as a percentage of the overall number of responses for each statement).

As this study only sought the anonymous opinions of physicians and no patient-specific data were captured, ethical approval was not sought. Respondents provided consent to have their responses included in this study through their inclusion to the panel held by Sermo.

Patient and Public Involvement

None, the stated objective was to examine the opinions of healthcare professionals towards aspects of CKD management across 11 countries.

Results

Of the 45 initial statements created by the Steering Group, 32 were retained, 8 were modified, 5 were removed, and 3 new statements were added. The final set was then sent out to the group via email for acceptance prior to progressing to Round 2.

In Round 2, completed surveys from a panel of 274 were analysed to define the total level of agreement with each of the 42 statements. Respondents were either PCPs or consultants in cardiology, nephrology, and endocrinology (including diabetology) (Figure 2). Most responders had more than 10-years' experience in role (159/274) and only 13% (n=35) had less then 5 years in role. When asked to estimate the number of patients seen in a 3-month

[i] HI – High Income, LMI – Lower-Middle Income, UMI – Upper-Middle Income, according to World Bank Classification.

For peer review only

period, 177 responders (65%) stated that they saw more than 50 patients (either as inpatients or outpatients). This suggests the respondent cohort was sufficiently experienced to provide insight into aspects of CKD.

Figure 2: Respondents by role

<<Figure 2>>

Consensus was achieved for all statements, with very high agreement (≥90%) in 37 (88%) statements, and high agreement (≥75 & ≤89%) in 5 (12%) statements. Overall consensus agreement by statement is shown in Figure 3, and Table 1, a detailed results showing percentages of agreement at each level can be found in supplementary table S1 and figure S1.

Figure 3: Overall consensus agreement levels by statement[a]

<<Figure 3>>

[a]The green horizontal line represents the 75% threshold for consensus agreement and the blue line indicates the threshold for very high consensus (90%).

Table 1. Agreement to each statement, divided by topic.

No:	Statement:	Overall
**Earlier identification & screening of CKD**		
**1.**	Late diagnosis is a lost opportunity for early management of CKD which can slow disease progression	**93%**
**2.**	National screening and diagnostic programmes for CKD are essential to identifying patients at earlier stages of CKD	**96%**
**3.**	A simple and practical definition of 'high-risk' should be established to support cost-effective screening	**97%**
**4.**	Early screening for CKD in high-risk groups is cost-effective for the health system (where resources are in place to support intervention)	**97%**
**5.**	Primary care physicians and specialists (cardiologists, endocrinologists, nephrologists, diabetologists, etc.) should routinely screen for CKD in patients with risk factors	**97%**
**6.**	Patients with risk factors (e.g., elderly, diabetes, hypertension, dyslipidaemia, obesity, family history of CKD) should be screened at least annually for CKD	**97%**
**7.**	Patients with CKD should be screened for heart failure (including those with preserved ejection fraction (HFpEF))	**92%**
**8.**	GFR estimated by the CKD-EPI Creatinine Equation (2021) and albuminuria (using albumin-to-creatinine ratio) should be the screening method of choice for CKD	**95%**
**9.**	Confirmatory testing of abnormalities of kidney function or structure at 3 months is required to establish a CKD diagnosis	**92%**
**Risk factors for CKD in cardio-renal-metabolic patients**		
**10.**	A holistic view of cardio-renal-metabolic disease states is critical to provide integrated patient-centred care to individuals with CKD	**99%**
**11.**	CKD progressively increases the risk of CVD, including heart failure and overall mortality	**99%**
**12.**	Frequency of hyperkalaemia increases as CKD progresses	**97%**
**13.**	AKI is a risk factor for CKD and chronic heart failure	**93%**
**14.**	Infection, including COVID-19, can cause AKI and is therefore a risk factor for CKD	**85%**
**15.**	Nephrotoxic medications should be modified, and risk/benefit profiles should be assessed in patients with, or at risk of CKD	**98%**

For peer review only

**Holistic management of CKD in cardio-renal-metabolic patients**		
**16.**	Lifestyle factors such as diet, smoking, exercise must be optimised for CKD patients	**100%**
**17.**	Disease modifying therapies for CKD (e.g., SGLT2i, RAASi, MRA) should be used where indicated to manage progression of CKD	**99%**
**18.**	SGLT2i and RAASi have a complementary cardio-renal protective action	**98%**
**19.**	Early use of SGLT2i could prevent the development and progression of CKD patients with type 2 diabetes and heart failure	**96%**
**20.**	Early use of SGLT2i can slow progression of CKD in patients without diabetes	**91%**
**21.**	A small decrease of eGFR (~10%) may be expected on initiation of CKD disease-modifying therapies	**93%**
**22.**	De-escalation or discontinuation of RAASi therapy is associated with worse cardiovascular and renal outcomes in the CKD patient	**90%**
**23.**	Disease modifying therapies for CKD should only be stopped as a last resort	**82%**
**24.**	Novel K binders (i.e., patiromer and sodium zirconium cyclosilicate) are an option to manage hyperkalaemia and prevent de-escalation or downtitration of RAASi	**91%**
**25.**	Action to manage hyperkalaemia in the CKD patient should be taken when serum potassium level reaches 5.0mmol/L	**75%**
**26.**	Action to manage hyperkalaemia in the CKD patient should be taken when serum potassium level reaches 5.5 mmol/L	**93%**
**27.**	Treatment targets such as blood pressure, blood glucose, lipid levels should be maintained for both kidney and cardiac outcomes in CKD patients	**98%**
**Guidelines**		
**28.**	Implementation of evidence-based CKD guidelines is suboptimal and should be improved	**77%**
**29.**	Cardiology, nephrology, and endocrinology guidelines for CKD should be aligned	**98%**
**30.**	Primary care physicians have an important role in implementing guidelines	**95%**
**31.**	Guidelines should be practical with an executive summary / checklist to assist implementation by non-specialist HCPs	**97%**
**32.**	Guidelines should include clear criteria for when and how to refer to other specialists/MDT	**98%**
**33.**	Guidelines should reflect differences in population phenotypes and be locally adapted where needed	**94%**
**34.**	Patient perspective is crucial in developing guidelines	**88%**
**35.**	Investment is needed to shift the approach to primary prevention rather than end stage kidney disease management	**96%**
**Cross-specialty alignment (Cardiology, Nephrology, Endocrinology, Primary Care & Policy Makers)**		
**36.**	Patients with CKD should be managed by a multi-disciplinary team (MDT) where possible with an agreed management plan	**99%**
**37.**	The CKD multi-disciplinary team (MDT) should include the primary care physician to improve early intervention and decision making	**97%**
**38.**	The care pathway for CKD should be designed to minimise the impact of organisational barriers	**98%**
**39.**	Clinicians, professional associations, academic institutions, and patient representative organisations need to engage with policy makers to ensure appropriate plans and funding are in place to deliver optimal CKD care	**98%**
**Education of clinicians and patients**		
**40.**	Up-to-date healthcare professional (HCP) education on the management of patients with CKD and associated guidelines is needed	**99%**
**41.**	Structured education of primary care physicians on screening and early detection improves patient outcomes	**100%**
**42.**	Therapeutic patient education is key for them to understand the consequences of CKD and how to manage it through lifestyle modification and appropriate use of therapies	**99%**

As none of the statements failed to reach the predetermined threshold of 75%, only one round of survey was required. The results of the survey represent current opinions of the respondents and are not intended to contradict the established evidence base. Anonymized Round 2 results data are available in supplemental table S2.

For peer review only

DISCUSSION (Note, in the discussion below, 'S' is used to denote 'statement'.)

Earlier identification & screening of CKD (S1-9)

Very high agreement (>90%) was observed for all statements in this topic, underscoring the key principle that early diagnosis of CKD is key to implementing strategies to slow disease progression. Responses suggest a strong support for the need for national kidney health screening and diagnostic programmes. Universal screening of the general population has been found not to be cost-effective [28], but systematic review has shown screening to be cost-effective in patients with hypertension or diabetes.[29] Indeed, a KDIGO Controversies Conference concluded that targeted groups, such as those with hypertension, diabetes, or cardiovascular disease should be screened for CKD, and that an individualised approach should be taken to screen others based on a range of factors.[8]

Respondents agree that patients with risk factors should be screened for CKD at least annually by using eGFR and uACR where available. Low- and middle-income countries (LMICs) are disproportionately affected by CKD, with a prevalence around 15% higher than for high income countries.[30] CKD prevalence in sub-Saharan Africa is four times higher than that of North Africa. The incidence of CKD in low-income countries is not only linked to the growing prevalence of well-recognised risk-factors such as obesity, T2DM, hypertension, but also the increased incidence of communicable risk-factors such as streptococcal infections, schistosomiasis, leishmaniasis, and HIV. Additionally, some populations may have a higher genetic risk for kidney disease.[31] These risk factors must all be considered (where relevant) when identifying high-risk individuals.

The recommendations proposed in this report require resource and investment in primary care services within LMICs. The ISN-Global Kidney Health Atlas 2023 [32] reports that the majority of primary care services in Africa and South Asia are not able to provide basic diagnostic services such as serum creatinine assessment. The issue of identification is also compounded in low-income countries by lack of awareness amongst healthcare staff and poor health literacy in the general population.[31] In this context, earlier detection and intervention is of even greater importance in LMICs where the costs of dialysis may be prohibitive for many.

A minimal-resource pre-screening tool has been developed and globally validated for CKD in people with T2DM. This demonstrates that age, gender, body mass index, duration of diabetes, and blood pressure information can be used to identify those at an increased risk of CKD.[33] This model does not require sophisticated diagnostics and can be used to guide cost-effective screening for CKD where resources are limited.

For peer review only

Risk factors for CKD in cardio-renal-metabolic patients (S10-15)

All statements in this topic achieved >90% consensus apart from statement 14 (85%). This is interesting and could be due to the wording of the statement and the specific use of COVID-19 as an example. Reference to COVID-19 was included in the statement as it was considered relevant by the steering group at the time of statement generation and evidence was beginning to emerge of potential kidney injury associated with COVID-19. Analysis by country found that Taiwan agreement was at 68% for this statement, noticeably lower than for other countries. Although 93% of respondents agree that AKI is a risk-factor for CKD and HF, it is interesting that 7% do not agree given the evidence base.[34,35]

Given the established interplay between the cardiac, renal and endocrine systems, it is heartening to see that 99% of respondents agree that a holistic approach is needed to provide personalised care for individuals with CKD.

Where practical, models of care should be developed to deliver integrated multidisciplinary care to patients with cardio-renal-metabolic disease, as described by the CardioMetabolic Care Alliance, to use comprehensive, patient-centred, team-based approaches for aggressive secondary prevention. [36] Implementing combined clinics to deliver medical care for patients with kidney disease and diabetes or CVD may reduce outpatient healthcare costs without compromising health outcomes.[37] Combined multidisciplinary clinics for diabetes, CKD, and CVD are also associated with a slower decline in GFR than usual care, and a significant reduction in the risk for all-cause mortality.[38] However, global variation exists regarding resource availability and access to specialist care [32], and the use of MDTs is not universal. In these regions, HCPs are encouraged to develop local contacts/networks to allow for inter-disciplinary discussions of CKD cases where needed.

Holistic management of CKD in cardio-renal-metabolic patients (S16-27)

In lower resource settings the availability of DMT to manage CKD may be a challenge, but the broad objective of this consensus is to promote the earlier detection and management of CKD, and this principle applies regardless of resource limitations. As CKD associated health care costs increase with disease progression [39], the economic argument supports this approach, and in patients with limited access to specialist care (including DMT), the role of patient education is paramount in improving health literacy and promoting lifestyle changes to reduce CKD risk. Coupled with this, the health system should look at how interventions such as DMTs can be used to slow progression of CKD and the need for dialysis (and associated costs), cost-savings from slower progression to dialysis dependence might be reinvested in detection and diagnosis/national screening of high-risk patient groups.

For peer review only

Very high agreement was observed for 10 of the statements in this topic, with two statements at lower levels of agreement (S23 and S25, 82% and 75%, respectively). Response to statement 23 is interesting, given the current debate regarding the benefits of stopping RAASi in advanced CKD. Results from a 52-week open-label UK study in patients with CKD stages 4-5 concluded 'The STOP-ACEi trial did not find any benefit by stopping RAASi in advanced CKD', and that 'stopping RAASi in advanced CKD at an arbitrary GFR threshold is not the optimal approach'. Large observational studies have confirmed the cardioprotective benefits of RAASi in advanced CKD (including patients with concomitant T2DM).[40-42] Linked to this is statement 21, respondents agree that a small decrease in eGFR is to be expected upon initiation of DMT for CKD. During initiation and up-titration of RAASi treatment, a decline in kidney function of up to 30% within 4 weeks can be acceptable [1,43] and it is important to avoid a knee-jerk response and reduce or stop DMT, and consultation with a Nephrologist or multidisciplinary team (MDT) is recommended.

Hyperkalaemia (HK) is potentially life-threatening; it may be acute or chronic and individuals with CKD are at an increased risk, which increases with the later stages of CKD.[44] HK treatment is often stratified by serum potassium level, with borderline levels warranting modification of dietary potassium intake, followed by pharmaceutical management for larger or more sustained increases.[45] Evidence to support recommending low potassium diets to patients with advanced CKD or ESRD is weak. Observational studies report weak associations between dietary potassium intake and potassium concentration and this approach may deprive patients of the beneficial cardiovascular effects associated with potassium-rich diets.[46]

When HK occurs, RAASi dose reduction or discontinuation are the most common utilized therapeutic options, but this approach is associated with worse cardiorenal outcomes and increased mortality.[47-49] Once stopped, RAASi treatment is rarely reinitiated.[50] In cases of mild-to-moderate hyperkalaemia, DMT should be maintained where possible [1,14], and an option for achieving this is using a potassium lowering therapy such as patiromer or sodium zirconium cyclosilicate (S24, 91%).

Whilst the threshold for intervention in HK may vary by country and even individual physician, we can conclude that action may be considered when K^+ concentration reaches 5.0 mmol/L (S25, 75%) and certainly when at 5.5 mmol/L (S26, 93%) but with a degree of personalisation (in consultation with a nephrologist). Statements 25 and 26 were included to try and understand where the weight of opinion fell regarding interventions to manage hyperkalaemia, both statements achieved consensus, but the strongest agreement was for statement 26. On reflection, these

For peer review only

statements could have been worded better to understand what would constitute appropriate action in these circumstances.

Respondents very strongly agree that SGLT2i and RAASi therapies have a complementary cardio-renal protective action (S18, 98%) and that in patients with T2DM and HF, early SGLT2i use can prevent the development and progression of CKD (S19, 96%), in fact, SGLT2i may slow progression of CKD in patients without T2DM (S20, 91%). It is therefore recommended that SGLT2i and RAASi therapies are initiated as early as possible to both delay CKD progression and to therapeutically address the full cardio-renal-metabolic continuum – including T2DM and HF.

Guidelines (S28-35)

The interplay between CKD, diabetes, and CVD have been discussed, but CKD is associated with a number of comorbidities. A prospective UK cohort study of 1,741 people with CKD stage 3 found that isolated CKD (without comorbidities) was present in only 4% of patients, and that common comorbidities include ‘painful condition’ (30%), anaemia (24%), thyroid disorders (12%), cerebrovascular disease (12%), and respiratory conditions (10%). These comorbidities should be identified and managed as part of a multidisciplinary approach.[51]

Whilst all statements in this topic achieved consensus threshold, the steering group were surprised that 23% of respondents did not agree that implementation of CKD guidelines is suboptimal. The lowest agreement levels were observed in South Korea (68%) and Guatemala (72%). This could be either genuine strength in implementation of guidelines in these countries (which should be investigated and replicated elsewhere), or the need for continuing professional development (CPD) even amongst experienced physicians.

Due to the vital role of primary care must play in identifying and referring suspected CKD patients, respondents agree that clear guidelines for referral of patients are considered essential (S32, 98%). PCPs should be provided with clear criteria for referral and followed through with continuing education from an appropriate specialist (ideally a nephrologist). The focus on prevention can only be achieved through a whole-system approach with appropriate investment in awareness, screening, and diagnosis programmes (S35, 96%).

For peer review only

Cross-specialty alignment (Cardiology, Nephrology, Endocrinology, Primary Care & Policy Makers) (S36-39)

Very high levels of agreement suggest strong support for multidisciplinary involvement in CKD management, and that PCPs should form part of the MDT. Given the prominent status of CKD as an emerging global health threat [52], there is a need to ensure that plans and funding are in place to enable optimal care. Stakeholders should engage with both local and national policymakers to ensure that CKD is appropriately prioritised. Policy makers should consider how access to CKD related healthcare (including affordability of medicines) can be improved in LMICs, and governmental and regulatory bodies should consider how to engage best with the pharmaceutical industry to ensure that limited funding is used to maximal effect.

Education of clinicians and patients (S40-42)

Most responders agree on the need for up-to-date education for all healthcare professional roles involved in CKD management, and that PCPs require structured education. Education of patients is a low-cost intervention, particularly in areas with poor health literacy (LMICs) to encourage kidney healthy lifestyles and improve awareness of CKD among high-risk populations.

Strengths and limitations

274 responses received with good representation from experienced physicians across 11 different countries is a strong basis for consensus, the study was designed to ensure that each country was represented by an equal number of responders. The majority of responders reported more than 10 years' experience in role (159/274), suggesting that the results represent the views of experienced physicians.

This study had some key limitations. Very high levels of agreement were achieved across the statements, this which may suggest that the statements were constructed as to achieve agreement (confirmation bias), or that perhaps they represent established good practice, a possible improvement to the process might be to provide survey responders (or a sub-set) with the opportunity to add or amend the statements prior to the full survey. It must also be acknowledged that associations with pharmaceutical companies may have led to unconscious bias the development of the consensus statements, but the study was designed to minimise the impact of this bias on results: the identity of steering group members was not disclosed to survey responders, and the identity of responders was not known to either the facilitator or the steering group.

For peer review only

Whilst the survey covered 11 countries with different economic status, the range was from lower-middle income to high, there is a clear gap in this data –countries classified as ‘low income’ (e.g., Sub-Saharan Africa). We must recognise that implementation of recommendations may not be possible or practical in areas not represented by the dataset. Future work should focus explore the situation in low-income countries, this could provide insight into the specific challenges and considerations and allow for comparison with the current dataset.

Aspects of patient choice and empowerment and consideration of the patient experience (outside of treatment outcomes) have not been discussed, this can be considered a limitation as the patient perspective may have significant bearing on the practicability and implementation of CKD management.

RECOMMENDATIONS

1. Early screening for CKD in high-risk groups is cost-effective for the health system (where resources are in place to support intervention)
2. GFR (estimated by the CKD-EPI Creatinine Equation) and uACR (using albumin-to creatinine ratio) should be the screening method of choice for CKD
3. The CKD multi-disciplinary team (MDT) should include the primary care physician to improve early intervention and decision making
4. Intervene early in CKD patients with proven therapies (i.e., SGLT2i, RAASi, DMTs) to delay/prevent progression to kidney failure
5. RAASi therapies should be optimised in all CKD and HF patients
6. Patients with CKD should have their hyperkalaemia managed appropriately when serum potassium level rises above >5.0 mmol/L
7. Chronic hyperkalaemia should be treated with a potassium binder (e.g., patorimer, sodium zirconium cyclosilicate) to allow for the maintenance of disease-modifying therapies
8. Guidelines should be practical with an executive summary/checklist to assist implementation by non-specialist HCPs
9. Guidelines should include clear criteria for when and how to refer to other specialists/MDT
10. Nephrologists should work together to deliver consistent and clear education regarding CKD management
11. Clinicians, professional associations, academic institutions, and patient representative organisations need to engage with policymakers to ensure appropriate plans and funding are in place to deliver optimal CKD care

For peer review only

12. National kidney health programmes should be implemented to drive improvements to screening and diagnosis

CONCLUSION

The Steering Group was able to form a set of recommendations based on the high levels of agreement achieved for most statements. Implementation of these recommendations has the potential to improve detection of CKD at an earlier stage in patients with risk factors. Earlier diagnosis provides the opportunity for early intervention with DMTs that can slow or halt the progression of CKD, in addition to reducing associated morbidity and mortality.

DECLARATIONS

Data availability statement

All data relevant to the study are included in the article or uploaded as supplementary information.

Ethics statements

Patient consent for publication

Not applicable.

Ethics approval

Not applicable.

Author contributions: All views expressed by the authors are those of the individual and do not represent those of their organisation/place of work. Contributors MA, SAK, MCB, JCJC, YJL, MM, GRD, VSP, SC, TT, CP agreed the design of the study, formulated and reviewed the statement set. Results of the study were discussed by all contributors as part of a formal Steering Group meeting where commentary was agreed for development into the initial draft manuscript. MA, SAK, MCB, JCJC, YJL, MM, GRD, VSP, SC, TT, CP took an equal role in reviewing the initial manuscript draft and providing comments, and all approved the final draft. MA is the guarantor for the work and/or conduct of the study, had access to the data and controlled the decision to publish.

Acknowledgements: The authors would like to thank Ian Walker and Dal Singh of Triducive Partners Limited for their assistance in survey distribution, analysis of result, writing the initial draft manuscript, and reviewing the final draft.

For peer review only

FUNDING

The study was initiated and funded by AstraZeneca. All authors received funding from AstraZeneca while undertaking this study. AstraZeneca commissioned Triducive Partners Limited to facilitate the project and analyse the responses to the consensus statements in line with the Delphi methodology. After engaging Triducive Partners Limited, AstraZeneca made no contribution to the design and development of the study outside of payment of honoraria. AstraZeneca took no part in the writing, revision or editing of the manuscript except to check that the manuscript contained no promotion of specific medicines and that all recommendations were appropriate to drug label.

CONFLICTS OF INTEREST

M Arici has received payment honoraria for lectures, presentations, speaker bureaus, publication writing or educational events from: Amgen, AstraZeneca, Bayer, Boehringer Ingelheim, Menarini, MSD, Novo Nordisk, Sandoz, and Sanofi. M Arici has also participated in a Data Safety Monitoring Board or Advisory Boards for*:* AstraZeneca, Bayer, Boehringer Ingelheim, and Novo Nordisk. MC Bertoluci has worked as speaker for: Aché, AstraZeneca, Bayer, Boehringer-Ingelheim, Novo-Nordisk, and Servier. JCJ Choo has consulted for, or been on advisory boards for: AstraZeneca, ATC, Bayer, GSK, Nitto Denko, and Pfizer. JCJ Choo has been a speaker for: Abbott, AstraZeneca, Bayer, and Boehringer Ingelheim. JCJ Choo has been on Steering Committees or Data Safety Monitoring Boards for: AstraZeneca, Boehringer Ingelheim, and Novartis, and has received scientific grant funding from: AstraZeneca, ATC, and Nitto Denko. Y-J Lee has been a speaker and Advisory Board member for: AstraZeneca Taiwan. M Madero was the International Society for Nephrology Deputy Chair for Latin America and Caribbean (2019-2023) and is currently the on the council for the International Society for Peritoneal Disease (2022-2024). M Madero has been a consultant for: AstraZeneca, Bayer, and Boehringer Ingelheim, and provided expert testimony for: AstraZeneca, Bayer, and Boehringer Ingelheim. M Madero has received grants from or has pending grants with: AstraZeneca*, Bayer*, Boehringer, Renal Research Institute*, and Tricida*. M Madero has received travel grants from: AstraZeneca and Medcom. GJ Rosa Diez has been a speaker or Advisory Board member for: Astra Zeneca, Boehringer Ingelheim, Bayer, Fresenius, and Medtronic. S Chung has received honoraria as a speaker or advisory board member from: AstraZeneca, Boehringer Ingelheim, Daewon, Daewoong, Eli Lilly, Korea Pharma, Myoung Poom Medical, Otsuka, Sanofi-Aventis, and Yuhan. C Pollock has been a speaker or participated in advisory boards for Astra Zeneca, Boehringer Ingelheim, Eli Lilly, GlaxoSmithKline, Otsuka, Vifor-CSL. S Assad-Khalil, V Sánchez-Polo, and T Thanachayanont declare no conflict of interest in regard to this study.

REFERENCES

1. Kidney Disease: Improving Global Outcomes (KDIGO) 2022 Clinical Practice Guideline for Diabetes Management in Chronic Kidney Disease Kidney International (2022) 102 (Suppl 5S), S1–S127 S1.

For peer review only - http://bmjopen.bmj.com/site/about/guidelines.xhtml

2. Tuttle KR, Alicic RZ, Duru OK, et al. Clinical Characteristics of and Risk Factors for Chronic Kidney Disease Among Adults and Children: An Analysis of the CURE-CKD Registry. JAMA Netw Open. 2019 Dec 2;2(12):e1918169. doi: 10.1001/jamanetworkopen.2019.18169. PMID: 31860111; PMCID: PMC6991307.

3. Kadowaki T, Maegawa H, Watada H, et al. Interconnection between cardiovascular, renal and metabolic disorders: A narrative review with a focus on Japan. Diabetes Obes Metab. 2022 Dec;24(12):2283-2296. doi: 10.1111/dom.14829. Epub 2022 Aug 25. PMID: 35929483; PMCID: PMC9804928.

4. Thomas MC, Cooper ME, Zimmet P. Changing epidemiology of type 2 diabetes mellitus and associated chronic kidney disease. Nat Rev Nephrol. 2016 Feb;12(2):73-81. doi: 10.1038/nrneph.2015.173. Epub 2015 Nov 10. PMID: 26553517.

5. van de Wouw J, Broekhuizen M, Sorop O, et al. Chronic Kidney Disease as a Risk Factor for Heart Failure With Preserved Ejection Fraction: A Focus on Microcirculatory Factors and Therapeutic Targets. Front Physiol. 2019 Sep 4;10:1108. doi: 10.3389/fphys.2019.01108. PMID: 31551803; PMCID: PMC6737277.

6. da Silva LS, Cotta RMM, Moreira TR, et al. Hidden prevalence of chronic kidney disease in hypertensive patients: the strategic role of primary health care. Public Health. 2016 Nov;140:250-257. doi: 10.1016/j.puhe.2016.02.029. Epub 2016 Mar 29. PMID: 27036982.

7. Bikbov B, Purcell C, Levey A, et al. (2020). Global, regional, and national burden of chronic kidney disease, 1990–2017: a systematic analysis for the Global Burden of Disease Study 2017. The Lancet. 395. 709-733. 10.1016/S0140-6736(20)30045-3.

8. Shlipak MG, Tummalapalli SL, Boulware LE, et al. The case for early identification and intervention of chronic kidney disease: conclusions from a Kidney Disease: Improving Global Outcomes (KDIGO) Controversies Conference. Kidney Int. 2021 Jan;99(1):34-47. doi: 10.1016/j.kint.2020.10.012. Epub 2020 Oct 27. PMID: 33127436.

9. Couser WG, Remuzzi G, Mendis S, Tonelli M. The contribution of chronic kidney disease to the global burden of major noncommunicable diseases. Kidney Int. 2011 Dec;80(12):1258-70. doi: 10.1038/ki.2011.368. Epub 2011 Oct 12. PMID: 21993585.

10. Bastos MG, Kirsztajn GM. Chronic kidney disease: importance of early diagnosis, immediate referral and structured interdisciplinary approach to improve outcomes in patients not yet on dialysis. J Bras Nefrol. 2011 Mar;33(1):93-108. English, Portuguese. PMID: 21541469.

11. Kidney Disease: Improving Global Outcomes (KDIGO) CKD Work Group. KDIGO 2012 Clinical Practice Guideline for the Evaluation and Management of Chronic Kidney Disease. Kidney inter., Suppl. 2013; 3: 1–150.

12. Inker LA, Eneanya ND, Coresh J, et al. New Creatinine- and Cystatin C-Based Equations to Estimate GFR without Race. N Engl J Med. 2021 Nov 4;385(19):1737-1749. doi: 10.1056/NEJMoa2102953. Epub 2021 Sep 23. PMID: 34554658; PMCID: PMC8822996.

13. James G, Garcia Sanchez JJ, Carrero JJ, et al. Low Adherence to Kidney Disease: Improving Global Outcomes 2012 CKD Clinical Practice Guidelines Despite Clear Evidence of Utility. Kidney Int Rep. 2022 Jun 8;7(9):2059-2070. doi: 10.1016/j.ekir.2022.05.033. PMID: 36090504; PMCID: PMC9458998.

14. Kidney Disease: Improving Global Outcomes (KDIGO) Blood Pressure Work Group. Kidney Int. 2021 Mar;99(3S):S1-S87. Doi: 10.1016/j.kint.2020.11.003. PMID: 33637192.

15. Sperati CJ, Soman S, Agrawal V, et al; National Kidney Foundation Education Committee. Primary care physicians' perceptions of barriers and facilitators to management of chronic kidney disease: A mixed methods study. PLoS One. 2019 Aug 22;14(8):e0221325. doi: 10.1371/journal.pone.0221325. PMID: 31437198; PMCID: PMC6705804.

16. Lamprea-Montealegre JA, Joshi P, Shapiro AS, et al. Improving chronic kidney disease detection and treatment in the United States: the chronic kidney disease cascade of care (C3) study protocol. BMC Nephrol. 2022 Oct 12;23(1):331. doi: 10.1186/s12882-022-02943-z. PMID: 36224528; PMCID: PMC9554861.

17. Al-Ghamdi S, Abu-Alfa A, Alotaibi T, AlSaaidi A, AlSuwaida A, Arici M, Ecder T, El Koraie AF, Ghnaimat M, Hafez MH, Hassan M, Sqalli T. Chronic Kidney Disease Management in the Middle East and Africa: Concerns, Challenges, and Novel Approaches. Int J Nephrol Renovasc Dis. 2023 Apr 6;16:103-112. doi: 10.2147/IJNRD.S363133. PMID: 37051319; PMCID: PMC10084934.

18. Assaad-Khalil S, Elkafrawy N, Khaled M, Mogeib O, Badr H, Rashwan A, Youssef M, Eltamawy K, Mohamed S. A Phase II, Randomized, Double-Blind, Double-Dummy, Active-Controlled Clinical Trial to Investigate the Efficacy and Safety of NW Low-Glu® in Patients Newly Diagnosed with Type 2 Diabetes Mellitus. Evid Based Complement Alternat Med. 2022 Sep 27;2022:9176026. doi: 10.1155/2022/9176026. PMID: 36204125; PMCID: PMC9532066.

19. Bertoluci MC, Silva Júnior WS, Valente F, Araujo LR, Lyra R, de Castro JJ, Raposo JF, Miranda PAC, Boguszewski CL, Hohl A, Duarte R, Salles JEN, Silva-Nunes J, Dores J, Melo M, de Sá JR, Neves JS, Moreira RO, Malachias MVB, Lamounier RN, Malerbi DA, Calliari LE, Cardoso LM, Carvalho MR, Ferreira HJ, Nortadas R, Trujilho FR, Leitão CB, Simões JAR, Dos Reis MIN, Melo P, Marcelino M, Carvalho D. 2023 UPDATE: Luso-Brazilian evidence-based guideline for the management of antidiabetic therapy in type 2 diabetes. Diabetol Metab Syndr. 2023 Jul 19;15(1):160. doi: 10.1186/s13098-023-01121-x. PMID: 37468901; PMCID: PMC10354939.

20. Hsu YT, Lin JY, Lin CJ, Lee YJ, Chang WH. Association of Possible Sarcopenia or Sarcopenia with Body Composition, Nutritional Intakes, Serum Vitamin D Levels, and Physical Activity among Patients with Type 2 Diabetes Mellitus in Taiwan. Nutrients. 2023 Sep 7;15(18):3892. doi: 10.3390/nu15183892. PMID: 37764676; PMCID: PMC10535053.

21. Vasquez Jimenez E, Campos Núñez G, Lerma A, Lerma C, Meave González A, Pérez-Grovas H, López Gil S, Madero M. Neurocognitive Function with Conventional Hemodialysis Versus Post-Dilution Hemofiltration as Initial Treatment in ESKD patients. A randomized controlled trial. The DA-VINCI Study. Blood Purif. 2023 Oct 27. doi: 10.1159/000534823. Epub ahead of print. PMID: 37899042.

22. Glastras SJ, Pollock CA. Targeted identification of risk and treatment of diabetic kidney disease. Nat Rev Nephrol. 2023 Dec 1. doi: 10.1038/s41581-023-00796-9. Epub ahead of print. PMID: 38040863.

23. Barbagelata L, Masson W, Corral P, Lavalle-Cobo A, Nogueira JP, Rosa Diez G. Relationship between lipoprotein(a) levels, cardiovascular outcomes and death in patients with chronic kidney disease: a systematic review of prospective studies. J Nephrol. 2023 Jul;36(6):1549-1559. doi: 10.1007/s40620-023-01590-3. Epub 2023 Apr 8. PMID: 37029883.

24. Koh ES, Kim GH, Chung S. Intrarenal Mechanisms of Sodium-Glucose Cotransporter-2 Inhibitors on Tubuloglomerular Feedback and Natriuresis. Endocrinol Metab (Seoul). 2023 Aug;38(4):359-372. doi: 10.3803/EnM.2023.1764. Epub 2023 Jul 24. PMID: 37482684; PMCID: PMC10475968.

25. Thanachayanont T, Chanpitakkul M, Saetie A, Lekagul S, Tungsanga K. Correlation of the Dietary Protein Intake between Those Estimated from a Short Protein Food-Recall Questionnaire and from 24-Hour Urinary Urea-Nitrogen Excretion in Stages 3-4 Chronic

Kidney Disease Patients. Int J Nephrol. 2023 Nov 22;2023:9713045. doi: 10.1155/2023/9713045. PMID: 38033373; PMCID: PMC10686714.

26. Burton JO, Coats AJS, Kovesdy CP, et al. An international Delphi consensus regarding best practice recommendations for hyperkalaemia across the cardiorenal spectrum. Eur J Heart Fail. 2022 Sep;24(9):1467-1477. doi: 10.1002/ejhf.2612. Epub 2022 Aug 4. Erratum in: Eur J Heart Fail. 2023 Mar;25(3):444. PMID: 35791065; PMCID: PMC9804940.

27. Diamond IR, Grant RC, Feldman BM, et al. Defining consensus: a systematic review recommends methodologic criteria for reporting of Delphi studies. J Clin Epidemiol. 2014 Apr;67(4):401-9. doi: 10.1016/j.jclinepi.2013.12.002. PMID: 24581294.

28. Li PK, Garcia-Garcia G, Lui SF, et al. Kidney health for everyone everywhere - from prevention to detection and equitable access to care. Clin Nephrol. 2020 Mar;93(3):111-122. doi: 10.5414/CNWKDEditorial. PMID: 32017699.

29. Komenda P, Ferguson TW, Macdonald K, et al. Cost-effectiveness of primary screening for CKD: a systematic review. Am J Kidney Dis. 2014 May;63(5):789-97. doi: 10.1053/j.ajkd.2013.12.012. Epub 2014 Feb 12. PMID: 24529536.

30. Aseneh, J.B., Kemah, BL.A., Mabouna, S. et al. Chronic kidney disease in Cameroon: a scoping review. BMC Nephrol 21, 409 (2020). https://doi.org/10.1186/s12882-020-02072-5

31. Ulasi II, Awobusuyi O, Nayak S, Ramachandran R, Musso CG, Depine SA, Aroca-Martinez G, Solarin AU, Onuigbo M, Luyckx VA, Ijoma CK. Chronic Kidney Disease Burden in Low-Resource Settings: Regional Perspectives. Semin Nephrol. 2022 Sep;42(5):151336. doi: 10.1016/j.semnephrol.2023.151336. Epub 2023 Apr 12. PMID: 37058859.

32. ISN-Global Kidney Health Atlas. International Society of Nephrology. 3rd Edition. 2023.

33. Sammut-Powell C, Sisk R, Budd J, Patel N, Edge M, Cameron R. Development of minimal resource pre-screening tools for chronic kidney disease in people with type 2 diabetes. Future Healthc J. 2022 Nov;9(3):305-309. doi: 10.7861/fhj.2022-0020. PMID: 36561833; PMCID: PMC9761456.

34. Kellum JA, Romagnani P, Ashuntantang G, Ronco C, Zarbock A, Anders HJ. Acute kidney injury. Nat Rev Dis Primers. 2021 Jul 15;7(1):52. doi: 10.1038/s41572-021-00284-z. PMID: 34267223.

35. Schytz PA, Blanche P, Nissen AB, et al. Acute kidney injury and risk of cardiovascular outcomes: A nationwide cohort study. Nefrologia (Engl Ed). 2021 Sep 3:S0211-6995(21)00140-5. English, Spanish. doi: 10.1016/j.nefro.2021.06.007. Epub ahead of print. PMID: 34489123.

36. Thomas M, Magwire M, Gosch K, et al. Cardiometabolic Center of Excellence: A Novel Care Delivery Model for Secondary Prevention of Cardiovascular Disease in Type 2 Diabetes. Circ Cardiovasc Qual Outcomes. 2021 Oct;14(10):e007682. doi: 10.1161/CIRCOUTCOMES.120.007682. Epub 2021 Sep 30. PMID: 34587753; PMCID: PMC8530947.

37. Weber C, Beaulieu M, Djurdjev O, et al. Towards rational approaches of health care utilization in complex patients: an exploratory randomized trial comparing a novel combined clinic to multiple specialty clinics in patients with renal disease-cardiovascular disease-diabetes. Nephrol Dial Transplant. 2012 Oct;27 Suppl 3:iii104-10. doi: 10.1093/ndt/gfr292. Epub 2011 Jun 30. PMID: 21719715.

38. Spaak J. Novel combined management approaches to patients with diabetes, chronic kidney disease and cardiovascular disease. J R Coll Physicians Edinb. 2017 Mar;47(1):83-87. doi: 10.4997/JRCPE.2017.118. PMID: 28569290.

39. Nichols GA, Ustyugova A, Déruaz-Luyet A, O'Keeffe-Rosetti M, Brodovicz KG. Health Care Costs by Type of Expenditure across eGFR Stages among Patients with and without Diabetes, Cardiovascular Disease, and Heart Failure. J Am Soc Nephrol. 2020 Jul;31(7):1594-1601. doi: 10.1681/ASN.2019121308. Epub 2020 Jun 2. PMID: 32487562; PMCID: PMC7350988.

40. Yang A, Shi M, Lau ESH, et al. Clinical outcomes following discontinuation of renin-angiotensin-system inhibitors in patients with type 2 diabetes and advanced chronic kidney disease: A prospective cohort study. EClinicalMedicine. 2022 Nov 24;55:101751. doi: 10.1016/j.eclinm.2022.101751. PMID: 36457651; PMCID: PMC9706514.

41. Fu EL, Evans M, Clase CM, et al. Stopping Renin-Angiotensin System Inhibitors in Patients with Advanced CKD and Risk of Adverse Outcomes: A Nationwide Study. J Am Soc Nephrol. 2021 Feb;32(2):424-435. doi: 10.1681/ASN.2020050682. Epub 2020 Dec 28. PMID: 33372009; PMCID: PMC8054897.

42. Qiao Y, Shin J, Chen TK, et al. Association Between Renin-Angiotensin System Blockade Discontinuation and All-Cause Mortality Among Persons With Low Estimated Glomerular Filtration Rate. JAMA Intern Med. 2020;180(5):718–726. doi:10.1001/jamainternmed.2020.0193.

43. Clark AL, Kalra PR, Petrie MC, Mark PB, Tomlinson LA, Tomson CR. Change in renal function associated with drug treatment in heart failure: national guidance. Heart. 2019 Jun;105(12):904-910. doi: 10.1136/heartjnl-2018-314158. PMID: 31118203; PMCID: PMC6582720.

44. Morales E, Cravedi P, Manrique J. Management of Chronic Hyperkalemia in Patients With Chronic Kidney Disease: An Old Problem With News Options. Front Med (Lausanne). 2021 Jun 4;8:653634. doi: 10.3389/fmed.2021.653634. PMID: 34150795; PMCID: PMC8213200.

45. Kim GH. Pharmacologic Treatment of Chronic Hyperkalemia in Patients with Chronic Kidney Disease. Electrolyte Blood Press. 2019 Jun;17(1):1-6. doi: 10.5049/EBP.2019.17.1.1. Epub 2019 Jun 30. PMID: 31338108; PMCID: PMC6629599.

46. Clase CM, Carrero JJ, Ellison DH, et al. Potassium homeostasis and management of dyskalemia in kidney diseases: conclusions from a Kidney Disease: Improving Global Outcomes (KDIGO) Controversies Conference. Kidney Int. 2020 Jan;97(1):42-61. doi: 10.1016/j.kint.2019.09.018. Epub 2019 Oct 10. PMID: 31706619.

47. Wang AY. Optimally managing hyperkalemia in patients with cardiorenal syndrome. Nephrol Dial Transplant. 2019 Dec 1;34(Suppl 3):iii36-iii44. Doi: 10.1093/ndt/gfz225. PMID: 31800079.

48. Silva-Cardoso J, Brito D, Frazão JM, et al. Management of RAASi-associated hyperkalemia in patients with cardiovascular disease. Heart Fail Rev. 2021 Jul;26(4):891-896. Doi: 10.1007/s10741-020-10069-3. Epub 2021 Feb 18. PMID: 33599908; PMCID: PMC8149346.

49. Epstein M, Reaven NL, Funk SE, McGaughey KJ, Oestreicher N, Knispel J. Evaluation of the treatment gap between clinical guidelines and the utilization of renin-angiotensin-aldosterone system inhibitors. Am J Manag Care. 2015 Sep;21(11 Suppl):S212-20. PMID: 26619183.

50. Trevisan M, de Deco P, Xu H, et al. Incidence, predictors, and clinical management of hyperkalaemia in new users of mineralocorticoid receptor antagonists. Eur J Heart Fail. 2018 Aug;20(8):1217-1226. Doi: 10.1002/ejhf.1199. Epub 2018 Apr 18. Erratum in: Eur J Heart Fail. 2019 Apr;21(4):540. PMID: 29667759; PMCID: PMC6607478.

51. Fraser SD, Roderick PJ, May CR, et al. The burden of comorbidity in people with chronic kidney disease stage 3: a cohort study. BMC Nephrol. 2015 Dec 1;16:193. doi: 10.1186/s12882-015-0189-z. Erratum in: BMC Nephrol. 2020 Dec 21;21(1):543. PMID: 26620131; PMCID: PMC4666158.

52. Lee WC, Lee YT, Li LC, et al. The Number of Comorbidities Predicts Renal Outcomes in Patients with Stage 3‑5 Chronic Kidney Disease. J Clin Med. 2018 Nov 28;7(12):493. doi: 10.3390/jcm7120493. PMID: 30486496; PMCID: PMC6306906.

For peer review only

Appendix: Supplementary Data

Table S1: Proportion of responses for each agreement level by statement

<<Table S1>>

Figure S1: Proportion of responses for each agreement level by statement

<<Figure S1>>

Table S2: Anonymized Round 2 Response Data

<<Table S2>>

For peer review only

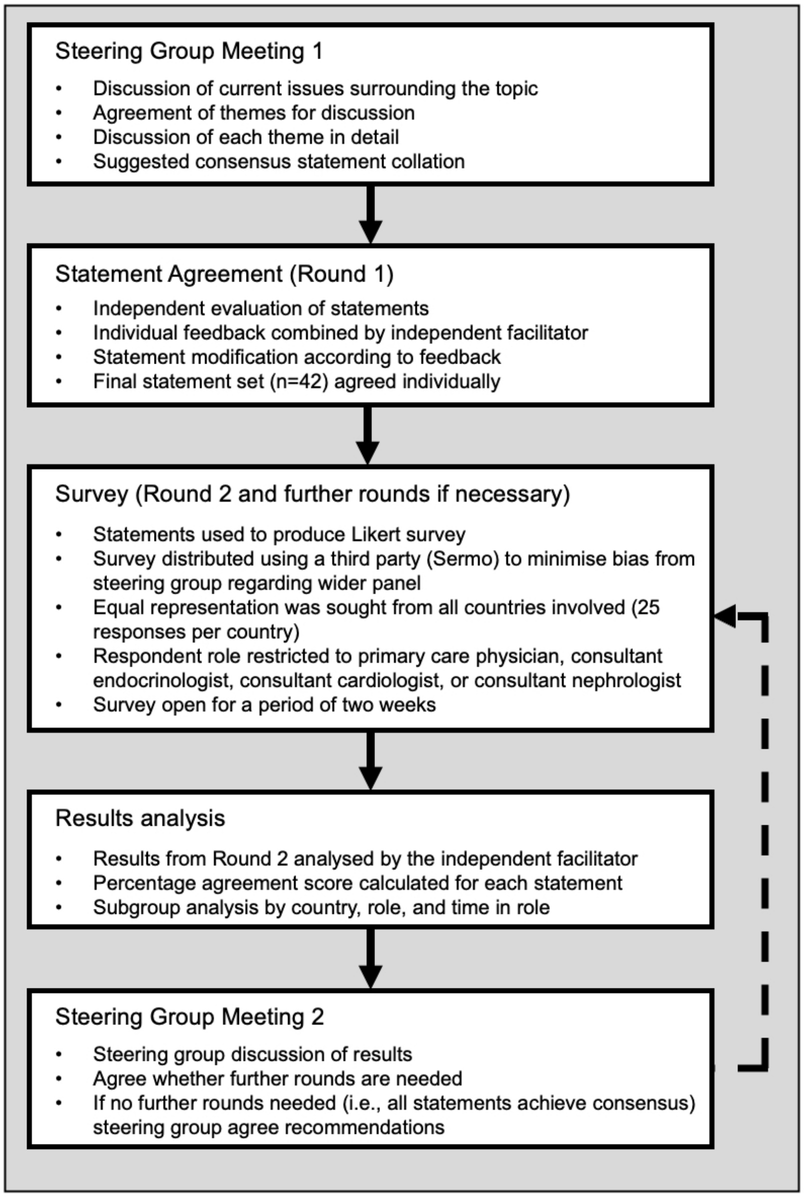

Figure 1: Study flow diagram

282x418mm (72 x 72 DPI)

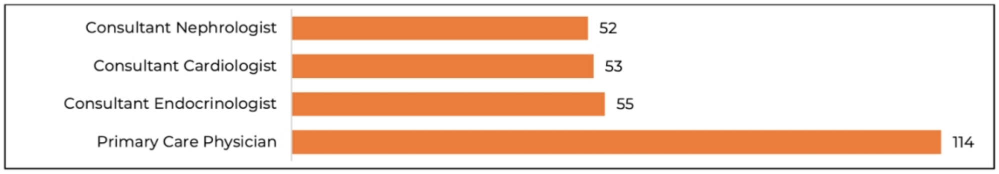

Figure 2: Respondents by role

525x91mm (72 x 72 DPI)

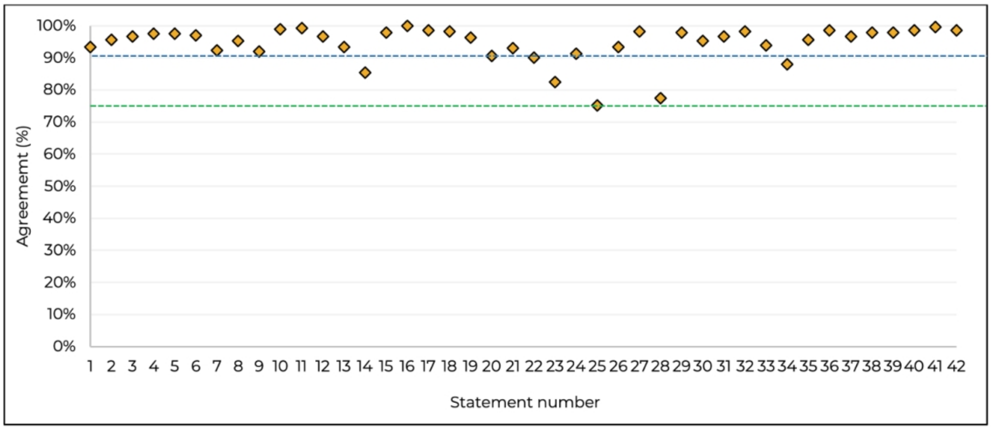

Figure 3: Overall consensus agreement levels by statementa

521x225mm (72 x 72 DPI)

No:	Statement:	Strongly agree	Tend to agree	Tend to disagree	Strongly disagree	Overall
**Earlier identification & screening of CKD**						
**1.**	Late diagnosis is a lost opportunity for early management of CKD which can slow disease progression	77%	16%	3%	4%	**93%**
**2.**	National screening and diagnostic programmes for CKD are essential to identifying patients at earlier stages of CKD	69%	27%	3%	2%	**96%**
**3.**	A simple and practical definition of 'high-risk' should be established to support cost-effective screening	65%	32%	2%	1%	**97%**
**4.**	Early screening for CKD in high-risk groups is cost-effective for the health system (where resources are in place to support intervention)	71%	27%	1%	1%	**97%**
**5.**	Primary care physicians and specialists (cardiologists, endocrinologists, nephrologists, diabetologists, etc.) should routinely screen for CKD in patients with risk factors	76%	21%	1%	1%	**97%**
**6.**	Patients with risk factors (e.g., elderly, diabetes, hypertension, dyslipidaemia, obesity, family history of CKD) should be screened at least annually for CKD	81%	16%	2%	1%	**97%**
**7.**	Patients with CKD should be screened for heart failure (including those with preserved ejection fraction (HFpEF))	46%	46%	6%	2%	**92%**
**8.**	GFR estimated by the CKD-EPI Creatinine Equation (2021) and albuminuria (using albumin-to-creatinine ratio) should be the screening method of choice for CKD	55%	41%	4%	1%	**95%**
**9.**	Confirmatory testing of abnormalities of kidney function or structure at 3 months is required to establish a CKD diagnosis	48%	44%	7%	1%	**92%**
**Risk factors for CKD in cardio-renal-metabolic patients**						
**10.**	A holistic view of cardio-renal-metabolic disease states is critical to provide integrated patient-centred care to individuals with CKD	74%	25%	0%	1%	**99%**
**11.**	CKD progressively increases the risk of CVD, including heart failure and overall mortality	75%	24%	0%	1%	**99%**
**12.**	Frequency of hyperkalaemia increases as CKD progresses	62%	34%	3%	1%	**97%**
**13.**	AKI is a risk factor for CKD and chronic heart failure	57%	37%	5%	1%	**93%**
**14.**	Infection, including COVID-19, can cause AKI and is therefore a risk factor for CKD	42%	43%	14%	1%	**85%**
**15.**	Nephrotoxic medications should be modified, and risk/benefit profiles should be assessed in patients with, or at risk of CKD	72%	26%	1%	1%	**98%**
**Holistic management of CKD in cardio-renal-metabolic patients**						
**16.**	Lifestyle factors such as diet, smoking, exercise must be optimised for CKD patients	83%	17%	0%	0%	**100%**
**17.**	Disease modifying therapies for CKD (e.g., SGLT2i, RAASi, MRA) should be used where indicated to manage progression of CKD	69%	29%	1%	0%	**99%**
**18.**	SGLT2i and RAASi have a complementary cardio-renal protective action	61%	37%	1%	0%	**98%**
**19.**	Early use of SGLT2i could prevent the development and progression of CKD patients with type 2 diabetes and heart failure	62%	34%	3%	0%	**96%**
**20.**	Early use of SGLT2i can slow progression of CKD in patients without diabetes	45%	45%	9%	1%	**91%**
**21.**	A small decrease of eGFR (~10%) may be expected on initiation of CKD disease-modifying therapies	39%	54%	7%	0%	**93%**
**22.**	De-escalation or discontinuation of RAASi therapy is associated with worse cardiovascular and renal outcomes in the CKD patient	33%	57%	9%	1%	**90%**
**23.**	Disease modifying therapies for CKD should only be stopped as a last resort	33%	50%	15%	3%	**82%**
**24.**	Novel K binders (i.e., patiromer and sodium zirconium cyclosilicate) are an option to manage hyperkalaemia and prevent de-escalation or downtitration of RAASi	27%	65%	8%	0%	**91%**
**25.**	Action to manage hyperkalaemia in the CKD patient should be taken when serum potassium level reaches 5.0mmol/L	22%	54%	20%	5%	**75%**
**26.**	Action to manage hyperkalaemia in the CKD patient should be taken when serum potassium level reaches 5.5 mmol/L	53%	40%	5%	1%	**93%**

**27.**	Treatment targets such as blood pressure, blood glucose, lipid levels should be maintained for both kidney and cardiac outcomes in CKD patients	81%	17%	1%	0%	**98%**
**Guidelines**						
**28.**	Implementation of evidence-based CKD guidelines is suboptimal and should be improved	29%	48%	20%	2%	**77%**
**29.**	Cardiology, nephrology, and endocrinology guidelines for CKD should be aligned	62%	36%	2%	0%	**98%**
**30.**	Primary care physicians have an important role in implementing guidelines	66%	30%	4%	1%	**95%**
**31.**	Guidelines should be practical with an executive summary / checklist to assist implementation by non-specialist HCPs	65%	31%	3%	0%	**97%**
**32.**	Guidelines should include clear criteria for when and how to refer to other specialists/MDT	69%	30%	2%	0%	**98%**
**33.**	Guidelines should reflect differences in population phenotypes and be locally adapted where needed	58%	35%	6%	0%	**94%**
**34.**	Patient perspective is crucial in developing guidelines	42%	46%	11%	1%	**88%**
**35.**	Investment is needed to shift the approach to primary prevention rather than end stage kidney disease management	69%	26%	3%	1%	**96%**
**Cross-specialty alignment (Cardiology, Nephrology, Endocrinology, Primary Care & Policy Makers)**						
**36.**	Patients with CKD should be managed by a multi-disciplinary team (MDT) where possible with an agreed management plan	67%	31%	1%	0%	**99%**
**37.**	The CKD multi-disciplinary team (MDT) should include the primary care physician to improve early intervention and decision making	65%	32%	3%	0%	**97%**
**38.**	The care pathway for CKD should be designed to minimise the impact of organisational barriers	58%	39%	2%	0%	**98%**
**39.**	Clinicians, professional associations, academic institutions, and patient representative organisations need to engage with policy makers to ensure appropriate plans and funding are in place to deliver optimal CKD care	67%	31%	2%	0%	**98%**
**Education of clinicians and patients**						
**40.**	Up-to-date healthcare professional (HCP) education on the management of patients with CKD and associated guidelines is needed	72%	27%	1%	0%	**99%**
**41.**	Structured education of primary care physicians on screening and early detection improves patient outcomes	76%	24%	0%	0%	**100%**
**42.**	Therapeutic patient education is key for them to understand the consequences of CKD and how to manage it through lifestyle modification and appropriate use of therapies	73%	26%	1%	0%	**99%**

For peer review only

ID	Role	Country	Experience	No. CKD patients every 3 months	Statement Number																																								
					1	2	3	4	5	6	7	8	9	10	11	12	13	14	15	16	17	18	19	20	21	22	23	24	25	26	27	28	29	30	31	32	33	34	35	36	37	38	39	40	41	42
1	Primary Care Physician	AUS	More than 10 years	10-19 patients	1	1	1	1	1	1	2	1	2	1	1	2	1	2	1	1	1	2	2	2	2	2	2	2	2	3	1	3	1	2	1	1	2	2	2	2	1	1	2	2	2	2
2	Primary Care Physician	AUS	More than 10 years	20-29 patients	1	1	1	1	1	1	1	1	1	1	1	2	1	1	1	1	1	1	1	2	2	2	2	2	2	1	1	1	1	1	1	1	1	1	1	1	1	1	1	1	1	1
3	Consultant Endocrinologist	AUS	More than 10 years	100+ patients	1	1	1	1	1	1	1	1	2	1	1	1	1	1	1	1	1	2	2	2	1	2	1	2	2	1	1	1	1	1	1	1	1	1	1	1	1	1	1	1	2	1
4	Primary Care Physician	AUS	More than 10 years	80-89 patients	1	2	2	1	2	1	2	2	2	2	1	2	2	1	1	2	2	2	2	2	2	3	2	2	2	3	1	3	2	1	2	1	1	2	1	1	1	1	1	1	1	1
5	Primary Care Physician	AUS	6 - 10 years	40-49 patients	2	2	2	2	2	1	2	3	2	2	1	1	3	3	2	1	2	3	3	3	2	1	2	3	1	1	1	3	2	1	1	1	1	1	1	1	1	1	1	2	1	2
6	Primary Care Physician	AUS	More than 10 years	80-89 patients	1	2	1	1	1	1	2	1	2	2	1	2	1	1	1	1	1	2	1	1	2	2	3	3	3	2	1	3	3	1	2	1	2	2	1	1	1	1	1	1	2	1
7	Primary Care Physician	AUS	More than 10 years	30-39 patients	1	2	1	2	2	3	3	3	2	2	2	1	3	2	3	2	2	2	3	1	1	4	3	2	3	1	1	1	2	2	1	3	2	3	4	1	1	2	1	2	2	2
8	Primary Care Physician	AUS	6 - 10 years	90-99 patients	1	1	1	1	1	1	1	1	2	1	1	2	2	2	1	1	1	2	1	2	2	2	2	2	2	1	1	2	1	1	1	1	2	2	1	1	1	1	1	1	1	1
9	Primary Care Physician	AUS	6 - 10 years	50-59 patients	1	2	2	2	1	1	1	2	2	1	1	1	1	1	1	1	2	1	1	2	2	3	1	3	2	3	1	2	1	1	1	1	1	2	1	1	1	1	1	2	1	1
10	Primary Care Physician	AUS	More than 10 years	40-49 patients	2	2	1	2	1	1	3	1	1	1	1	2	2	2	1	1	1	1	1	1	2	1	1	2	4	2	1	1	1	1	2	1	1	1	2	1	2	2	2	1	1	2
11	Primary Care Physician	AUS	6 - 10 years	40-49 patients	1	2	1	2	2	1	1	2	2	2	2	1	2	3	1	1	1	1	1	1	2	2	2	3	2	1	1	2	2	1	1	1	1	1	2	2	1	1	2	1	2	2
12	Consultant Cardiologist	AUS	6 - 10 years	50-59 patients	1	2	2	2	2	2	1	2	2	1	1	1	2	2	2	2	2	2	2	2	3	2	2	2	3	2	2	2	2	2	2	3	2	4	2	2	3	2	2	2	2	2
13	Consultant Cardiologist	AUS	6 - 10 years	50-59 patients	2	2	1	1	2	2	2	1	1	2	1	3	2	3	2	1	1	2	2	2	1	2	2	1	3	2	2	2	2	1	1	2	2	2	1	2	1	2	1	2	2	1
14	Consultant Cardiologist	AUS	More than 10 years	70-79 patients	2	2	1	2	1	2	1	2	2	1	2	1	1	1	2	1	1	2	1	2	2	2	2	2	1	2	1	2	2	2	2	2	2	2	1	2	1	2	2	2	2	2
15	Consultant Cardiologist	AUS	More than 10 years	100+ patients	1	1	1	1	1	1	1	1	1	1	1	2	1	2	1	2	1	2	1	2	2	1	2	2	2	2	1	2	1	2	1	2	2	3	1	2	2	1	2	2	2	2
16	Consultant Cardiologist	AUS	More than 10 years	100+ patients	1	1	1	1	1	1	1	1	1	1	1	1	1	1	1	1	1	1	1	1	1	1	1	2	1	2	1	2	1	1	1	1	1	1	2	1	1	1	1	1	1	1
17	Primary Care Physician	MEX	6 - 10 years	60-69 patients	1	1	1	1	1	1	2	2	1	1	1	1	2	2	1	1	1	1	1	2	2	2	2	1	2	2	1	2	1	1	1	2	1	2	1	1	1	1	1	1	1	1
18	Consultant Cardiologist	MEX	More than 10 years	30-39 patients	1	1	1	1	1	1	2	1	1	1	1	1	1	2	1	1	1	1	1	1	1	1	1	1	2	1	1	1	1	1	1	1	1	2	1	1	1	1	1	1	1	1
19	Primary Care Physician	TWN	More than 10 years	10-19 patients	1	1	1	1	1	1	1	1	1	1	1	1	2	2	1	1	1	1	1	1	1	1	1	1	2	1	1	1	1	1	1	1	1	1	1	1	1	1	1	1	1	1
20	Consultant Endocrinologist	MEX	More than 10 years	60-69 patients	2	1	2	1	1	1	2	1	2	1	2	3	3	2	2	1	1	2	2	1	2	3	2	2	3	2	1	1	1	2	2	1	1	3	2	2	1	2	2	2	1	2
21	Primary Care Physician	MEX	6 - 10 years	100+ patients	1	1	1	1	1	1	1	2	1	1	1	1	2	1	1	1	1	1	1	3	1	1	1	2	2	2	1	1	1	1	1	1	1	1	1	1	1	1	1	1	1	1
22	Primary Care Physician	TUR	6 - 10 years	60-69 patients	1	1	1	1	1	1	1	2	1	1	1	1	1	1	1	1	2	2	2	2	2	2	2	1	1	1	1	3	2	1	1	1	1	1	1	1	1	1	1	1	1	1
23	Primary Care Physician	TUR	Less than 5 Years	10-19 patients	1	1	1	1	2	1	2	2	2	1	1	1	1	2	1	1	1	1	1	2	2	2	1	2	3	2	1	3	1	3	1	1	1	1	1	1	1	1	1	1	1	1
24	Consultant Nephrologist	MEX	6 - 10 years	30-39 patients	1	1	1	1	2	1	1	1	1	1	1	1	1	1	1	1	1	1	1	1	1	2	3	2	2	2	1	3	2	2	2	1	2	2	2	2	2	2	2	1	1	1
25	Consultant Endocrinologist	BRA	More than 10 years	100+ patients	1	1	1	1	1	1	1	1	1	1	1	2	1	1	1	1	1	1	1	1	1	1	1	2	2	2	1	1	1	1	1	1	1	1	1	1	1	1	1	1	1	1
26	Primary Care Physician	TUR	Less than 5 Years	10-19 patients	2	1	2	2	2	1	1	1	2	2	2	2	2	2	2	2	2	2	2	2	2	2	2	2	2	2	2	2	2	2	2	2	2	2	2	2	2	2	2	2	2	2
27	Consultant Cardiologist	MEX	Less than 5 Years	50-59 patients	1	1	1	1	1	1	1	2	1	1	1	2	2	2	2	1	1	1	1	1	1	1	1	2	2	1	1	1	1	1	2	2	2	3	1	1	1	1	1	1	1	2
28	Consultant Nephrologist	TUR	Less than 5 Years	30-39 patients	2	1	2	4	3	2	2	1	1	1	1	1	1	2	1	2	1	1	2	1	3	2	2	2	1	1	1	2	1	1	1	1	1	2	1	1	2	1	1	1	1	1
29	Primary Care Physician	TUR	Less than 5 Years	10-19 patients	1	1	1	1	2	2	2	2	2	1	1	1	1	2	1	1	1	2	2	2	2	2	1	2	2	1	1	2	1	1	1	1	1	1	1	1	1	1	1	2	2	2
30	Primary Care Physician	TUR	Less than 5 Years	10-19 patients	4	2	4	1	3	1	3	4	4	1	1	4	4	4	2	1	4	4	4	4	1	4	4	4	4	2	4	4	4	4	4	1	3	4	4	3	4	4	1	4	4	3
31	Primary Care Physician	BRA	6 - 10 years	30-39 patients	2	1	2	1	1	1	3	1	2	1	2	1	2	3	1	1	2	1	2	2	3	2	2	3	1	3	1	2	2	1	1	1	1	2	1	1	1	1	1	1	1	1
32	Consultant Nephrologist	MEX	6 - 10 years	80-89 patients	1	1	2	1	1	1	1	1	1	1	1	1	1	1	1	1	2	1	1	2	1	1	2	2	3	1	1	2	1	2	1	1	1	1	1	1	2	2	2	1	1	1
33	Primary Care Physician	BRA	More than 10 years	100+ patients	1	3	2	2	1	1	1	1	2	1	2	1	2	2	2	1	2	2	2	2	2	2	3	2	3	2	1	2	2	1	1	2	3	2	2	1	1	1	1	2	1	1
34	Primary Care Physician	TUR	6 - 10 years	10-19 patients	1	1	1	1	1	1	1	1	1	1	1	1	1	1	1	1	1	1	1	1	1	1	1	1	4	1	1	2	2	3	2	1	1	1	1	1	1	1	1	1	1	1
35	Consultant Endocrinologist	BRA	More than 10 years	50-59 patients	1	1	1	1	1	1	1	1	1	1	1	1	1	1	1	1	1	1	1	2	1	1	2	2	2	1	1	1	1	2	1	1	1	1	1	1	2	1	2	1	1	1
36	Primary Care Physician	TUR	Less than 5 Years	10-19 patients	1	1	1	1	2	1	1	1	1	1	1	1	1	1	1	1	1	1	1	1	1	1	1	1	1	1	1	1	1	1	1	1	1	1	1	1	1	1	1	1	1	1
37	Primary Care Physician	BRA	6 - 10 years	80-89 patients	1	1	1	1	2	1	2	1	2	1	2	2	1	1	1	1	1	1	3	3	2	1	1	1	1	1	1	1	2	1	1	1	1	3	1	1	2	1	2	1	1	1
38	Consultant Endocrinologist	TUR	6 - 10 years	20-29 patients	1	1	2	2	2	1	2	2	1	2	2	1	1	1	1	1	2	2	2	2	2	2	2	2	1	1	1	2	2	2	2	2	2	2	2	2	2	2	2	2	2	2
39	Primary Care Physician	BRA	More than 10 years	100+ patients	1	1	1	1	1	1	1	1	2	1	1	1	1	1	1	1	1	1	1	1	1	1	1	1	1	1	1	1	1	1	1	1	1	1	1	1	1	1	1	1	1	1
40	Primary Care Physician	TUR	Less than 5 Years	100+ patients	2	2	1	1	1	1	1	2	3	1	2	1	1	1	1	1	2	1	2	2	1	2	3	2	2	1	1	2	1	1	1	1	1	2	1	1	1	1	1	1	1	1
41	Primary Care Physician	TUR	Less than 5 Years	10-19 patients	3	4	2	2	1	1	1	2	2	3	1	1	1	1	1	1	1	2	1	1	1	1	1	1	1	1	2	1	1	1	1	1	1	1	1	1	2	2	3	1	1	2
42	Primary Care Physician	BRA	6 - 10 years	70-79 patients	2	1	2	2	2	1	3	2	3	1	1	2	2	1	1	1	1	1	2	1	2	2	3	3	1	2	1	2	2	1	1	3	1	3	1	1	1	2	2	2	1	1
43	Consultant Cardiologist	TUR	6 - 10 years	20-29 patients	1	2	1	2	1	1	2	2	2	1	1	2	1	1	2	1	1	1	1	1	3	1	4	1	2	3	1	2	2	1	1	1	1	1	2	1	1	1	2	2	2	1
44	Consultant Cardiologist	TUR	More than 10 years	60-69 patients	1	2	1	1	1	1	1	2	1	1	1	2	2	2	1	1	1	1	1	2	2	2	2	2	2	1	1	2	1	3	2	1	1	2	1	1	2	2	1	1	1	1
45	Consultant Nephrologist	MEX	6 - 10 years	40-49 patients	2	1	1	1	1	1	2	1	2	2	2	1	3	3	2	1	1	1	1	3	2	2	2	2	3	2	1	2	1	1	1	1	2	3	1	2	2	2	1	1	1	1
46	Consultant Endocrinologist	BRA	More than 10 years	10-19 patients	1	2	2	1	1	1	1	1	1	1	1	2	2	1	1	2	1	1	2	2	2	1	2	2	3	1	1	2	1	2	1	1	2	2	2	1	1	2	2	1	1	1
47	Primary Care Physician	BRA	6 - 10 years	40-49 patients	1	1	2	2	2	1	2	3	2	2	1	2	1	2	2	1	1	1	2	2	2	2	1	3	2	2	1	1	1	1	1	1	1	1	1	1	1	2	2	1	1	1
48	Consultant Nephrologist	BRA	More than 10 years	100+ patients	1	2	2	2	2	2	2	2	2	2	2	2	2	2	1	2	2	2	1	1	2	2	2	2	2	2	2	2	2	2	2	2	2	2	2	2	2	2	2	1	2	2
49	Primary Care Physician	BRA	More than 10 years	80-89 patients	1	2	1	3	1	1	1	2	2	2	1	1	3	2	1	1	1	1	1	1	2	1	3	3	4	3	2	2	1	2	1	2	4	1	1	1	2	2	2	3	1	2
50	Primary Care Physician	MEX	More than 10 years	40-49 patients	1	2	1	1	1	1	1	1	2	2	1	1	1	1	1	1	1	1	1	1	2	2	2	2	2	1	1	1	1	1	1	2	1	2	1	2	2	1	2	1	1	2
51	Consultant Endocrinologist	MEX	More than 10 years	60-69 patients	1	1	2	1	1	1	2	2	1	1	1	1	2	2	1	1	1	1	2	1	1	2	2	2	2	1	1	1	1	2	2	1	2	1	1	1	2	1	1	1	2	1
52	Primary Care Physician	MEX	More than 10 years	60-69 patients	2	2	1	2	1	1	1	2	2	1	1	2	1	1	1	1	2	1	1	1	1	2	2	1	2	1	1	2	1	1	1	1	1	1	1	1	1	1	1	1	1	1
53	Consultant Cardiologist	BRA	6 - 10 years	40-49 patients	2	2	2	2	2	2	2	2	2	2	2	2	2	2	2	2	2	2	2	2	2	2	2	2	2	2	2	3	2	3	2	2	2	2	2	3	3	3	2	2	2	2
54	Primary Care Physician	BRA	Less than 5 Years	100+ patients	1	1	1	1	1	1	1	1	1	1	1	2	2	2	1	1	1	1	1	1	2	2	1	2	4	2	1	2	2	2	1	1	1	1	1	1	1	1	1	1	1	1
55	Primary Care Physician	BRA	More than 10 years	40-49 patients	1	1	1	1	1	1	1	1	1	1	1	1	1	1	1	1	1	1	1	1	1	2	2	2	1	2	2	2	1	1	1	2	1	2	1	2	2	1	1	1	2	2
56	Consultant Nephrologist	MEX	6 - 10 years	100+ patients	1	2	1	1	1	1	2	1	1	2	1	1	1	1	1	2	1	1	1	1	2	2	2	1	3	2	1	2	1	3	2	1	1	1	1	2	1	1	1	1	1	2
57	Consultant Endocrinologist	BRA	More than 10 years	90-99 patients	1	1	1	1	1	1	1	2	2	1	1	2	1	1	1	1	1	1	1	2	2	2	2	1	2	1	2	2	1	1	1	1	2	2	2	1	2	2	2	2	1	1
58	Primary Care Physician	MEX	More than 10 years	100+ patients	2	2	1	1	1	1	1	2	2	1	1	1	2	1	2	2	1	1	1	2	2	2	3	2	2	2	1	2	1	2	1	1	1	1	1	2	2	3	2	2	1	1
59	Consultant Cardiologist	TUR	6 - 10 years	90-99 patients	2	1	1	1	1	1	1	2	2	1	1	2	1	2	2	1	3	2	1	3	1	2	1	2	2	1	1	2	2	4	2	2	1	2	2	2	3	2	2	1	2	3
60	Consultant Nephrologist	MEX	More than 10 years	100+ patients	1	1	1	1	1	1	1	1	1	1	1	1	1	1	1	1	1	1	1	1	1	1	1	1	1	1	1	2	1	1	1	1	1	1	1	1	1	1	1	1	1	1
61	Primary Care Physician	MEX	6 - 10 years	100+ patients	2	2	3	2	3	3	2	3	3	2	1	2	2	3	3	2	2	2	3	3	2	2	3	3	2	2	2	2	2	2	2	2	2	2	2	2	2	2	2	2	2	2
62	Consultant Endocrinologist	MEX	6 - 10 years	100+ patients	1	1	1	1	1	1	1	1	1	2	1	1	1	1	1	1	1	1	1	1	1	1	3	2	2	1	1	2	1	1	1	1	1	2	1	1	1	1	1	1	1	1
63	Consultant Endocrinologist	MEX	More than 10 years	20-29 patients	1	1	1	1	1	1	1	1	1	1	1	1	1	1	1	1	1	1	1	1	2	2	1	2	1	2	1	3	1	2	1	1	1	1	1	1	1	1	1	1	1	1
64	Consultant Endocrinologist	MEX	More than 10 years	90-99 patients	1	2	2	1	1	2	3	3	3	1	2	2	2	3	3	1	3	2	2	2	2	1	2	2	3	2	3	2	2	1	3	2	2	3	3	1	2	2	3	1	2	3
65	Consultant Cardiologist	TUR	6 - 10 years	100+ patients	2	2	2	1	2	2	2	2	2	2	1	1	1	2	1	1	1	1	1	1	2	2	2	2	3	2	2	3	3	2	2	3	3	2	4	2	2	2	2	3	2	2
66	Consultant Endocrinologist	ARG	More than 10 years	50-59 patients	1	1	2	1	1	1	2	1	1	1	1	1	3	3	1	1	1	2	2	1	2	1	2	2	2	2	3	2	1	1	1	1	1	2	1	1	1	2	2	1	1	1
67	Consultant Endocrinologist	AUS	Less than 5 Years	70-79 patients	1	1	2	1	1	1	1	1	2	1	1	1	1	1	1	1	1	1	1	2	1	2	2	1	2	1	1	1	1	1	1	2	1	2	1	1	1	1	1	1	1	1
68	Consultant Cardiologist	BRA	More than 10 years	20-29 patients	1	2	2	2	1	1	1	1	1	2	1	2	1	2	1	1	1	2	2	2	2	2	2	2	3	2	2	2	2	2	1	1	2	2	2	2	2	2	2	2	1	1
69	Consultant Cardiologist	BRA	More than 10 years	50-59 patients	1	1	1	1	1	1	1	1	1	1	1	1	1	1	1	1	1	1	1	3	2	3	3	1	3	2	3	3	2	2	2	2	2	2	1	1	1	1	1	1	1	1
70	Consultant Cardiologist	MEX	More than 10 years	60-69 patients	3	4	4	4	4	4	4	3	3	4	4	4	4	4	4	1	1	1	1	2	2	2	2	3	2	1	1	1	1	2	2	1	2	3	1	1	2	1	2	1	1	1
71	Consultant Nephrologist	BRA	More than 10 years	70-79 patients	2	1	2	2	1	1	1	1	2	2	2	2	2	2	2	2	2	2	2	2	2	3	2	3	3	2	2	2	2	1	2	1	3	2	2	2	2	1	2	1	1	1
72	Consultant Cardiologist	BRA	More than 10 years	100+ patients	1	1	1	1	1	1	1	2	2	1	1	1	1	1	1	1	1	1	1	1	1	2	2	2	1	1	1	2	2	3	2	2	3	1	1	1	1	1	1	1	1	1
73	Consultant Nephrologist	TUR	6 - 10 years	100+ patients	1	1	1	1	1	1	1	1	1	1	1	1	1	1	1	1	1	1	2	1	1	1	3	1	1	1	1	4	1	1	1	1	1	1	1	1	1	1	1	1	1	1
74	Consultant Endocrinologist	TUR	More than 10 years	90-99 patients	1	1	1	1	1	1	1	1	1	1	1	1	1	1	1	1	1	1	1	1	1	1	1	1	1	1	1	2	1	2	1	1	1	2	1	1	1	1	1	1	1	1
75	Consultant Cardiologist	BRA	More than 10 years	20-29 patients	2	1	2	2	1	1	2	2	3	2	1	2	2	1	2	1	1	2	1	2	3	2	2	2	2	2	1	1	2	3	1	1	2	2	2	1	2	2	1	1	1	1
76	Primary Care Physician	KOR	6 - 10 years	40-49 patients	4	1	2	1	2	1	2	2	3	1	1	2	2	3	1	2	2	2	2	3	2	2	3	2	3	2	1	1	1	1	1	1	2	1	2	1	1	1	1	2	1	1
77	Consultant Cardiologist	TUR	Less than 5 Years	20-29 patients	1	1	1	1	1	1	1	1	2	1	1	1	1	2	2	1	1	1	1	1	2	1	1	2	2	1	1	2	1	1	2	2	2	1	2	2	1	1	1	1	1	1
78	Primary Care Physician	KOR	6 - 10 years	30-39 patients	4	1	2	1	1	1	1	1	1	1	1	1	1	1	1	1	1	2	1	2	2	1	1	2	1	1	1	1	2	1	1	1	1	1	1	1	1	1	1	1	1	1
79	Consultant Endocrinologist	BRA	More than 10 years	30-39 patients	1	1	2	1	1	1	1	1	2	1	1	1	2	1	1	1	1	1	1	1	1	2	4	2	1	4	1	1	1	1	1	1	2	2	1	1	1	1	1	1	1	1
80	Primary Care Physician	KOR	More than 10 years	100+ patients	1	2	2	1	1	2	2	2	1	1	2	2	1	1	1	2	2	1	1	1	2	1	1	2	2	2	2	1	1	2	2	1	1	1	1	2	2	2	2	2	1	1
81	Primary Care Physician	ARG	More than 10 years	10-19 patients	1	2	2	2	2	2	2	2	2	2	2	3	2	3	1	2	2	2	2	2	2	2	3	2	2	1	2	2	2	1	3	2	2	2	1	1	2	1	2	2	1	2
82	Consultant Cardiologist	EGY	Less than 5 Years	80-89 patients	1	2	2	2	2	2	2	2	2	1	1	1	1	2	2	1	1	1	1	2	2	2	2	2	2	1	1	3	2	2	1	2	2	2	2	2	3	2	2	2	1	2
83	Primary Care Physician	SGP	More than 10 years	100+ patients	1	1	1	1	1	1	2	2	1	2	1	1	2	1	2	1	2	1	1	2	2	2	2	2	2	1	1	3	1	2	1	2	2	2	1	1	1	1	2	1	1	1
84	Primary Care Physician	ARG	More than 10 years	30-39 patients	1	1	2	1	1	1	2	1	2	1	1	1	1	2	1	1	1	2	1	2	1	2	4	2	4	1	1	4	2	1	1	1	1	1	2	1	1	1	1	1	1	1
85	Primary Care Physician	SGP	More than 10 years	100+ patients	1	1	1	1	2	2	2	1	1	2	1	1	2	2	1	1	1	1	1	1	2	2	2	2	2	2	1	2	1	2	3	1	2	2	1	1	1	2	1	1	1	1
86	Primary Care Physician	KOR	More than 10 years	10-19 patients	3	3	4	3	2	3	4	3	4	2	1	2	3	2	2	2	2	3	2	2	2	2	2	3	1	2	2	2	2	2	3	2	2	1	2	2	2	3	2	2	2	2
87	Consultant Endocrinologist	ARG	6 - 10 years	50-59 patients	3	1	2	3	1	2	2	2	2	1	1	2	2	1	1	1	1	1	2	2	2	2	2	1	3	2	2	3	1	2	2	2	2	2	2	2	2	2	2	1	1	1
88	Primary Care Physician	ARG	More than 10 years	70-79 patients	2	1	1	1	1	1	2	2	2	2	1	2	1	2	2	1	2	2	2	3	3	2	3	2	3	2	1	2	2	1	2	1	2	2	2	1	1	1	2	2	1	2
89	Primary Care Physician	KOR	More than 10 years	40-49 patients	1	1	1	2	1	2	2	1	2	1	1	1	1	2	1	1	1	1	1	2	2	2	1	2	2	1	1	2	1	1	2	1	1	1	1	2	1	2	1	1	1	1
90	Consultant Endocrinologist	KOR	6 - 10 years	60-69 patients	2	2	1	2	2	2	2	2	1	2	2	2	1	1	3	2	2	1	1	2	1	2	3	2	2	2	2	2	1	1	2	2	2	3	1	2	2	1	1	2	2	2
91	Primary Care Physician	KOR	More than 10 years	30-39 patients	2	2	2	2	2	2	2	2	2	2	2	2	2	2	2	2	2	2	2	2	2	2	2	2	2	2	2	2	2	2	2	2	2	2	2	2	2	2	2	2	2	2
92	Primary Care Physician	KOR	More than 10 years	70-79 patients	1	1	1	1	1	1	2	2	1	2	1	1	2	1	1	2	2	1	1	1	2	2	1	1	1	1	1	2	2	2	1	1	1	2	2	2	1	2	2	2	1	2
93	Consultant Cardiologist	MEX	6 - 10 years	10-19 patients	1	1	1	1	2	2	2	2	2	1	1	3	2	3	2	1	2	2	2	3	3	2	2	1	2	2	2	2	1	1	1	1	2	2	2	1	1	1	1	1	2	2
94	Primary Care Physician	ARG	6 - 10 years	10-19 patients	2	2	2	1	1	1	1	1	2	1	1	1	1	2	1	1	1	2	2	2	2	2	4	2	3	2	2	2	2	2	3	2	2	2	2	2	2	2	2	2	2	2
95	Consultant Endocrinologist	AUS	More than 10 years	50-59 patients	1	2	2	1	1	1	2	1	1	2	1	1	1	2	1	1	1	1	1	1	2	1	1	2	2	1	1	2	1	1	2	1	1	1	1	2	2	2	2	2	2	2
96	Consultant Cardiologist	SGP	6 - 10 years	100+ patients	1	1	1	1	1	1	1	1	2	1	1	1	1	1	1	1	1	1	1	1	1	1	1	2	2	1	1	2	1	1	1	1	1	2	2	2	1	1	1	1	1	2
97	Consultant Endocrinologist	SGP	6 - 10 years	100+ patients	1	1	1	1	1	1	2	1	2	2	1	1	2	2	1	1	1	1	1	1	2	2	2	2	2	2	1	3	2	1	1	1	2	2	1	1	2	2	1	1	1	1
98	Consultant Nephrologist	SGP	6 - 10 years	100+ patients	1	1	1	1	1	2	2	1	1	1	1	1	1	2	1	1	1	1	1	2	1	1	1	2	2	1	1	2	1	1	1	2	3	2	1	1	1	1	1	1	1	1
99	Consultant Nephrologist	SGP	More than 10 years	100+ patients	1	2	2	2	2	1	3	2	2	1	1	1	2	3	2	2	1	1	1	2	1	2	2	1	2	1	2	2	2	1	2	2	2	1	1	1	1	1	1	1	1	1
100	Consultant Endocrinologist	SGP	More than 10 years	100+ patients	1	1	1	2	1	1	1	1	1	1	1	2	2	1	1	1	1	1	2	2	2	2	1	1	1	1	1	2	1	1	1	1	1	2	1	2	1	1	1	2	1	1
101	Primary Care Physician	KOR	More than 10 years	50-59 patients	1	1	1	1	1	2	2	2	2	1	1	1	1	1	1	1	1	1	1	1	2	2	2	2	2	1	1	3	2	2	2	2	2	2	3	2	2	2	2	2	2	2
102	Consultant Nephrologist	TUR	More than 10 years	40-49 patients	1	1	2	2	1	1	2	1	1	2	1	2	1	2	2	1	2	1	2	2	2	3	2	2	2	2	2	2	2	2	2	1	1	2	2	1	1	1	1	1	1	1
103	Consultant Nephrologist	TUR	More than 10 years	40-49 patients	1	1	2	1	1	1	2	1	1	1	1	1	1	1	1	1	1	2	1	2	2	2	2	1	2	2	1	2	1	1	1	1	1	1	1	1	1	1	1	1	1	1
104	Consultant Endocrinologist	AUS	More than 10 years	70-79 patients	1	1	1	1	1	1	2	2	2	1	1	2	2	3	2	1	1	2	2	2	2	2	2	2	3	2	1	2	2	1	1	2	2	2	2	2	2	2	2	2	2	2
105	Primary Care Physician	SGP	More than 10 years	100+ patients	1	1	1	1	1	1	2	1	2	1	1	2	2	2	1	1	1	1	1	2	2	1	1	2	4	1	1	2	1	1	1	1	2	3	2	2	1	1	1	1	1	1
106	Consultant Nephrologist	SGP	6 - 10 years	100+ patients	1	1	1	1	3	2	2	2	1	1	1	1	1	1	1	1	1	1	1	2	2	1	2	2	2	1	1	2	2	1	1	2	1	2	1	1	3	2	1	1	1	1
107	Consultant Nephrologist	TUR	More than 10 years	40-49 patients	1	1	1	1	2	2	1	1	1	1	1	1	2	1	2	1	2	1	2	1	1	2	1	2	1	1	1	1	1	1	2	1	1	2	1	1	1	1	1	1	1	1
108	Primary Care Physician	SGP	More than 10 years	10-19 patients	1	2	1	1	2	2	1	2	2	2	1	2	2	2	1	2	1	1	1	1	2	2	1	2	2	2	1	2	1	1	1	1	1	2	1	2	1	1	1	1	1	1
109	Consultant Nephrologist	BRA	More than 10 years	100+ patients	1	2	2	2	1	1	1	1	2	1	1	1	1	1	1	1	1	1	1	1	1	1	2	2	3	2	1	2	2	2	2	2	2	3	2	2	2	2	2	1	1	1
110	Primary Care Physician	ARG	Less than 5 Years	20-29 patients	2	1	2	2	1	1	2	2	2	2	1	1	1	2	1	1	2	2	2	2	3	2	2	2	3	4	2	2	2	1	1	1	2	1	1	1	1	2	2	1	2	2
111	Consultant Endocrinologist	TUR	More than 10 years	60-69 patients	1	1	1	1	1	1	1	1	1	1	1	1	1	1	1	1	1	2	2	2	3	2	2	1	1	1	1	2	2	2	2	1	1	1	2	1	2	1	1	1	2	2
112	Consultant Cardiologist	ARG	More than 10 years	60-69 patients	1	1	1	1	1	1	1	1	1	1	1	1	1	1	1	1	1	1	1	1	1	2	2	1	2	2	1	1	2	1	1	1	1	2	2	1	1	1	1	1	1	1
113	Consultant Endocrinologist	MEX	More than 10 years	50-59 patients	1	3	1	1	1	1	2	1	2	1	1	1	1	1	1	1	1	1	1	1	1	3	1	3	2	1	1	2	1	1	1	1	1	2	1	1	1	1	1	1	1	1
114	Consultant Cardiologist	SGP	6 - 10 years	90-99 patients	3	1	1	1	1	1	2	2	2	1	1	2	2	3	1	1	1	1	1	1	1	1	1	2	3	1	1	1	1	1	1	1	2	1	2	1	2	1	1	1	1	1
115	Primary Care Physician	SGP	More than 10 years	10-19 patients	1	2	2	1	2	2	2	1	3	2	1	1	2	2	1	1	2	2	2	2	2	3	3	3	1	1	1	2	2	1	1	1	2	2	2	2	1	1	1	1	1	1
116	Primary Care Physician	SGP	More than 10 years	100+ patients	1	1	1	1	2	1	1	1	2	1	1	1	1	2	2	1	1	2	1	1	1	1	2	2	2	1	1	1	2	1	2	1	2	2	1	1	1	1	2	1	1	1
117	Primary Care Physician	KOR	More than 10 years	80-89 patients	1	2	2	2	2	1	2	2	2	2	1	2	2	2	2	2	2	1	1	1	2	2	2	2	2	2	2	2	2	2	2	2	2	2	2	2	2	2	2	2	2	2
118	Consultant Cardiologist	MEX	More than 10 years	10-19 patients	1	1	1	1	1	1	1	1	1	1	1	1	1	1	1	1	1	1	1	1	1	1	1	1	1	1	1	1	1	1	1	1	1	1	1	1	1	1	1	1	1	1
119	Consultant Endocrinologist	SGP	More than 10 years	70-79 patients	1	1	1	2	1	2	2	1	1	1	1	2	1	2	1	1	1	1	1	2	2	2	2	2	2	1	1	2	1	2	1	1	1	3	1	2	2	2	2	1	1	1
120	Consultant Nephrologist	SGP	6 - 10 years	100+ patients	1	1	1	1	1	1	1	1	1	1	1	1	2	1	1	1	1	1	1	2	2	1	1	1	2	1	1	2	1	2	1	1	1	3	1	2	1	2	1	1	1	2
121	Consultant Cardiologist	SGP	More than 10 years	100+ patients	2	1	1	1	1	1	1	1	1	1	1	1	1	2	1	1	1	1	1	1	1	1	1	2	2	1	1	2	1	1	1	1	1	1	1	1	1	1	1	2	2	2
122	Consultant Cardiologist	SGP	More than 10 years	40-49 patients	1	2	1	1	1	1	3	1	2	1	1	2	2	2	1	1	1	2	1	1	1	1	2	2	2	1	1	1	1	1	1	1	2	2	1	2	2	2	2	1	1	1
123	Primary Care Physician	SGP	6 - 10 years	100+ patients	1	1	1	1	1	1	2	2	2	1	2	1	1	2	1	1	1	1	1	2	2	3	2	2	1	3	2	1	1	1	1	1	2	1	1	1	1	1	1	1	1	1
124	Consultant Nephrologist	TUR	6 - 10 years	100+ patients	1	1	1	1	1	1	1	1	1	1	1	1	2	1	1	1	1	1	1	1	1	2	3	2	1	1	1	1	1	3	1	1	2	1	2	1	2	2	1	1	1	1
125	Primary Care Physician	TWN	More than 10 years	40-49 patients	1	2	1	2	1	1	2	1	2	1	2	2	1	2	1	1	1	1	1	1	2	2	2	1	2	1	1	2	1	1	1	1	2	1	1	1	1	2	2	2	1	1
126	Primary Care Physician	TWN	More than 10 years	50-59 patients	1	2	2	2	1	1	2	2	2	1	2	2	1	2	1	1	2	1	1	1	2	2	2	2	2	1	1	3	2	1	2	2	1	2	2	1	1	2	2	1	2	1
127	Primary Care Physician	TWN	More than 10 years	100+ patients	1	1	2	2	1	1	2	2	2	1	1	2	1	1	2	2	2	2	1	1	2	2	3	2	2	1	1	2	1	1	2	2	2	1	2	2	1	2	2	2	1	2
128	Primary Care Physician	TWN	More than 10 years	80-89 patients	1	1	1	1	1	1	1	1	2	1	1	1	1	2	1	1	1	2	2	2	1	2	3	2	2	1	1	2	2	1	2	1	1	1	1	2	2	1	1	1	2	1
129	Primary Care Physician	TWN	More than 10 years	100+ patients	1	1	2	2	1	1	2	2	1	1	1	1	2	2	1	1	1	1	1	2	2	1	3	2	2	1	1	3	2	1	1	2	1	2	1	1	1	2	2	2	1	1
130	Primary Care Physician	TWN	More than 10 years	100+ patients	1	1	2	2	1	1	2	2	3	1	2	2	2	2	2	1	1	2	1	1	1	2	2	2	2	2	1	2	2	1	2	1	2	2	1	2	2	2	1	2	1	1
131	Primary Care Physician	TWN	More than 10 years	40-49 patients	1	1	1	1	1	2	2	1	2	1	1	1	2	2	1	1	1	1	1	2	2	2	2	1	2	1	2	2	1	1	2	1	2	2	1	1	1	1	1	1	2	1
132	Primary Care Physician	TWN	More than 10 years	10-19 patients	1	2	2	1	2	2	2	2	1	2	2	1	2	2	2	2	2	2	1	2	2	2	2	2	2	1	2	2	2	1	2	2	2	2	2	2	2	1	1	2	1	1
133	Consultant Endocrinologist	TWN	More than 10 years	100+ patients	1	1	2	1	2	1	3	2	2	1	2	2	1	2	2	2	1	2	1	2	2	3	2	2	3	1	2	2	2	1	1	1	3	2	2	2	1	1	2	2	1	2
134	Consultant Endocrinologist	TWN	More than 10 years	100+ patients	2	2	3	2	1	1	2	2	2	2	2	2	1	3	2	2	2	1	1	1	2	2	2	2	2	1	2	3	1	1	2	2	1	2	2	1	1	2	2	2	1	2
135	Consultant Endocrinologist	TWN	More than 10 years	100+ patients	1	1	1	1	1	1	4	2	1	1	2	1	1	3	1	1	1	1	1	1	1	3	2	2	4	2	1	1	3	1	2	2	2	4	1	1	3	1	1	1	1	1
136	Consultant Endocrinologist	TWN	More than 10 years	100+ patients	1	1	2	2	1	1	3	1	2	2	1	1	2	3	1	2	2	1	1	1	1	3	2	3	3	2	1	4	2	1	1	1	2	3	2	1	1	2	3	1	1	2

137	Consultant Endocrinologist	TWN	More than 10 years	100+ patients	1	2	1	1	2	1	2	2	2	1	2	2	2	3	1	1	2	1	1	1	2	2	1	2	3	2	1	3	2	2	2	1	3	3	1	2	1	2	1	1	1	2
138	Consultant Cardiologist	TWN	More than 10 years	80-89 patients	1	1	2	1	2	1	2	2	2	2	1	2	2	2	2	2	1	2	1	2	2	2	2	2	2	1	1	2	1	2	2	2	2	1	1	2	1	2	2	2	2	2
139	Consultant Cardiologist	TWN	More than 10 years	60-69 patients	1	1	2	1	1	2	1	2	2	1	1	1	1	3	2	1	1	1	1	1	2	1	2	2	2	2	1	2	1	2	2	2	2	3	1	2	1	1	1	1	2	2
140	Consultant Cardiologist	TWN	More than 10 years	100+ patients	1	1	1	1	1	1	1	1	1	1	1	1	1	1	1	1	1	1	1	1	1	1	2	2	1	1	1	2	1	1	1	1	1	2	1	1	1	1	1	1	1	1
141	Consultant Cardiologist	TWN	More than 10 years	50-59 patients	1	2	1	1	1	1	2	2	2	1	1	1	3	3	1	1	1	3	1	1	1	1	2	2	3	1	1	1	1	1	1	1	1	2	1	1	1	1	1	1	1	1
142	Consultant Cardiologist	TWN	More than 10 years	50-59 patients	1	2	1	2	1	1	3	2	3	1	1	2	1	2	2	1	2	1	1	1	1	2	2	2	2	1	1	2	2	1	1	2	2	1	3	3	2	1	2	1	2	1
143	Consultant Nephrologist	TWN	More than 10 years	100+ patients	1	3	1	1	1	1	1	1	1	1	1	2	2	3	1	1	1	2	1	1	1	2	2	3	4	2	1	2	2	1	1	2	1	2	1	2	2	2	1	1	1	1
144	Consultant Nephrologist	TWN	More than 10 years	100+ patients	1	2	2	2	1	1	1	2	1	1	1	1	2	2	1	2	2	2	2	2	2	2	2	2	3	2	1	1	2	1	2	1	2	2	2	2	3	2	2	3	2	2
145	Consultant Nephrologist	TWN	More than 10 years	100+ patients	2	1	1	1	1	1	2	1	1	1	1	1	1	2	1	1	1	1	1	2	1	2	2	1	2	1	1	2	2	1	1	2	1	2	3	1	2	2	1	2	1	1
146	Primary Care Physician	THA	More than 10 years	10-19 patients	1	1	1	1	1	1	1	1	2	2	1	1	1	2	1	1	1	2	2	2	2	2	3	1	1	1	1	3	1	1	1	1	1	1	1	1	1	1	1	1	1	1
147	Primary Care Physician	THA	6 - 10 years	40-49 patients	2	2	2	2	2	2	2	2	2	2	2	2	2	2	2	1	2	2	2	2	2	2	2	2	2	2	2	1	2	2	2	2	1	2	2	2	2	2	1	2	2	2
148	Primary Care Physician	THA	Less than 5 Years	40-49 patients	1	1	1	1	1	1	1	1	1	1	1	1	2	2	2	1	1	2	2	3	2	2	2	2	2	2	1	2	2	1	1	1	2	2	1	1	1	1	1	1	1	1
149	Primary Care Physician	TWN	More than 10 years	100+ patients	1	1	4	1	1	1	4	1	2	2	1	1	1	2	1	1	1	1	1	1	1	1	1	2	2	2	1	1	1	1	1	2	2	3	1	1	1	1	1	1	1	1
150	Primary Care Physician	SGP	More than 10 years	50-59 patients	1	1	1	1	1	1	1	2	1	2	1	1	2	2	1	1	1	1	1	1	1	1	2	1	2	1	1	3	1	1	1	2	1	2	1	2	1	1	1	1	1	1
151	Primary Care Physician	SGP	More than 10 years	30-39 patients	1	2	2	2	2	2	2	2	2	2	2	2	2	2	1	1	2	2	2	2	2	2	3	2	2	1	1	3	1	2	1	1	1	2	1	2	2	2	1	1	2	1
152	Consultant Endocrinologist	ARG	More than 10 years	40-49 patients	3	2	3	1	1	1	2	2	1	1	1	2	1	1	2	1	2	1	1	2	2	1	1	1	1	1	1	2	1	1	1	1	1	1	1	1	1	2	1	2	1	1
153	Consultant Endocrinologist	KOR	More than 10 years	20-29 patients	1	2	1	2	2	2	3	2	3	2	2	1	2	2	2	1	2	2	2	3	2	2	2	2	2	1	1	2	2	2	2	2	3	2	2	2	2	2	2	1	1	1
154	Consultant Cardiologist	KOR	More than 10 years	20-29 patients	1	1	1	1	1	1	1	1	1	1	1	1	1	1	1	1	1	1	1	1	3	1	3	2	2	1	1	3	2	1	1	1	1	2	1	1	1	1	1	1	1	1
155	Consultant Endocrinologist	KOR	More than 10 years	100+ patients	1	1	1	1	1	1	1	1	1	1	1	1	1	1	1	1	1	1	1	1	1	1	1	1	1	1	1	3	1	1	1	1	1	2	1	1	1	1	1	1	1	1
156	Consultant Endocrinologist	THA	More than 10 years	100+ patients	2	1	1	1	1	1	1	1	1	1	2	2	2	2	1	1	1	1	1	2	2	2	2	2	2	2	1	3	1	2	1	1	1	1	1	1	1	1	1	1	1	1
157	Consultant Nephrologist	KOR	More than 10 years	100+ patients	1	1	1	1	1	1	1	1	1	1	1	1	1	1	1	1	1	1	1	1	1	1	1	1	3	1	1	1	2	1	1	1	1	2	2	1	1	1	1	1	1	1
158	Primary Care Physician	KOR	Less than 5 Years	100+ patients	2	2	2	2	2	2	2	2	2	2	2	2	1	2	2	2	2	2	1	2	2	2	2	2	2	2	2	3	2	2	2	2	2	2	2	2	2	2	2	2	2	2
159	Consultant Endocrinologist	KOR	6 - 10 years	50-59 patients	1	2	2	1	1	1	2	1	1	1	1	1	1	1	1	1	1	1	1	3	2	3	2	2	2	1	3	2	2	2	2	2	3	2	2	2	2	2	2	1	1	1
160	Primary Care Physician	THA	Less than 5 Years	60-69 patients	1	2	2	1	1	1	2	1	2	1	1	1	2	3	2	1	3	2	1	2	1	2	2	2	3	1	1	3	1	1	1	1	1	1	1	1	1	2	1	1	1	1
161	Consultant Nephrologist	TUR	6 - 10 years	100+ patients	1	1	1	1	1	1	1	1	1	1	1	1	1	1	1	1	1	1	1	1	1	1	1	2	2	1	1	4	1	2	2	1	1	1	2	1	2	1	1	1	1	1
162	Consultant Endocrinologist	THA	More than 10 years	100+ patients	1	1	1	1	1	1	1	1	1	1	1	1	1	1	1	1	1	1	1	3	2	2	2	2	2	2	1	1	1	1	1	1	1	1	1	1	2	2	2	1	1	1
163	Primary Care Physician	THA	Less than 5 Years	40-49 patients	1	1	1	1	2	1	1	1	2	1	2	1	2	3	1	1	1	1	2	3	2	2	4	3	3	2	1	2	1	1	1	1	1	1	1	1	1	1	1	1	1	1
164	Consultant Nephrologist	THA	More than 10 years	100+ patients	1	1	1	1	1	1	1	2	1	1	1	1	1	1	1	1	1	1	1	1	2	2	1	2	1	2	1	3	1	1	1	1	1	1	1	2	2	1	1	1	1	1
165	Consultant Nephrologist	TWN	More than 10 years	100+ patients	2	1	1	1	1	1	2	2	1	1	1	1	2	3	1	1	1	1	1	1	1	1	1	2	2	1	1	2	1	1	1	1	1	1	1	1	1	2	1	1	1	1
166	Consultant Nephrologist	TWN	More than 10 years	100+ patients	1	1	1	1	1	1	1	1	1	2	2	1	1	1	2	1	1	1	1	1	2	2	1	2	2	1	1	3	2	2	2	1	2	2	2	2	2	1	1	2	2	1
167	Consultant Cardiologist	ARG	6 - 10 years	10-19 patients	2	1	1	1	1	1	1	2	2	1	1	2	2	3	2	2	2	2	1	2	3	2	3	2	1	1	1	3	1	2	1	2	3	4	2	1	1	2	2	2	1	1
168	Consultant Endocrinologist	THA	More than 10 years	50-59 patients	1	1	1	1	1	1	2	2	2	1	1	2	3	2	1	1	2	1	2	2	3	3	3	2	3	2	2	3	2	2	2	2	2	1	1	2	2	2	1	1	1	1
169	Consultant Cardiologist	THA	More than 10 years	90-99 patients	1	1	1	1	1	1	1	1	1	1	1	1	1	2	2	1	1	1	1	1	2	2	2	1	1	2	1	3	1	1	2	1	1	1	2	1	2	1	1	1	1	1
170	Consultant Cardiologist	THA	More than 10 years	20-29 patients	1	1	1	1	1	1	1	1	2	1	1	1	1	1	1	1	1	1	1	1	2	2	2	1	2	1	1	1	1	1	1	1	1	1	1	1	2	1	1	1	1	1
171	Consultant Nephrologist	MEX	More than 10 years	80-89 patients	4	1	1	1	1	1	1	2	2	1	1	1	1	1	1	1	1	1	1	1	1	1	1	1	2	1	1	1	1	1	1	1	1	2	1	1	1	1	1	1	2	1
172	Consultant Nephrologist	BRA	Less than 5 Years	100+ patients	1	1	1	1	1	1	1	1	1	1	1	1	1	1	1	1	1	1	1	1	1	1	1	1	3	2	3	2	1	1	1	1	1	1	1	1	1	1	1	1	1	1
173	Consultant Cardiologist	ARG	More than 10 years	50-59 patients	2	3	1	1	1	1	1	2	1	1	1	2	1	2	2	2	1	1	1	2	1	2	2	2	3	2	1	2	1	2	2	1	2	3	1	1	1	2	3	2	1	1
174	Consultant Nephrologist	ARG	6 - 10 years	100+ patients	1	1	1	1	1	1	2	1	1	1	1	1	1	1	1	1	1	1	1	1	1	1	1	1	2	1	1	1	1	1	1	1	1	1	1	1	1	1	1	1	1	1
175	Consultant Nephrologist	ARG	6 - 10 years	20-29 patients	4	4	1	2	1	1	1	1	1	1	1	2	2	2	1	1	1	2	2	2	2	2	1	1	2	1	1	3	1	2	1	1	2	2	1	1	1	2	1	1	1	1
176	Consultant Cardiologist	ARG	Less than 5 Years	10-19 patients	3	1	1	1	1	2	2	1	2	2	1	2	2	2	1	1	2	2	2	2	2	2	1	2	2	2	1	2	2	2	2	2	3	2	1	1	2	2	2	2	2	2
177	Primary Care Physician	ARG	Less than 5 Years	20-29 patients	1	1	1	1	1	2	2	2	2	1	1	1	1	2	1	1	1	2	2	2	2	2	3	2	3	2	1	2	1	1	1	1	1	1	1	1	1	1	1	1	1	1
178	Consultant Cardiologist	ARG	More than 10 years	60-69 patients	1	1	1	1	1	1	2	1	2	1	1	1	1	1	1	1	1	2	1	1	1	1	1	2	2	3	1	2	1	1	1	1	1	1	1	1	1	1	1	1	1	1
179	Primary Care Physician	GTM	More than 10 years	100+ patients	1	1	1	1	1	1	1	1	1	1	1	1	1	1	1	1	1	1	1	1	2	2	2	2	2	3	1	1	1	1	1	1	1	1	1	1	1	1	1	1	1	1
180	Consultant Endocrinologist	GTM	Less than 5 Years	20-29 patients	1	1	1	1	1	1	1	1	1	1	1	1	1	1	1	1	1	1	1	1	1	1	1	1	1	1	1	2	1	1	1	1	1	1	1	1	1	1	2	1	1	1
181	Consultant Nephrologist	ARG	6 - 10 years	100+ patients	1	1	1	1	1	1	1	2	1	1	1	1	1	1	1	1	1	1	1	1	1	3	1	3	1	4	1	1	1	1	1	1	1	1	1	1	1	1	1	1	1	1
182	Consultant Nephrologist	ARG	6 - 10 years	50-59 patients	1	2	2	2	1	1	1	3	2	1	1	1	2	2	2	1	1	2	2	2	1	1	2	2	3	2	2	2	2	1	2	2	2	2	1	1	1	2	2	1	1	2
183	Consultant Endocrinologist	ARG	6 - 10 years	80-89 patients	1	2	1	1	1	1	1	2	1	1	1	1	1	1	1	1	1	1	1	1	1	2	2	1	2	1	1	2	1	1	1	1	1	1	1	1	1	1	1	1	1	1
184	Consultant Nephrologist	KOR	More than 10 years	100+ patients	1	1	2	1	1	2	2	1	3	2	1	1	2	3	1	2	1	1	1	2	2	2	2	2	3	2	1	2	1	2	2	2	2	2	2	1	2	3	2	1	2	2
185	Consultant Endocrinologist	GTM	More than 10 years	30-39 patients	1	1	1	1	1	1	1	1	1	1	1	1	2	2	1	1	1	1	1	1	1	2	2	1	2	1	1	3	1	1	1	1	1	3	1	1	1	1	1	1	1	1
186	Consultant Endocrinologist	ARG	Less than 5 Years	30-39 patients	1	1	1	1	1	1	2	1	1	1	1	1	2	2	1	1	1	1	1	2	1	2	1	2	2	1	1	2	1	1	2	1	2	3	1	1	1	2	2	2	1	1
187	Consultant Endocrinologist	ARG	Less than 5 Years	50-59 patients	1	1	1	1	1	1	1	1	1	1	1	1	3	3	1	1	1	1	1	1	1	1	1	1	3	3	1	2	1	1	1	1	1	1	1	1	1	1	1	1	1	1
188	Consultant Cardiologist	KOR	More than 10 years	100+ patients	1	1	1	1	1	1	2	1	2	1	1	1	2	2	1	1	1	1	1	1	1	3	2	2	2	1	1	3	2	2	1	2	2	1	2	2	2	2	1	1	2	2
189	Primary Care Physician	ARG	More than 10 years	20-29 patients	1	1	1	1	1	1	2	2	2	1	2	2	2	3	1	1	2	2	1	2	2	2	2	2	2	1	1	2	1	1	1	1	1	1	1	1	1	1	1	1	1	1
190	Consultant Nephrologist	ARG	6 - 10 years	100+ patients	1	1	1	1	1	1	1	1	1	1	1	2	1	2	1	1	1	1	1	1	1	1	1	1	3	2	1	1	1	1	1	1	1	1	1	1	1	1	1	1	1	1
191	Primary Care Physician	ARG	More than 10 years	20-29 patients	1	1	1	1	1	1	2	1	1	1	2	1	2	2	1	1	2	2	2	2	2	2	2	2	2	1	1	1	1	1	1	1	1	2	1	2	1	2	1	1	1	1
192	Consultant Nephrologist	ARG	More than 10 years	100+ patients	1	1	2	1	1	2	2	2	2	1	1	1	3	3	1	1	1	1	1	2	1	1	2	2	2	2	1	2	1	3	3	1	2	2	1	1	3	2	3	1	2	1
193	Consultant Endocrinologist	SGP	More than 10 years	90-99 patients	1	1	1	1	1	1	1	1	2	1	1	2	2	2	1	1	1	1	1	1	1	3	2	2	2	2	1	2	1	1	1	1	1	1	1	1	1	1	1	1	1	1
194	Consultant Endocrinologist	SGP	More than 10 years	100+ patients	1	1	1	1	1	1	2	3	1	1	1	1	1	1	1	1	1	2	1	1	1	2	1	2	1	2	1	1	1	1	1	1	1	1	1	1	1	1	1	1	1	1
195	Consultant Cardiologist	SGP	More than 10 years	100+ patients	1	1	2	1	1	1	2	2	2	1	1	1	1	2	1	1	1	1	1	1	1	2	1	1	1	2	1	1	2	1	1	2	2	1	2	1	1	1	1	1	1	1
196	Primary Care Physician	KOR	More than 10 years	50-59 patients	1	2	2	2	1	1	2	1	2	2	1	1	2	2	1	2	1	1	1	2	2	2	2	2	2	2	1	2	2	1	2	2	2	2	1	2	2	2	1	2	1	1
197	Consultant Nephrologist	THA	More than 10 years	100+ patients	1	1	1	1	1	1	1	1	1	1	1	1	1	1	1	1	1	1	1	1	1	1	1	1	1	2	1	3	1	1	1	1	1	1	1	1	1	1	1	1	1	1
198	Consultant Nephrologist	THA	6 - 10 years	100+ patients	1	1	1	1	1	1	2	1	1	1	1	1	1	1	1	1	1	1	1	1	1	1	4	1	1	1	1	1	1	1	1	1	1	1	1	1	1	1	1	1	1	1
199	Consultant Nephrologist	KOR	More than 10 years	100+ patients	1	1	1	1	1	1	2	1	1	2	1	1	1	1	1	1	2	1	1	2	2	2	2	2	2	1	1	3	2	2	2	2	2	2	1	2	2	2	1	1	2	2
200	Primary Care Physician	SGP	More than 10 years	100+ patients	1	2	2	1	2	1	2	1	1	1	1	1	1	1	1	1	1	1	1	2	1	1	1	2	3	2	1	3	2	2	1	1	1	2	2	1	2	1	1	1	1	1
201	Consultant Cardiologist	THA	More than 10 years	80-89 patients	1	2	1	2	1	1	1	2	1	1	2	2	2	1	1	1	1	1	2	1	1	2	1	1	2	2	1	1	1	2	2	2	1	2	1	1	1	1	1	1	2	1
202	Primary Care Physician	GTM	More than 10 years	10-19 patients	2	3	2	2	2	1	2	2	2	2	1	2	2	2	1	1	2	2	2	2	2	2	2	2	2	3	1	2	2	2	1	1	2	2	1	1	1	1	1	1	1	1
203	Primary Care Physician	GTM	Less than 5 Years	10-19 patients	4	1	2	1	1	1	2	2	3	1	2	1	2	2	1	1	1	2	1	2	1	1	2	2	3	1	1	1	1	1	1	1	1	2	1	1	1	1	1	1	1	1
204	Primary Care Physician	GTM	6 - 10 years	20-29 patients	2	1	1	2	1	1	1	1	1	1	1	2	1	2	2	1	2	2	2	2	2	2	2	2	3	2	1	2	1	1	1	1	1	2	1	1	1	1	1	1	1	1
205	Primary Care Physician	GTM	6 - 10 years	30-39 patients	1	1	1	1	1	1	1	1	2	1	1	2	1	1	1	1	1	1	1	1	1	2	2	2	2	2	1	2	1	1	1	1	1	2	1	1	1	1	1	1	1	1
206	Primary Care Physician	GTM	More than 10 years	80-89 patients	1	1	1	1	1	1	1	1	1	1	1	1	1	1	1	1	1	1	1	1	1	1	1	1	3	1	1	1	1	1	1	1	1	1	1	1	1	1	1	1	1	1
207	Primary Care Physician	GTM	Less than 5 Years	10-19 patients	1	1	1	1	1	1	1	1	1	1	1	1	1	2	1	1	1	1	1	1	1	1	2	1	4	1	1	3	1	1	1	1	1	2	1	1	1	2	1	1	1	1
208	Primary Care Physician	GTM	6 - 10 years	60-69 patients	1	1	1	1	1	1	2	1	2	1	1	1	2	2	1	1	2	1	2	2	2	1	2	2	2	1	1	1	1	2	1	1	1	1	1	1	1	1	1	1	1	1
209	Consultant Nephrologist	KOR	6 - 10 years	100+ patients	2	1	2	2	2	2	2	3	2	2	2	2	2	3	2	2	2	2	2	2	2	2	2	2	3	2	2	2	3	2	2	3	3	3	2	3	2	3	3	2	2	2
210	Primary Care Physician	GTM	Less than 5 Years	10-19 patients	2	1	1	1	1	1	2	1	1	1	1	1	1	1	1	1	1	1	1	1	1	2	1	1	4	1	1	1	1	1	1	1	1	2	1	1	1	1	1	1	1	1
211	Consultant Nephrologist	MEX	More than 10 years	100+ patients	1	1	1	1	1	1	2	2	1	1	1	1	1	1	1	1	1	1	1	2	1	2	2	2	2	2	1	2	1	1	2	1	1	1	1	1	1	2	1	1	1	1
212	Primary Care Physician	GTM	6 - 10 years	10-19 patients	1	1	2	2	1	1	1	1	2	1	1	2	1	1	1	1	1	2	1	2	2	2	2	2	3	1	2	2	2	2	1	1	1	3	1	1	1	1	1	1	1	1
213	Primary Care Physician	GTM	More than 10 years	10-19 patients	1	1	1	2	1	1	1	2	2	1	2	2	3	1	1	1	1	1	1	3	2	1	2	1	2	1	1	1	1	1	1	1	1	2	1	1	1	1	1	1	1	1
214	Consultant Nephrologist	EGY	More than 10 years	80-89 patients	1	1	1	2	2	1	2	1	1	2	2	3	2	3	1	1	1	2	2	2	2	1	2	2	2	2	2	2	1	1	1	1	3	2	4	2	1	1	1	1	1	1
215	Primary Care Physician	GTM	More than 10 years	10-19 patients	1	1	2	1	1	1	2	2	3	1	2	1	2	2	1	1	2	2	2	3	3	2	1	1	3	2	2	3	1	1	1	2	2	2	1	1	1	1	1	1	1	1
216	Primary Care Physician	GTM	6 - 10 years	40-49 patients	1	1	1	1	1	1	1	1	1	1	1	1	1	1	1	1	1	1	1	1	1	2	2	2	2	2	1	3	1	1	1	1	1	1	1	1	1	1	1	1	1	1
217	Consultant Cardiologist	KOR	More than 10 years	100+ patients	1	1	1	1	1	2	1	2	3	1	1	1	1	2	2	1	1	2	2	2	2	1	2	3	2	1	1	3	2	2	1	2	1	3	1	2	2	2	1	2	2	1
218	Primary Care Physician	GTM	Less than 5 Years	10-19 patients	4	2	2	1	1	1	3	1	4	1	1	1	1	1	1	1	1	2	2	2	2	3	3	2	4	1	1	3	1	1	1	1	1	1	1	1	1	1	1	2	1	1
219	Primary Care Physician	GTM	More than 10 years	10-19 patients	1	1	2	1	1	3	2	1	1	1	1	2	1	3	2	1	2	2	2	4	2	1	4	2	1	1	1	1	1	2	1	1	1	2	1	2	1	1	1	2	1	1
220	Primary Care Physician	THA	More than 10 years	10-19 patients	1	1	1	1	1	1	2	2	2	1	1	2	2	2	2	2	2	2	2	2	2	2	3	2	3	2	1	2	2	2	2	2	2	2	1	1	1	2	2	1	1	1
221	Consultant Nephrologist	THA	6 - 10 years	100+ patients	1	1	1	1	1	1	2	2	1	1	1	1	1	1	1	1	1	1	1	1	1	1	1	2	1	1	1	2	1	1	1	1	1	1	1	1	1	1	1	1	1	1
222	Consultant Nephrologist	TUR	More than 10 years	80-89 patients	4	4	3	3	4	4	4	4	4	4	4	3	4	3	4	1	2	2	2	2	2	3	3	3	2	1	1	2	2	2	2	2	2	2	2	1	2	2	2	2	2	2
223	Consultant Nephrologist	THA	Less than 5 Years	100+ patients	1	1	1	1	1	1	1	1	1	1	1	1	1	1	1	1	1	1	1	1	2	1	1	1	1	2	1	3	1	1	1	1	1	1	1	1	1	1	1	1	1	1
224	Consultant Endocrinologist	THA	6 - 10 years	80-89 patients	2	1	1	1	1	1	2	1	1	1	2	1	1	1	1	1	1	1	2	1	2	2	2	2	2	1	1	3	1	2	1	1	1	1	1	1	1	1	1	1	1	1
225	Consultant Endocrinologist	THA	6 - 10 years	90-99 patients	1	1	1	1	1	1	2	2	1	1	1	1	2	2	1	1	2	1	1	2	2	2	3	2	2	2	1	3	1	1	2	1	1	1	1	2	2	2	1	1	1	1
226	Consultant Nephrologist	EGY	More than 10 years	80-89 patients	1	2	1	2	1	1	2	2	1	1	2	3	3	3	2	1	2	2	2	2	1	2	2	2	3	3	2	1	1	1	2	1	2	2	2	2	1	2	2	2	1	2
227	Consultant Nephrologist	SGP	6 - 10 years	100+ patients	1	1	2	1	1	1	2	1	1	1	1	1	1	2	1	1	1	1	2	1	1	2	2	1	1	1	1	2	2	1	1	1	1	1	1	2	2	2	1	1	1	1
228	Primary Care Physician	THA	More than 10 years	50-59 patients	1	1	1	1	1	1	2	1	1	1	1	1	1	2	1	1	1	1	2	1	2	1	3	2	2	2	1	2	1	1	1	1	1	1	1	1	1	1	1	1	1	1
229	Consultant Cardiologist	THA	More than 10 years	40-49 patients	2	2	1	2	1	1	3	2	2	2	1	2	2	3	1	1	2	2	2	3	2	2	2	2	3	1	2	2	2	2	2	2	1	1	1	2	2	1	2	2	2	1
230	Primary Care Physician	GTM	6 - 10 years	10-19 patients	4	4	3	4	4	4	3	3	2	1	2	2	3	3	1	1	2	2	2	2	2	2	2	2	2	1	1	3	1	2	1	1	1	2	1	1	1	1	1	1	1	1
231	Consultant Endocrinologist	GTM	More than 10 years	10-19 patients	1	1	1	1	1	1	1	1	1	1	1	1	1	2	1	1	1	1	1	1	1	2	1	2	1	1	1	1	1	1	1	1	1	1	1	1	1	1	1	1	1	1
232	Consultant Cardiologist	KOR	More than 10 years	100+ patients	1	2	1	2	1	1	3	2	2	2	1	1	1	1	2	2	2	2	3	3	3	3	2	2	2	1	2	2	2	2	2	1	3	3	2	2	2	2	2	2	1	3
233	Consultant Cardiologist	KOR	More than 10 years	90-99 patients	1	1	1	2	2	2	1	1	1	1	2	1	1	1	1	2	2	3	2	2	2	2	2	2	1	1	1	2	2	2	2	2	3	3	2	1	2	1	1	2	1	1
234	Primary Care Physician	THA	6 - 10 years	100+ patients	1	1	1	2	2	1	2	2	2	1	1	1	1	3	1	1	2	2	1	3	2	2	2	2	2	2	1	1	1	1	1	1	2	2	1	1	1	2	1	1	1	1
235	Consultant Cardiologist	GTM	6 - 10 years	50-59 patients	1	1	1	1	1	1	1	1	1	1	1	1	1	1	1	1	1	1	1	1	1	2	1	1	2	1	1	2	1	1	1	1	1	2	1	1	1	1	1	1	1	1
236	Consultant Cardiologist	THA	6 - 10 years	70-79 patients	1	1	1	2	1	1	1	2	1	1	2	2	1	1	2	1	1	1	1	2	1	1	2	2	1	2	2	3	1	2	2	2	1	2	1	1	2	2	1	1	1	2
237	Primary Care Physician	THA	6 - 10 years	50-59 patients	1	1	1	2	2	1	2	1	1	1	1	1	1	2	2	1	2	2	2	3	2	2	2	2	2	2	1	2	2	2	1	1	2	2	1	2	1	1	1	1	2	1
238	Primary Care Physician	AUS	More than 10 years	100+ patients	2	2	2	2	2	2	2	2	2	2	2	2	2	2	2	2	2	2	2	2	2	3	3	2	3	2	2	1	1	2	1	1	2	3	2	1	1	1	1	1	1	1
239	Primary Care Physician	AUS	6 - 10 years	30-39 patients	1	1	1	1	1	1	1	1	1	1	1	1	1	1	1	1	1	2	2	2	3	2	2	1	1	1	1	1	1	1	1	1	1	1	1	1	1	1	1	1	1	1
240	Primary Care Physician	AUS	More than 10 years	40-49 patients	4	1	1	1	1	1	2	2	2	1	1	2	1	1	1	1	2	2	2	2	2	2	3	2	3	2	1	2	1	1	1	1	1	1	1	1	1	1	1	1	1	1
241	Primary Care Physician	THA	Less than 5 Years	30-39 patients	1	1	2	1	1	1	2	1	2	2	1	1	2	2	2	1	1	2	2	2	2	2	3	2	1	1	1	3	1	1	1	1	1	2	1	1	1	2	1	1	1	1
242	Primary Care Physician	AUS	More than 10 years	80-89 patients	1	1	1	1	1	1	1	1	1	1	1	1	1	1	1	1	1	1	1	1	1	1	1	1	1	1	1	1	1	1	1	1	1	1	1	1	1	1	1	1	1	1
243	Consultant Cardiologist	GTM	More than 10 years	20-29 patients	1	1	1	1	1	1	1	1	1	1	1	1	2	2	1	1	2	1	1	1	1	1	1	1	3	3	1	1	1	1	1	1	1	3	1	1	2	1	1	1	1	1
244	Consultant Cardiologist	GTM	More than 10 years	20-29 patients	1	1	1	1	1	1	1	1	2	1	1	1	1	1	1	1	1	1	1	2	2	1	1	1	2	1	1	1	1	1	1	1	1	1	1	1	1	1	1	1	1	1
245	Consultant Nephrologist	GTM	6 - 10 years	100+ patients	1	1	1	1	1	1	1	1	2	1	1	1	1	1	1	1	1	1	1	1	1	1	1	1	4	1	1	4	1	1	1	1	1	1	1	1	1	1	1	1	1	1
246	Consultant Endocrinologist	GTM	6 - 10 years	30-39 patients	1	1	1	1	1	1	1	1	1	1	1	1	1	1	1	1	1	1	1	1	1	1	1	1	2	1	1	1	1	1	1	1	1	1	1	1	1	1	1	1	1	1
247	Consultant Endocrinologist	GTM	More than 10 years	20-29 patients	1	2	1	2	1	1	1	1	2	1	1	1	1	1	1	1	1	1	2	2	2	1	1	1	2	1	1	1	1	1	1	1	1	2	1	1	1	1	1	1	1	1
248	Consultant Cardiologist	AUS	6 - 10 years	20-29 patients	1	2	2	2	2	2	1	2	2	2	2	2	2	1	2	1	1	2	2	1	2	2	1	2	1	2	1	2	2	1	1	2	2	1	2	2	1	2	2	2	1	2
249	Consultant Endocrinologist	KOR	More than 10 years	60-69 patients	1	1	2	1	1	1	2	1	2	1	1	1	1	1	2	1	1	1	1	1	1	2	2	2	2	1	2	3	2	1	1	2	2	2	1	2	2	2	1	2	2	1
250	Consultant Cardiologist	AUS	More than 10 years	100+ patients	2	2	2	2	2	1	2	1	1	2	2	2	1	2	1	2	2	1	1	1	2	1	2	1	2	1	2	2	2	1	2	1	1	2	2	1	1	2	2	2	1	2
251	Primary Care Physician	EGY	More than 10 years	40-49 patients	1	1	2	2	2	1	2	2	2	2	2	1	1	2	2	1	2	2	2	2	1	2	2	1	1	2	1	1	2	1	2	2	2	2	2	2	1	2	2	1	2	2
252	Consultant Endocrinologist	EGY	More than 10 years	30-39 patients	1	1	2	1	1	1	2	2	1	1	2	1	2	3	2	1	1	2	1	2	2	2	1	2	1	1	1	1	1	1	1	1	2	1	2	1	1	2	1	1	1	1
253	Consultant Endocrinologist	EGY	More than 10 years	50-59 patients	1	1	2	1	1	1	1	1	1	1	1	1	1	2	1	1	1	1	1	1	1	1	1	1	1	1	1	1	1	1	1	1	1	1	1	1	1	1	1	1	1	1
254	Primary Care Physician	EGY	6 - 10 years	80-89 patients	2	1	1	1	1	1	1	2	2	1	2	2	2	2	2	1	1	2	2	2	3	2	2	2	1	1	2	1	1	1	1	1	1	1	1	2	2	1	1	2	2	1
255	Primary Care Physician	EGY	More than 10 years	90-99 patients	1	1	1	1	1	1	2	1	2	2	2	2	1	1	1	2	2	1	2	2	1	1	2	2	2	1	1	1	2	1	2	2	2	1	1	2	2	1	1	1	2	1
256	Primary Care Physician	EGY	Less than 5 Years	70-79 patients	2	1	2	1	1	2	1	1	1	2	1	2	1	1	2	1	2	1	2	2	2	1	2	2	1	1	1	2	1	1	2	2	1	2	1	1	2	1	2	1	1	2
257	Primary Care Physician	EGY	6 - 10 years	80-89 patients	2	1	2	2	1	1	2	2	1	1	2	2	1	2	2	2	1	1	1	2	1	2	1	1	1	2	2	2	1	1	1	2	2	1	1	2	1	2	1	2	1	1
258	Consultant Endocrinologist	EGY	6 - 10 years	100+ patients	3	1	1	1	1	1	1	2	1	1	2	1	1	2	1	1	2	2	2	2	3	2	1	2	2	3	1	2	2	3	3	2	2	1	1	1	1	2	1	1	1	1
259	Consultant Nephrologist	EGY	More than 10 years	90-99 patients	1	2	2	2	2	1	2	2	3	2	2	2	2	2	1	2	2	2	3	3	2	3	3	2	2	2	1	3	2	2	2	2	2	2	3	2	2	2	2	2	2	2
260	Consultant Nephrologist	EGY	6 - 10 years	80-89 patients	1	2	2	1	1	1	1	2	1	1	1	1	1	1	1	1	1	2	2	2	2	1	2	2	3	1	1	1	1	1	1	1	1	1	1	1	2	2	2	2	2	2
261	Primary Care Physician	EGY	Less than 5 Years	70-79 patients	1	2	2	2	2	1	2	2	3	2	2	2	2	2	1	2	2	2	3	3	2	3	3	2	2	2	1	3	2	2	2	2	2	2	3	2	2	2	2	2	2	2
262	Consultant Endocrinologist	EGY	More than 10 years	80-89 patients	2	2	1	2	2	1	1	2	1	1	2	1	1	2	2	1	2	2	3	3	2	3	3	2	2	2	1	3	2	2	2	2	2	2	3	1	2	2	1	2	2	2
263	Consultant Endocrinologist	MEX	More than 10 years	10-19 patients	1	3	1	1	1	1	1	1	2	1	1	2	2	2	1	1	1	1	1	1	1	3	3	3	3	2	1	3	1	3	3	1	2	3	1	1	2	1	2	1	1	2
264	Consultant Nephrologist	BRA	6 - 10 years	60-69 patients	1	1	2	1	1	1	2	1	2	1	2	1	2	2	1	1	2	2	2	2	3	2	2	2	2	2	1	1	1	1	2	2	1	2	1	1	1	2	2	1	1	1
265	Consultant Nephrologist	BRA	6 - 10 years	60-69 patients	1	2	1	1	1	1	1	2	2	1	1	2	1	2	1	1	1	1	2	1	1	1	1	2	2	2	1	1	1	1	1	1	1	1	1	1	2	2	1	1	1	1
266	Consultant Nephrologist	EGY	More than 10 years	100+ patients	1	2	1	2	2	1	1	2	3	2	2	2	2	2	1	2	2	1	3	3	2	3	3	2	2	2	1	3	2	2	2	2	2	2	3	1	1	2	2	2	1	2
267	Primary Care Physician	EGY	Less than 5 Years	70-79 patients	1	1	2	1	1	1	1	1	2	2	1	1	2	2	1	1	1	2	2	2	1	2	1	2	2	2	1	2	2	1	1	1	1	1	1	1	1	1	1	1	1	1
268	Primary Care Physician	EGY	6 - 10 years	80-89 patients	1	1	1	1	2	1	1	1	1	1	2	1	1	2	2	1	1	1	1	2	1	2	1	3	2	2	1	1	1	1	1	1	1	1	1	2	1	1	1	1	1	1
269	Primary Care Physician	EGY	Less than 5 Years	70-79 patients	1	1	1	1	2	3	2	1	1	1	2	2	3	2	2	1	1	1	1	1	2	1	1	2	1	1	1	2	1	1	1	1	1	1	1	1	1	1	1	1	1	1
270	Primary Care Physician	EGY	Less than 5 Years	80-89 patients	1	1	1	1	1	1	2	1	1	1	2	1	2	1	1	1	1	2	2	1	2	2	2	1	2	1	1	1	1	1	1	1	1	1	1	2	1	1	1	1	1	1
271	Primary Care Physician	EGY	6 - 10 years	70-79 patients	1	1	1	1	1	1	2	1	1	2	1	2	2	1	1	1	1	2	1	1	2	2	1	2	2	1	1	1	1	1	1	1	1	1	1	1	1	1	1	1	1	1
272	Consultant Endocrinologist	EGY	More than 10 years	70-79 patients	1	1	1	1	1	1	2	2	1	1	1	1	1	1	1	1	1	2	2	2	2	1	2	2	3	1	1	1	1	1	1	1	1	1	1	1	2	2	2	2	2	2
273	Consultant Cardiologist	EGY	6 - 10 years	40-49 patients	1	1	1	1	1	2	2	2	1	1	2	1	1	1	1	1	1	2	2	2	2	2	2	1	2	2	1	1	1	1	1	1	1	1	1	1	1	1	1	1	1	1
274	Consultant Cardiologist	EGY	6 - 10 years	50-59 patients	1	1	1	1	1	1	2	2	1	1	2	2	1	1	1	1	1	2	2	2	2	2	1	1	2	2	1	1	1	1	1	1	1	1	1	1	1	1	1	1	1	1

Key
1 - Strongly Agree, 2 - Tend to Agree, 3 - Tend to Disagree, 4 - Strongly Disagree

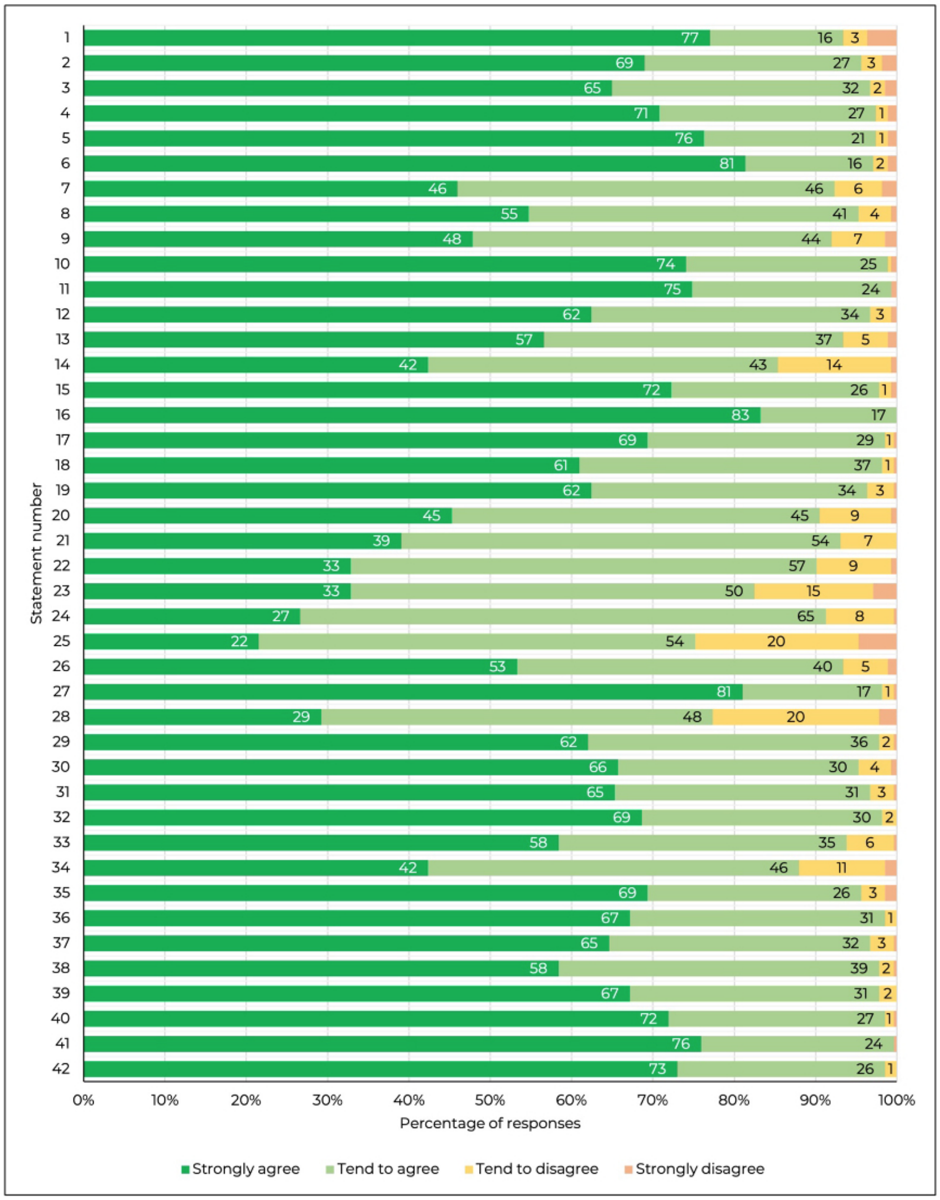

306x390mm (72 x 72 DPI)

CREDES Checklist:

Recommendations for the Conducting and REporting of DElphi Studies (CREDES)[1]

Results from a cross-specialty consensus on optimal management of the chronic kidney disease (CKD) patient: from screening to complications

Items of reporting	Reported on page
Purpose and rationale. The purpose of the study should be clearly defined and demonstrate the appropriateness of the use of the Delphi technique as a method to achieve the research aim. A rationale for the choice of the Delphi technique as the most suitable method needs to be provided.	5 - 6
Expert panel. Criteria for the selection of experts and transparent information on recruitment of the expert panel, sociodemographic details including information on expertise regarding the topic in question, (non)response and response rates over the ongoing iterations should be reported.	5 - 6
Description of the methods. The methods employed need to be comprehensible; this includes information on preparatory steps (How was available evidence on the topic in question synthesised?), piloting of material and survey instruments, design of the survey instrument(s), the number and design of survey rounds, methods of data analysis, processing and synthesis of experts' responses to inform the subsequent survey round and methodological decisions taken by the research team throughout the process.	5 - 7
Procedure. Flow chart to illustrate the stages of the Delphi process, including a preparatory phase, the actual 'Delphi rounds', interim steps of data processing and analysis, and concluding steps.	Figure 1
Definition and attainment of consensus. It needs to be comprehensible to the reader how consensus was achieved throughout the process, including strategies to deal with non-consensus.	7
Results. Reporting of results for each round separately is highly advisable in order to make the evolving of consensus over the rounds transparent. This includes figures showing the average group response, changes between rounds, as well as any modifications of the survey instrument such as deletion, addition or modification of survey items based on previous rounds.	8 - 9
Discussion of limitations. Reporting should include a critical reflection of potential limitations and their impact of the resulting guidance.	14 - 15
Adequacy of conclusions. The conclusions should adequately reflect the outcomes of the Delphi study with a view to the scope and applicability of the resulting practice guidance.	10 - 16
Publication and dissemination. The resulting guidance should be clearly identifiable from the publication, including recommendations for transfer into practice and implementation.	15 - 16

1. Jünger S, Payne SA, Brine J, Radbruch L, Brearley SG. Guidance on Conducting and REporting DElphi Studies (CREDES) in palliative care: Recommendations based on a methodological systematic review. Palliat Med. 2017;31: 684–706. doi:10.1177/0269216317690685

For peer review only

BMJ Open

Results from a cross-specialty consensus on optimal management of the chronic kidney disease (CKD) patient: from screening to complications

Journal:	*BMJ Open*
Manuscript ID	bmjopen-2023-080891.R2
Article Type:	Original research
Date Submitted by the Author:	12-Feb-2024
Complete List of Authors:	Arici, Mustafa ; Hacettepe Universitesi, ; Hacettepe University Faculty of Medicine<br>Assaad – Khalil, Samir; Alexandria University Faculty of Medicine, Unit of Diabetes, Lipidology & Metabolism<br>Bertoluci, Marcello; Universidade Federal do Rio Grande do Sul Instituto de Biociencias, Endocrinology Division, Hospital de Clínicas de Porto Alegre<br>Choo, Jason; Singapore General Hospital, Department of Renal Medicine<br>Lee, Yau-Jiunn; Lee's Clinic<br>Madero, Magdalena; National Heart Institute of Mexico<br>Rosa Diez, Guillermo ; Hospital Italiano de Buenos Aires, Servicio de Nefrología<br>Sánchez Polo, Vicente ; Instituto Guatemalteco de Seguridad Social, Servicio de Nefrología y Trasplante Renal<br>Chung, Sungjin; The Catholic University of Korea College of Medicine, Division of Nephrology<br>Thanachayanont, Teerawat; Bhumirajanagarindra Kidney Institute, Nephrology<br>Pollock, Carol; University of Sydney
**Primary Subject Heading**:	Renal medicine
Secondary Subject Heading:	Cardiovascular medicine, General practice / Family practice, Global health
Keywords:	Nephrology < INTERNAL MEDICINE, Heart failure < CARDIOLOGY, Hypertension < CARDIOLOGY, Chronic renal failure < NEPHROLOGY, Primary Care < Primary Health Care, Surveys and Questionnaires

SCHOLARONE™
Manuscripts

I, the Submitting Author has the right to grant and does grant on behalf of all authors of the Work (as defined in the below author licence), an exclusive licence and/or a non-exclusive licence for contributions from authors who are: i) UK Crown employees; ii) where BMJ has agreed a CC-BY licence shall apply, and/or iii) in accordance with the terms applicable for US Federal Government officers or employees acting as part of their official duties; on a worldwide, perpetual, irrevocable, royalty-free basis to BMJ Publishing Group Ltd ("BMJ") its licensees and where the relevant Journal is co-owned by BMJ to the co-owners of the Journal, to publish the Work in this journal and any other BMJ products and to exploit all rights, as set out in our licence.

The Submitting Author accepts and understands that any supply made under these terms is made by BMJ to the Submitting Author unless you are acting as an employee on behalf of your employer or a postgraduate student of an affiliated institution which is paying any applicable article publishing charge ("APC") for Open Access articles. Where the Submitting Author wishes to make the Work available on an Open Access basis (and intends to pay the relevant APC), the terms of reuse of such Open Access shall be governed by a Creative Commons licence – details of these licences and which Creative Commons licence will apply to this Work are set out in our licence referred to above.

Other than as permitted in any relevant BMJ Author's Self Archiving Policies, I confirm this Work has not been accepted for publication elsewhere, is not being considered for publication elsewhere and does not duplicate material already published. I confirm all authors consent to publication of this Work and authorise the granting of this licence.

For peer review only

Results from a cross-specialty consensus on optimal management of the chronic kidney disease (CKD) patient: from screening to complications

Arici M M.D.[1], Assaad-Khalil S M.D.[2], Bertoluci MC M.D.[3], Choo JCJ M.B.B.S.[4], Lee Y-J M.D.[5], Madero M M.D.[6], Rosa-Diez G M.D.[7], Sánchez-Polo V M.D.[8], Chung S M.D.[9], Thanachayanont T M.D.[10], Pollock C M.B.B.S.[11]

1. Division of Nephrology, Department of Internal Medicine, Faculty of Medicine, Hacettepe University, Ankara, Türkiye
2. Unit of Diabetes, Lipidology & Metabolism; Department of Internal Medicine/ Alexandria Faculty of Medicine, Alexandria University, Alexandria, Egypt
3. Endocrinology Division, Hospital de Clínicas de Porto Alegre, Universidade Federal do Rio Grande do Sul, Porto Alegre, Brazil
4. Department of Renal Medicine, Singapore General Hospital, Singapore
5. Lee's Endocrinology Clinic, Pingtung 90000, Taiwan
6. Division of Nephrology, Department of Medicine, National Heart Institute, Mexico City, Mexico
7. Servicio de Nefrología, Hospital Italiano de Buenos Aires, Buenos Aires, Argentina
8. Nephrology Department, Guatemalan Social Security Institute (IGSS)
9. Department of Internal Medicine, College of Medicine, The Catholic University of Korea, Seoul, Republic of Korea; Clinical Practice Guideline Committee, The Korean Society of Nephrology
10. Bhumirajanagarindra Kidney Institute, Bangkok Thailand
11. Kolling Institute of Medical Research, Sydney Medical School, University of Sydney, Royal North Shore Hospital, St Leonards, NSW, Australia

Correspondence to:

Professor M Arici
Division of Nephrology, Department of Internal Medicine, Faculty of Medicine,
Hacettepe University, Istanbul, Turkey
Division of Nephrology, Department of Internal Medicine,
Faculty of Medicine, Hacettepe University,
Ankara, Türkiye
marici@hacettepe.edu.tr

Main text = 3,833 words

For peer review only

KEYWORDS

- Chronic Kidney Disease
- Heart Failure
- Hypertension
- Hyperkalaemia
- Primary Care
- Multidisciplinary Care Team

ABSTRACT (300 WORDS)

Background: Chronic kidney disease (CKD) affects around 10% of the global population and has been estimated to affect around 50% of individuals with type 2 diabetes and 50% of those with heart failure. The guideline recommended approach is to manage with disease-modifying therapies, but real-world data suggests that prescribing rates do not reflect this in practice.

Objective: To develop a cross-specialty consensus on optimal management of the CKD patient using a modified Delphi method.

Design: An international steering group of experts specialising in internal medicine, endocrinology/diabetology, nephrology and primary care medicine developed 42 statements on aspects of CKD management including identification and screening, risk factors, holistic management, guidelines, cross-specialty alignment, and education. Consensus was determined by agreement using an online survey.

Participants: The survey was distributed to cardiologists, nephrologists, endocrinologists, and primary care physicians across 11 countries.

Main outcomes and measures: The threshold for consensus agreement was established *a priori* by the steering group at 75%. Stopping criteria were defined as a target of 25 responses from each country (N=275), and a four-week survey period.

Results: 274 responses were received in December 2022, 25 responses from Argentina, Australia, Brazil, Guatemala, Mexico, Singapore, South Korea, Taiwan, Thailand, Turkey, and 24 responses from Egypt. 53 responses were received from cardiologists, 52 from nephrologists, 55 from endocrinologists, and 114 from primary care physicians. 37 statements attained very high agreement (≥90%) and 5 attained high agreement (≥75% and

For peer review only

<90%). Strong alignment between roles was seen across the statements, and different levels of experience (2-5 years or 5+ years), some variation was observed between countries.

Conclusions: There is a high degree of consensus regarding aspects of CKD management among healthcare professionals from 11 countries. Based on these strong levels of agreement, the steering group derived twelve key recommendations focused on diagnosis and management of CKD.

STRENGTHS AND LIMITATIONS OF THIS STUDY

- 274 responses received with good representation from experienced physicians across 11 different countries
- Majority of responses from physician with more than ten years' experience in role, suggesting that the results represent the views of experienced physicians.
- Very high levels of agreement may suggest either that the statements were constructed to achieve agreement (confirmation bias), or that they represent already recognised good practice.
- Lack of representation from Low- or Middle-Income Countries (LMICs), therefore these findings may be of limited applicability to LMICs and a further Delphi process would be required to capture this perspective.
- Aspects of patient choice and empowerment and consideration of the patient experience (outside of treatment outcomes) have not been discussed.

For peer review only

BACKGROUND

Chronic kidney disease (CKD) is defined as abnormalities of kidney structure or function, which are present for >3 months.[1] This is indicated by a glomerular filtration rate (GFR) below 60 mL/min/1.73 m², or the presence of one or more markers of kidney damage. CKD is classified based on cause, glomerular filtration rate category, and albuminuria category.[1] Diabetes is the leading cause of CKD, followed by hypertension, suggesting a close relationship between the cardio-renal-metabolic systems.[2] The interplay between CKD, cardiovascular disease (CVD), and metabolic diseases such as type 2 diabetes mellitus (T2DM) is significant and has been termed 'cardio-renal-metabolic' (CRM) disease.[3] It has been suggested that CKD affects around 50% of people with T2DM [4], around 50% of heart failure (HF) patients [5], and up to 38% of those with hypertension [6], suggesting the need for a holistic approach to management of CKD patients.

Between 1990 and 2017, the prevalence of CKD was estimated to rise to 9.1% of the global population, with associated mortality increasing by 41.5%.[7] This is closely associated with an increase in populations with risk factors such as diabetes, hypertension, and prediabetes.[2] As a consequence of these factors, 1·2 million people dies in 2017 from CKD.

Early diagnosis and referral of CKD are key to reducing or avoiding progression to kidney failure, and reducing morbidity and mortality. The absence of symptoms in early stages of CKD requires that clinicians maintain suspicion in all patients and especially those with risk-factors.[8] Accurate screening, diagnosis, and risk stratification need to be in place to support the aim of 'earliest diagnosis'.

A diagnosis of CKD is determined by laboratory confirmation of proteinuria or haematuria, and/or a reduction in the glomerular filtration rate (GFR), for more than 3 months' duration.[9] Two key markers of CKD include albuminuria (defined as a urinary albumin-to-creatine ratio [uACR] of $\geq$30 mg/g) and reduction in estimated GFR (eGFR), as calculated by the CKD-EPI based on creatinine alone ($eGFR_{cr}$) or on creatinine and cystatin C ($eGFR_{cr-cys}$).[9,10] In practice, these methods may not be utilised effectively, an analysis of over 123,000 patient records found low frequency of uACR testing in patients with CKD, despite strong epidemiological evidence linking increased albuminuria with disease progression, kidney failure, cardiovascular events, and premature mortality.[11]

Besides lifestyle modifications, disease-modifying therapies (DMTs) for CKD including renin-angiotensin-aldosterone system inhibitors (RAASi), and sodium-glucose co-transporter-2 inhibitors (SGLT2i), as recommended by Kidney Disease Improving Global Outcomes (KDIGO) for use in CKD patients with hypertension or diabetes,

For peer review only

and non-steroidal mineralocorticoid antagonists (ns-MRAs) as add-on therapy in patients with T2DM and residual albuminuria.[1,12] Despite the availability of these therapies, analysis of 2 large US healthcare systems showed that even though nearly two-thirds of the adults with CKD had diabetes, hypertension, or prediabetes, rates of prescribing RAASi were low. Not only was DMT use below guideline directed levels, but potentially nephrotoxic agents (such as non-steroidal anti-inflammatory drugs and proton pump inhibitors) were used more commonly than RAASi.[2]

There is clearly some disconnect between guideline recommendations and real-world practice. Guidelines for the management of CKD may not be clear or made known to primary care practitioners (PCPs). The objective of this project is to use a modified Delphi technique to examine the opinions of healthcare professionals towards aspects of CKD management across 11 countries, report these findings, and develop practical recommendations for diagnosis and management of CKD.

METHODS

A steering group of experts (see author list) from 11 different countries convened in September 2022 to discuss current challenges in CKD management. The experts were defined as specialists in nephrology, endocrinology/diabetology, internal medicine, and primary care medicine who had achieved an appropriate level of seniority within their field (e.g., professors, clinical directors), or had published papers related to the management of CKD/HF/Diabetes, or had been involved in guidelines development.[13-21] The steering group members were selected to provide representation across a range of development indicator levels external to Europe and North America (to avoid replication of previous published work [22]). Steering Group members were recruited to represent countries outside of North America and Europe. Central and South America, Southeast Asia, Middle East and Africa were initially targeted. The process of recruitment involved identification of potential group members from each of these regions using desk research, followed by a snowball method until all target regions had at least one representative on the Steering Group. Although a wide representation was aimed for, it is a limitation that there were not enough members from low-income countries.

The Delphi technique used in this study was guided by Guidance on Conducting and REporting DElphi Studies (CREDES). Whilst guidelines exist, outcomes for CKD vary between countries, to understand the barriers and opportunities, a modified Delphi approach was employed to understand where common care processes differ in local use, and how the attitudes of healthcare professionals to elements of CKD management differ between countries. The overall process is outlined in Figure 1.

For peer review only - http://bmjopen.bmj.com/site/about/guidelines.xhtml

Figure 1: Study flow diagram

<<Figure 1>>

Six themes for focus for statement development were agreed (Table 1), these were discussed further, and statements developed by the Steering Group working collaboratively. The statements were then collated, and the steering group independently rated the statements as either “accept”, “remove”, or “reword” with suggested changes (as determined by a simple majority) with the potential for further group consultation for any significant differences of opinion on the fundamental principles of any statement. Once finalised, the steering group were agreed the final set of statements for testing. This constituted the initial round of consensus.

The resulting 42 statements were developed into a *Likert* survey, this was then distributed by a third party (Sermo) in Round 2 of the process.

Panel members were recruited based on the following criteria:

- Employed within one of the 11 target countries.
- 25 respondents from each country in a broadly 2:1:1:1 ratio of Primary Care Physician, Consultant Endocrinologist, Consultant Cardiologist, Consultant Nephrologist (or local equivalent).

The identity of respondents was not known to the steering group or the independent facilitator. For each statement, respondents were offered a 4-point scale (‘strongly disagree’, ‘tend to disagree’, ‘tend to agree’, and ‘strongly agree’) to indicate their level of agreement with each statement. The survey also captured country, specialty, length of time in role, and average number of CKD patients managed over 3 months. Stopping criteria for data collection were defined as a target of 25 responses from each country (N=275), and a four-week survey period.

The target countries were chosen to reflect the Steering Group demographic, this would enable each Steering Group member to provide insight into results from their respective country. Panel members were recruited from the following countries: Argentina (UMI), Australia (HI), Brazil (UMI), Egypt (LMI), Guatemala (UMI), Mexico (UMI), Singapore (HI), South Korea (HI), Taiwan (HI), Thailand (UMI), and Turkey (UMI)[i].

[i] HI – High Income, LMI – Lower-Middle Income, UMI – Upper-Middle Income, according to World Bank Classification.

For peer review only

The closing criteria for the study was defined *a priori* in line with best practice principles as: 90% of the final statement set achieving consensus threshold (defined at 75%, a widely accepted threshold).[23] If these criteria were not met, statements would be modified, and the survey re-issued as necessary for a maximum of three rounds. Consensus was further categorised as 'high' at ≥75% and 'very high' at ≥90%. Completed surveys were analysed using Microsoft Excel software. The responses were aggregated to provide an overall agreement level (i.e., the number of responded expressing agreement as a percentage of the overall number of responses for each statement).

As this study only sought the anonymous opinions of physicians and no patient-specific data were captured, ethical approval was not sought. Respondents provided consent to have their responses included in this study through their inclusion to the panel held by Sermo.

Patient and Public Involvement

None, the stated objective was to examine the opinions of healthcare professionals towards aspects of CKD management across 11 countries.

Results

Of the 45 initial statements created by the Steering Group, 32 were retained, 8 were modified, 5 were removed, and 3 new statements were added. The final set was then sent out to the group via email for acceptance prior to progressing to Round 2.

In Round 2, completed surveys from a panel of 274 were analysed to define the total level of agreement with each of the 42 statements. Respondents were either PCPs or consultants in cardiology, nephrology, and endocrinology (including diabetology) (Figure 2). Most responders had more than 10-years' experience in role (159/274) and only 13% (n=35) had less then 5 years in role. When asked to estimate the number of patients seen in a 3-month period, 177 responders (65%) stated that they saw more than 50 patients (either as inpatients or outpatients). This suggests the respondent cohort was sufficiently experienced to provide insight into aspects of CKD.

Figure 2: Respondents by role

<<Figure 2>>

For peer review only

Consensus was achieved for all statements, with very high agreement (≥90%) in 37 (88%) statements, and high agreement (≥75 & ≤89%) in 5 (12%) statements. Overall consensus agreement by statement is shown in Figure 3, and Table 1, a detailed results showing percentages of agreement at each level can be found in supplementary Table S1 and Figure S1.

Figure 3: Overall consensus agreement levels by statement[a]

<<Figure 3>>

[a]The green horizontal line represents the 75% threshold for consensus agreement and the blue line indicates the threshold for very high consensus (90%).

Table 1. Agreement to each statement, divided by topic.

No:	Statement:	Overall
**Earlier identification & screening of CKD**		
**1.**	Late diagnosis is a lost opportunity for early management of CKD which can slow disease progression	**93%**
**2.**	National screening and diagnostic programmes for CKD are essential to identifying patients at earlier stages of CKD	**96%**
**3.**	A simple and practical definition of 'high-risk' should be established to support cost-effective screening	**97%**
**4.**	Early screening for CKD in high-risk groups is cost-effective for the health system (where resources are in place to support intervention)	**97%**
**5.**	Primary care physicians and specialists (cardiologists, endocrinologists, nephrologists, diabetologists, etc.) should routinely screen for CKD in patients with risk factors	**97%**
**6.**	Patients with risk factors (e.g., elderly, diabetes, hypertension, dyslipidaemia, obesity, family history of CKD) should be screened at least annually for CKD	**97%**
**7.**	Patients with CKD should be screened for heart failure (including those with preserved ejection fraction (HFpEF))	**92%**
**8.**	GFR estimated by the CKD-EPI Creatinine Equation (2021) and albuminuria (using albumin-to-creatinine ratio) should be the screening method of choice for CKD	**95%**
**9.**	Confirmatory testing of abnormalities of kidney function or structure at 3 months is required to establish a CKD diagnosis	**92%**
**Risk factors for CKD in cardio-renal-metabolic patients**		
**10.**	A holistic view of cardio-renal-metabolic disease states is critical to provide integrated patient-centred care to individuals with CKD	**99%**
**11.**	CKD progressively increases the risk of CVD, including heart failure and overall mortality	**99%**
**12.**	Frequency of hyperkalaemia increases as CKD progresses	**97%**
**13.**	AKI is a risk factor for CKD and chronic heart failure	**93%**
**14.**	Infection, including COVID-19, can cause AKI and is therefore a risk factor for CKD	**85%**
**15.**	Nephrotoxic medications should be modified, and risk/benefit profiles should be assessed in patients with, or at risk of CKD	**98%**
**Holistic management of CKD in cardio-renal-metabolic patients**		
**16.**	Lifestyle factors such as diet, smoking, exercise must be optimised for CKD patients	**100%**
**17.**	Disease modifying therapies for CKD (e.g., SGLT2i, RAASi, MRA) should be used where indicated to manage progression of CKD	**99%**
**18.**	SGLT2i and RAASi have a complementary cardio-renal protective action	**98%**
**19.**	Early use of SGLT2i could prevent the development and progression of CKD patients with type 2 diabetes and heart failure	**96%**
**20.**	Early use of SGLT2i can slow progression of CKD in patients without diabetes	**91%**

For peer review only - http://bmjopen.bmj.com/site/about/guidelines.xhtml

**21.**	A small decrease of eGFR (~10%) may be expected on initiation of CKD disease-modifying therapies	**93%**
**22.**	De-escalation or discontinuation of RAASi therapy is associated with worse cardiovascular and renal outcomes in the CKD patient	**90%**
**23.**	Disease modifying therapies for CKD should only be stopped as a last resort	**82%**
**24.**	Novel K binders (i.e., patiromer and sodium zirconium cyclosilicate) are an option to manage hyperkalaemia and prevent de-escalation or downtitration of RAASi	**91%**
**25.**	Action to manage hyperkalaemia in the CKD patient should be taken when serum potassium level reaches 5.0mmol/L	**75%**
**26.**	Action to manage hyperkalaemia in the CKD patient should be taken when serum potassium level reaches 5.5 mmol/L	**93%**
**27.**	Treatment targets such as blood pressure, blood glucose, lipid levels should be maintained for both kidney and cardiac outcomes in CKD patients	**98%**
**Guidelines**		
**28.**	Implementation of evidence-based CKD guidelines is suboptimal and should be improved	**77%**
**29.**	Cardiology, nephrology, and endocrinology guidelines for CKD should be aligned	**98%**
**30.**	Primary care physicians have an important role in implementing guidelines	**95%**
**31.**	Guidelines should be practical with an executive summary / checklist to assist implementation by non-specialist HCPs	**97%**
**32.**	Guidelines should include clear criteria for when and how to refer to other specialists/MDT	**98%**
**33.**	Guidelines should reflect differences in population phenotypes and be locally adapted where needed	**94%**
**34.**	Patient perspective is crucial in developing guidelines	**88%**
**35.**	Investment is needed to shift the approach to primary prevention rather than end stage kidney disease management	**96%**
**Cross-specialty alignment (Cardiology, Nephrology, Endocrinology, Primary Care & Policy Makers)**		
**36.**	Patients with CKD should be managed by a multi-disciplinary team (MDT) where possible with an agreed management plan	**99%**
**37.**	The CKD multi-disciplinary team (MDT) should include the primary care physician to improve early intervention and decision making	**97%**
**38.**	The care pathway for CKD should be designed to minimise the impact of organisational barriers	**98%**
**39.**	Clinicians, professional associations, academic institutions, and patient representative organisations need to engage with policy makers to ensure appropriate plans and funding are in place to deliver optimal CKD care	**98%**
**Education of clinicians and patients**		
**40.**	Up-to-date healthcare professional (HCP) education on the management of patients with CKD and associated guidelines is needed	**99%**
**41.**	Structured education of primary care physicians on screening and early detection improves patient outcomes	**100%**
**42.**	Therapeutic patient education is key for them to understand the consequences of CKD and how to manage it through lifestyle modification and appropriate use of therapies	**99%**

As none of the statements failed to reach the predetermined threshold of 75%, only one round of survey was required. The results of the survey represent current opinions of the respondents and are not intended to contradict the established evidence base. Anonymized Round 2 results data are available in supplemental Table S2.

DISCUSSION (Note, in the discussion below, 'S' is used to denote 'statement'.)

Earlier identification & screening of CKD (S1-9)

Very high agreement (>90%) was observed for all statements in this topic, underscoring the key principle that early diagnosis of CKD is key to implementing strategies to slow disease progression. Responses suggest a strong

For peer review only

support for the need for national kidney health screening and diagnostic programmes. Universal screening of the general population has been found not to be cost-effective [24], but systematic review has shown screening to be cost-effective in patients with hypertension or diabetes.[25] Indeed, a KDIGO Controversies Conference concluded that targeted groups, such as those with hypertension, diabetes, or cardiovascular disease should be screened for CKD, and that an individualised approach should be taken to screen others based on a range of factors.[26]

Respondents agree that patients with risk factors should be screened for CKD at least annually by using eGFR and uACR where available, but identification of CKD is also challenging where awareness amongst healthcare staff and health literacy in the general population is poor, it is therefore recommended that national initiatives to improve these issues should support any screening programme.

A minimal-resource pre-screening tool has been developed and globally validated for CKD in people with T2DM. This demonstrates that age, gender, body mass index, duration of diabetes, and blood pressure information can be used to identify those at an increased risk of CKD.[27] This model does not require sophisticated diagnostics and can be used to guide cost-effective screening for CKD where resources are limited.

Risk factors for CKD in cardio-renal-metabolic patients (S10-15)

All statements in this topic achieved >90% consensus apart from statement 14 (85%). This is interesting and could be due to the wording of the statement and the specific use of COVID-19 as an example. Reference to COVID-19 was included in the statement as it was considered relevant by the steering group at the time of statement generation and evidence was beginning to emerge of potential kidney injury associated with COVID-19. Analysis by country found that Taiwan agreement was at 68% for this statement, noticeably lower than for other countries. Although 93% of respondents agree that AKI is a risk-factor for CKD and HF, it is interesting that 7% do not agree given the evidence base.[28,29]

Given the established interplay between the cardiac, renal and endocrine systems, it is heartening to see that 99% of respondents agree that a holistic approach is needed to provide personalised care for individuals with CKD.

Where practical, models of care should be developed to deliver integrated multidisciplinary care to patients with cardio-renal-metabolic disease, as described by the CardioMetabolic Care Alliance, to use comprehensive, patient-centred, team-based approaches for aggressive secondary prevention. [30] Implementing combined clinics to deliver medical care for patients with kidney disease and diabetes or CVD may reduce outpatient healthcare costs

For peer review only - http://bmjopen.bmj.com/site/about/guidelines.xhtml

without compromising health outcomes.[31] Combined multidisciplinary clinics for diabetes, CKD, and CVD are also associated with a slower decline in GFR than usual care, and a significant reduction in the risk for all-cause mortality.[32] However, global variation exists regarding resource availability and access to specialist care [33], and the use of MDTs is not universal. In these regions, HCPs are encouraged to develop local contacts/networks to allow for inter-disciplinary discussions of CKD cases where needed.

Holistic management of CKD in cardio-renal-metabolic patients (S16-27)

In the broad objective of this consensus is to promote the earlier detection and management of CKD, and this principle applies regardless of resource limitations. As CKD associated health care costs increase with disease progression [34], the economic argument supports this approach, and in patients with limited access to specialist care (including DMT), the role of patient education is paramount in improving health literacy and promoting lifestyle changes to reduce CKD risk. Coupled with this, the health system should look at how interventions such as DMTs can be used to slow progression of CKD and the need for dialysis (and associated costs), cost-savings from slower progression to dialysis dependence might be reinvested in detection and diagnosis/national screening of high-risk patient groups.

Very high agreement was observed for 10 of the statements in this topic, with two statements at lower levels of agreement (S23 and S25, 82% and 75%, respectively). Response to statement 23 is interesting, given the current debate regarding the benefits of stopping RAASi in advanced CKD. Results from a 52-week open-label UK study in patients with CKD stages 4-5 concluded 'The STOP-ACEi trial did not find any benefit by stopping RAASi in advanced CKD', and that 'stopping RAASi in advanced CKD at an arbitrary GFR threshold is not the optimal approach'. Large observational studies have confirmed the cardioprotective benefits of RAASi in advanced CKD (including patients with concomitant T2DM).[35-37] Linked to this is statement 21, respondents agree that a small decrease in eGFR is to be expected upon initiation of DMT for CKD. During initiation and up-titration of RAASi treatment, a decline in kidney function of up to 30% within 4 weeks can be acceptable [1,38] and it is important to avoid a knee-jerk response and reduce or stop DMT, and consultation with a Nephrologist or multidisciplinary team (MDT) is recommended.

Hyperkalaemia (HK) is potentially life-threatening; it may be acute or chronic and individuals with CKD are at an increased risk, which increases with the later stages of CKD.[39] HK treatment is often stratified by serum potassium level, with borderline levels warranting modification of dietary potassium intake, followed by pharmaceutical management for larger or more sustained increases.[40] Evidence to support recommending low

For peer review only

potassium diets to patients with advanced CKD or ESRD is weak. Observational studies report weak associations between dietary potassium intake and potassium concentration and this approach may deprive patients of the beneficial cardiovascular effects associated with potassium-rich diets.[41]

When HK occurs, RAASi dose reduction or discontinuation are the most common utilized therapeutic options, but this approach is associated with worse cardiorenal outcomes and increased mortality.[42-44] Once stopped, RAASi treatment is rarely reinitiated.[45] In cases of mild-to-moderate hyperkalaemia, DMT should be maintained where possible [1,14], and an option for achieving this is using a potassium lowering therapy such as patiromer or sodium zirconium cyclosilicate (S24, 91%).

Whilst the threshold for intervention in HK may vary by country and even individual physician, we can conclude that action may be considered when K^+ concentration reaches 5.0 mmol/L (S25, 75%) and certainly when at 5.5 mmol/L (S26, 93%) but with a degree of personalisation (in consultation with a nephrologist). Statements 25 and 26 were included to try and understand where the weight of opinion fell regarding interventions to manage hyperkalaemia, both statements achieved consensus, but the strongest agreement was for statement 26. On reflection, these statements could have been worded better to understand what would constitute appropriate action in these circumstances.

Respondents very strongly agree that SGLT2i and RAASi therapies have a complementary cardio-renal protective action (S18, 98%) and that in patients with T2DM and HF, early SGLT2i use can prevent the development and progression of CKD (S19, 96%), in fact, SGLT2i may slow progression of CKD in patients without T2DM (S20, 91%). It is therefore recommended that SGLT2i and RAASi therapies are initiated as early as possible to both delay CKD progression and to therapeutically address the full cardio-renal-metabolic continuum – including T2DM and HF.

Guidelines (S28-35)

The interplay between CKD, diabetes, and CVD have been discussed, but CKD is associated with a number of comorbidities. A prospective UK cohort study of 1,741 people with CKD stage 3 found that isolated CKD (without comorbidities) was present in only 4% of patients, and that common comorbidities include ‘painful condition’ (30%), anaemia (24%), thyroid disorders (12%), cerebrovascular disease (12%), and respiratory conditions (10%). These comorbidities should be identified and managed as part of a multidisciplinary approach.[46]

For peer review only

Whilst all statements in this topic achieved consensus threshold, the steering group were surprised that 23% of respondents did not agree that implementation of CKD guidelines is suboptimal. The lowest agreement levels were observed in South Korea (68%) and Guatemala (72%). This could be either genuine strength in implementation of guidelines in these countries (which should be investigated and replicated elsewhere), or the need for continuing professional development (CPD) even amongst experienced physicians.

Due to the vital role of primary care must play in identifying and referring suspected CKD patients, respondents agree that clear guidelines for referral of patients are considered essential (S32, 98%). PCPs should be provided with clear criteria for referral and followed through with continuing education from an appropriate specialist (ideally a nephrologist). The focus on prevention can only be achieved through a whole-system approach with appropriate investment in awareness, screening, and diagnosis programmes (S35, 96%).

Cross-specialty alignment (Cardiology, Nephrology, Endocrinology, Primary Care & Policy Makers) (S36-39)

Very high levels of agreement suggest strong support for multidisciplinary involvement in CKD management, and that PCPs should form part of the MDT. Given the prominent status of CKD as an emerging global health threat [47], there is a need to ensure that plans and funding are in place to enable optimal care. Stakeholders should engage with both local and national policymakers to ensure that CKD is appropriately prioritised.

Education of clinicians and patients (S40-42)

Most responders agree on the need for up-to-date education for all healthcare professional roles involved in CKD management, and that PCPs require structured education. Education of patients is a low-cost intervention, to encourage kidney healthy lifestyles and improve awareness of CKD among high-risk populations.

Strengths and limitations

274 responses received with good representation from experienced physicians across 11 different countries is a strong basis for consensus, the study was designed to ensure that each country was represented by an equal number of responders. The majority of responders reported more than 10 years' experience in role (159/274), suggesting that the results represent the views of experienced physicians.

This study had some key limitations. Very high levels of agreement were achieved across the statements, this which may suggest that the statements were constructed as to achieve agreement (confirmation bias), or that

For peer review only

perhaps they represent established good practice, a possible improvement to the process might be to provide survey responders (or a sub-set) with the opportunity to add or amend the statements prior to the full survey. It must also be acknowledged that associations with pharmaceutical companies may have led to unconscious bias the development of the consensus statements, but the study was designed to minimise the impact of this bias on results: the identity of steering group members was not disclosed to survey responders, and the identity of responders was not known to either the facilitator or the steering group.

The lack of representation on the Steering Group (and subsequently the wider panel) of members from LMICs was a significant limitation. Whilst the survey covered 11 countries of varying economic status, the range was from lower-middle income to high, a clear gap exists, namely those countries classified as 'low income' (e.g., Sub-Saharan Africa). Consequently, the findings of this study may not be generalisable to LMICs. In order to make such recommendations a further Delphi process would be required specifically to engage with this demographic, this would provide insight into the specific challenges and considerations and allow for comparison with the current dataset.

Aspects of patient choice and empowerment and consideration of the patient experience (outside of treatment outcomes) have not been discussed, this can be considered a limitation as the patient perspective may have significant bearing on the practicability and implementation of CKD management.

RECOMMENDATIONS

1. Early screening for CKD in high-risk groups is cost-effective for the health system (where resources are in place to support intervention)
2. GFR (estimated by the CKD-EPI Creatinine Equation) and uACR (using albumin-to creatinine ratio) should be the screening method of choice for CKD
3. The CKD multi-disciplinary team (MDT) should include the primary care physician to improve early intervention and decision making
4. Intervene early in CKD patients with proven therapies (i.e., SGLT2i, RAASi, DMTs) to delay/prevent progression to kidney failure
5. RAASi therapies should be optimised in all CKD and HF patients
6. Patients with CKD should have their hyperkalaemia managed appropriately when serum potassium level rises above >5.0 mmol/L

For peer review only

7. Chronic hyperkalaemia should be treated with a potassium binder (e.g., patorimer, sodium zirconium cyclosilicate) to allow for the maintenance of disease-modifying therapies
8. Guidelines should be practical with an executive summary/checklist to assist implementation by non-specialist HCPs
9. Guidelines should include clear criteria for when and how to refer to other specialists/MDT
10. Nephrologists should work together to deliver consistent and clear education regarding CKD management
11. Clinicians, professional associations, academic institutions, and patient representative organisations need to engage with policymakers to ensure appropriate plans and funding are in place to deliver optimal CKD care
12. National kidney health programmes should be implemented to drive improvements to screening and diagnosis

CONCLUSION

The Steering Group was able to form a set of recommendations specific to the 11 participant countries ranging from lower-middle income to high income and relevant to other countries with a similar demographic. Implementation of these recommendations has the potential to improve detection of CKD at an earlier stage in patients with risk factors. Earlier diagnosis provides the opportunity for early intervention with DMTs that can slow or halt the progression of CKD, in addition to reducing associated morbidity and mortality.

DECLARATIONS

Data availability statement

All data relevant to the study are included in the article or uploaded as supplementary information.

Ethics statements

Patient consent for publication

Not applicable.

Ethics approval

Not applicable.

Author contributions: All views expressed by the authors are those of the individual and do not represent those of their organisation/place of work. Contributors MA, SAK, MCB, JCJC, YJL, MM, GRD, VSP, SC, TT, CP agreed the

For peer review only

design of the study, formulated and reviewed the statement set. Results of the study were discussed by all contributors as part of a formal Steering Group meeting where commentary was agreed for development into the initial draft manuscript. MA, SAK, MCB, JCJC, YJL, MM, GRD, VSP, SC, TT, CP took an equal role in reviewing the initial manuscript draft and providing comments, and all approved the final draft. MA is the guarantor for the work and/or conduct of the study, had access to the data and controlled the decision to publish.

Acknowledgements: The authors would like to thank Ian Walker and Dal Singh of Triducive Partners Limited for their assistance in survey distribution, analysis of result, writing the initial draft manuscript, and reviewing the final draft.

FUNDING

The study was initiated and funded by AstraZeneca. All authors received funding from AstraZeneca while undertaking this study. AstraZeneca commissioned Triducive Partners Limited to facilitate the project and analyse the responses to the consensus statements in line with the Delphi methodology. After engaging Triducive Partners Limited, AstraZeneca made no contribution to the design and development of the study outside of payment of honoraria. AstraZeneca took no part in the writing, revision or editing of the manuscript except to check that the manuscript contained no promotion of specific medicines and that all recommendations were appropriate to drug label.

For peer review only

CONFLICTS OF INTEREST

M Arici has received payment honoraria for lectures, presentations, speaker bureaus, publication writing or educational events from: Amgen, AstraZeneca, Bayer, Boehringer Ingelheim, Menarini, MSD, Novo Nordisk, Sandoz, and Sanofi. M Arici has also participated in a Data Safety Monitoring Board or Advisory Boards for*:* AstraZeneca, Bayer, Boehringer Ingelheim, and Novo Nordisk. MC Bertoluci has worked as speaker for: Aché, AstraZeneca, Bayer, Boehringer-Ingelheim, Novo-Nordisk, and Servier. JCJ Choo has consulted for, or been on advisory boards for: AstraZeneca, ATC, Bayer, GSK, Nitto Denko, and Pfizer. JCJ Choo has been a speaker for: Abbott, AstraZeneca, Bayer, and Boehringer Ingelheim. JCJ Choo has been on Steering Committees or Data Safety Monitoring Boards for: AstraZeneca, Boehringer Ingelheim, and Novartis, and has received scientific grant funding from: AstraZeneca, ATC, and Nitto Denko. Y-J Lee has been a speaker and Advisory Board member for: AstraZeneca Taiwan. M Madero was the International Society for Nephrology Deputy Chair for Latin America and Caribbean (2019-2023) and is currently the on the council for the International Society for Peritoneal Disease (2022-2024). M Madero has been a consultant for: AstraZeneca, Bayer, and Boehringer Ingelheim, and provided

expert testimony for: AstraZeneca, Bayer, and Boehringer Ingelheim. M Madero has received grants from or has pending grants with: AstraZeneca*, Bayer*, Boehringer, Renal Research Institute*, and Tricida*. M Madero has received travel grants from: AstraZeneca and Medcom. GJ Rosa Diez has been a speaker or Advisory Board member for: Astra Zeneca, Boehringer Ingelheim, Bayer, Fresenius, and Medtronic. S Chung has received honoraria as a speaker or advisory board member from: AstraZeneca, Boehringer Ingelheim, Daewon, Daewoong, Eli Lilly, Korea Pharma, Myoung Poom Medical, Otsuka, Sanofi-Aventis, and Yuhan. C Pollock has been a speaker or participated in advisory boards for Astra Zeneca, Boehringer Ingelheim, Eli Lilly, GlaxoSmithKline, Otsuka, Vifor-CSL. S Assad-Khalil, V Sánchez-Polo, and T Thanachayanont declare no conflict of interest in regard to this study.

For peer review only

REFERENCES

1. Kidney Disease: Improving Global Outcomes (KDIGO) 2022 Clinical Practice Guideline for Diabetes Management in Chronic Kidney Disease Kidney International (2022) 102 (Suppl 5S), S1–S127 S1.
2. Tuttle KR, Alicic RZ, Duru OK, et al. Clinical Characteristics of and Risk Factors for Chronic Kidney Disease Among Adults and Children: An Analysis of the CURE-CKD Registry. JAMA Netw Open. 2019 Dec 2;2(12):e1918169. doi: 10.1001/jamanetworkopen.2019.18169. PMID: 31860111; PMCID: PMC6991307.
3. Kadowaki T, Maegawa H, Watada H, et al. Interconnection between cardiovascular, renal and metabolic disorders: A narrative review with a focus on Japan. Diabetes Obes Metab. 2022 Dec;24(12):2283-2296. doi: 10.1111/dom.14829. Epub 2022 Aug 25. PMID: 35929483; PMCID: PMC9804928.
4. Thomas MC, Cooper ME, Zimmet P. Changing epidemiology of type 2 diabetes mellitus and associated chronic kidney disease. Nat Rev Nephrol. 2016 Feb;12(2):73-81. doi: 10.1038/nrneph.2015.173. Epub 2015 Nov 10. PMID: 26553517.
5. van de Wouw J, Broekhuizen M, Sorop O, et al. Chronic Kidney Disease as a Risk Factor for Heart Failure With Preserved Ejection Fraction: A Focus on Microcirculatory Factors and Therapeutic Targets. Front Physiol. 2019 Sep 4;10:1108. doi: 10.3389/fphys.2019.01108. PMID: 31551803; PMCID: PMC6737277.
6. da Silva LS, Cotta RMM, Moreira TR, et al. Hidden prevalence of chronic kidney disease in hypertensive patients: the strategic role of primary health care. Public Health. 2016 Nov;140:250-257. doi: 10.1016/j.puhe.2016.02.029. Epub 2016 Mar 29. PMID: 27036982.
7. Bikbov B, Purcell C, Levey A, et al. (2020). Global, regional, and national burden of chronic kidney disease, 1990–2017: a systematic analysis for the Global Burden of Disease Study 2017. The Lancet. 395. 709-733. 10.1016/S0140-6736(20)30045-3.
8. Bastos MG, Kirsztajn GM. Chronic kidney disease: importance of early diagnosis, immediate referral and structured interdisciplinary approach to improve outcomes in patients not yet on dialysis. J Bras Nefrol. 2011 Mar;33(1):93-108. English, Portuguese. PMID: 21541469.
9. Kidney Disease: Improving Global Outcomes (KDIGO) CKD Work Group. KDIGO 2012 Clinical Practice Guideline for the Evaluation and Management of Chronic Kidney Disease. Kidney inter., Suppl. 2013; 3: 1–150.
10. Inker LA, Eneanya ND, Coresh J, et al. New Creatinine- and Cystatin C-Based Equations to Estimate GFR without Race. N Engl J Med. 2021 Nov 4;385(19):1737-1749. doi: 10.1056/NEJMoa2102953. Epub 2021 Sep 23. PMID: 34554658; PMCID: PMC8822996.
11. James G, Garcia Sanchez JJ, Carrero JJ, et al. Low Adherence to Kidney Disease: Improving Global Outcomes 2012 CKD Clinical Practice Guidelines Despite Clear Evidence of Utility. Kidney Int Rep. 2022 Jun 8;7(9):2059-2070. doi: 10.1016/j.ekir.2022.05.033. PMID: 36090504; PMCID: PMC9458998.
12. Kidney Disease: Improving Global Outcomes (KDIGO) Blood Pressure Work Group. Kidney Int. 2021 Mar;99(3S):S1-S87. Doi: 10.1016/j.kint.2020.11.003. PMID: 33637192.
13. Al-Ghamdi S, Abu-Alfa A, Alotaibi T, AlSaaidi A, AlSuwaida A, Arici M, Ecder T, El Koraie AF, Ghnaimat M, Hafez MH, Hassan M, Sqalli T. Chronic Kidney Disease Management in the Middle East and Africa: Concerns, Challenges, and Novel Approaches. Int J Nephrol Renovasc Dis. 2023 Apr 6;16:103-112. doi: 10.2147/IJNRD.S363133. PMID: 37051319; PMCID: PMC10084934.
14. Assaad-Khalil S, Elkafrawy N, Khaled M, Mogeib O, Badr H, Rashwan A, Youssef M, Eltamawy K, Mohamed S. A Phase II, Randomized, Double-Blind, Double-Dummy, Active-Controlled Clinical Trial to Investigate the Efficacy and Safety of NW Low-Glu® in Patients Newly Diagnosed with Type 2 Diabetes Mellitus. Evid Based Complement Alternat Med. 2022 Sep 27;2022:9176026. doi: 10.1155/2022/9176026. PMID: 36204125; PMCID: PMC9532066.
15. Bertoluci MC, Silva Júnior WS, Valente F, Araujo LR, Lyra R, de Castro JJ, Raposo JF, Miranda PAC, Boguszewski CL, Hohl A, Duarte R, Salles JEN, Silva-Nunes J, Dores J, Melo M, de Sá JR, Neves JS, Moreira RO, Malachias MVB, Lamounier RN, Malerbi DA, Calliari LE, Cardoso LM, Carvalho MR, Ferreira HJ, Nortadas R, Trujilho FR, Leitão CB, Simões JAR, Dos Reis MIN, Melo P, Marcelino M, Carvalho D. 2023 UPDATE: Luso-Brazilian evidence-based guideline for the management of antidiabetic therapy in type 2 diabetes. Diabetol Metab Syndr. 2023 Jul 19;15(1):160. doi: 10.1186/s13098-023-01121-x. PMID: 37468901; PMCID: PMC10354939.
16. Hsu YT, Lin JY, Lin CJ, Lee YJ, Chang WH. Association of Possible Sarcopenia or Sarcopenia with Body Composition, Nutritional Intakes, Serum Vitamin D Levels, and Physical Activity among Patients with Type 2 Diabetes Mellitus in Taiwan. Nutrients. 2023 Sep 7;15(18):3892. doi: 10.3390/nu15183892. PMID: 37764676; PMCID: PMC10535053.
17. Vasquez Jimenez E, Campos Núñez G, Lerma A, Lerma C, Meave González A, Pérez-Grovas H, López Gil S, Madero M. Neurocognitive Function with Conventional Hemodialysis Versus Post-Dilution Hemofiltration as Initial Treatment in ESKD patients. A randomized controlled trial. The DA-VINCI Study. Blood Purif. 2023 Oct 27. doi: 10.1159/000534823. Epub ahead of print. PMID: 37899042.
18. Glastras SJ, Pollock CA. Targeted identification of risk and treatment of diabetic kidney disease. Nat Rev Nephrol. 2023 Dec 1. doi: 10.1038/s41581-023-00796-9. Epub ahead of print. PMID: 38040863.
19. Barbagelata L, Masson W, Corral P, Lavalle-Cobo A, Nogueira JP, Rosa Diez G. Relationship between lipoprotein(a) levels, cardiovascular outcomes and death in patients with chronic kidney disease: a systematic review of prospective studies. J Nephrol. 2023 Jul;36(6):1549-1559. doi: 10.1007/s40620-023-01590-3. Epub 2023 Apr 8. PMID: 37029883.
20. Koh ES, Kim GH, Chung S. Intrarenal Mechanisms of Sodium-Glucose Cotransporter-2 Inhibitors on Tubuloglomerular Feedback and Natriuresis. Endocrinol Metab (Seoul). 2023 Aug;38(4):359-372. doi: 10.3803/EnM.2023.1764. Epub 2023 Jul 24. PMID: 37482684; PMCID: PMC10475968.
21. Thanachayanont T, Chanpitakkul M, Saetie A, Lekagul S, Tungsanga K. Correlation of the Dietary Protein Intake between Those Estimated from a Short Protein Food-Recall Questionnaire and from 24-Hour Urinary Urea-Nitrogen Excretion in Stages 3-4 Chronic Kidney Disease Patients. Int J Nephrol. 2023 Nov 22;2023:9713045. doi: 10.1155/2023/9713045. PMID: 38033373; PMCID: PMC10686714.
22. Burton JO, Coats AJS, Kovesdy CP, et al. An international Delphi consensus regarding best practice recommendations for hyperkalaemia across the cardiorenal spectrum. Eur J Heart Fail. 2022 Sep;24(9):1467-1477. doi: 10.1002/ejhf.2612. Epub 2022 Aug 4. Erratum in: Eur J Heart Fail. 2023 Mar;25(3):444. PMID: 35791065; PMCID: PMC9804940.

23. Diamond IR, Grant RC, Feldman BM, et al. Defining consensus: a systematic review recommends methodologic criteria for reporting of Delphi studies. J Clin Epidemiol. 2014 Apr;67(4):401-9. doi: 10.1016/j.jclinepi.2013.12.002. PMID: 24581294.

24. Li PK, Garcia-Garcia G, Lui SF, et al. Kidney health for everyone everywhere - from prevention to detection and equitable access to care. Clin Nephrol. 2020 Mar;93(3):111-122. doi: 10.5414/CNWKDEditorial. PMID: 32017699.

25. Komenda P, Ferguson TW, Macdonald K, et al. Cost-effectiveness of primary screening for CKD: a systematic review. Am J Kidney Dis. 2014 May;63(5):789-97. doi: 10.1053/j.ajkd.2013.12.012. Epub 2014 Feb 12. PMID: 24529536.

26. Shlipak MG, Tummalapalli SL, Boulware LE, et al. The case for early identification and intervention of chronic kidney disease: conclusions from a Kidney Disease: Improving Global Outcomes (KDIGO) Controversies Conference. Kidney Int. 2021 Jan;99(1):34-47. doi: 10.1016/j.kint.2020.10.012. Epub 2020 Oct 27. PMID: 33127436.

27. Sammut-Powell C, Sisk R, Budd J, Patel N, Edge M, Cameron R. Development of minimal resource pre-screening tools for chronic kidney disease in people with type 2 diabetes. Future Healthc J. 2022 Nov;9(3):305-309. doi: 10.7861/fhj.2022-0020. PMID: 36561833; PMCID: PMC9761456.

28. Kellum JA, Romagnani P, Ashuntantang G, Ronco C, Zarbock A, Anders HJ. Acute kidney injury. Nat Rev Dis Primers. 2021 Jul 15;7(1):52. doi: 10.1038/s41572-021-00284-z. PMID: 34267223.

29. Schytz PA, Blanche P, Nissen AB, et al. Acute kidney injury and risk of cardiovascular outcomes: A nationwide cohort study. Nefrologia (Engl Ed). 2021 Sep 3:S0211-6995(21)00140-5. English, Spanish. doi: 10.1016/j.nefro.2021.06.007. Epub ahead of print. PMID: 34489123.

30. Thomas M, Magwire M, Gosch K, et al. Cardiometabolic Center of Excellence: A Novel Care Delivery Model for Secondary Prevention of Cardiovascular Disease in Type 2 Diabetes. Circ Cardiovasc Qual Outcomes. 2021 Oct;14(10):e007682. doi: 10.1161/CIRCOUTCOMES.120.007682. Epub 2021 Sep 30. PMID: 34587753; PMCID: PMC8530947.

31. Weber C, Beaulieu M, Djurdjev O, et al. Towards rational approaches of health care utilization in complex patients: an exploratory randomized trial comparing a novel combined clinic to multiple specialty clinics in patients with renal disease-cardiovascular disease-diabetes. Nephrol Dial Transplant. 2012 Oct;27 Suppl 3:iii104-10. doi: 10.1093/ndt/gfr292. Epub 2011 Jun 30. PMID: 21719715.

32. Spaak J. Novel combined management approaches to patients with diabetes, chronic kidney disease and cardiovascular disease. J R Coll Physicians Edinb. 2017 Mar;47(1):83-87. doi: 10.4997/JRCPE.2017.118. PMID: 28569290.

33. ISN-Global Kidney Health Atlas. International Society of Nephrology. 3rd Edition. 2023.

34. Nichols GA, Ustyugova A, Déruaz-Luyet A, O'Keeffe-Rosetti M, Brodovicz KG. Health Care Costs by Type of Expenditure across eGFR Stages among Patients with and without Diabetes, Cardiovascular Disease, and Heart Failure. J Am Soc Nephrol. 2020 Jul;31(7):1594-1601. doi: 10.1681/ASN.2019121308. Epub 2020 Jun 2. PMID: 32487562; PMCID: PMC7350988.

35. Yang A, Shi M, Lau ESH, et al. Clinical outcomes following discontinuation of renin-angiotensin-system inhibitors in patients with type 2 diabetes and advanced chronic kidney disease: A prospective cohort study. EClinicalMedicine. 2022 Nov 24;55:101751. doi: 10.1016/j.eclinm.2022.101751. PMID: 36457651; PMCID: PMC9706514.

36. Fu EL, Evans M, Clase CM, et al. Stopping Renin-Angiotensin System Inhibitors in Patients with Advanced CKD and Risk of Adverse Outcomes: A Nationwide Study. J Am Soc Nephrol. 2021 Feb;32(2):424-435. doi: 10.1681/ASN.2020050682. Epub 2020 Dec 28. PMID: 33372009; PMCID: PMC8054897.

37. Qiao Y, Shin J, Chen TK, et al. Association Between Renin-Angiotensin System Blockade Discontinuation and All-Cause Mortality Among Persons With Low Estimated Glomerular Filtration Rate. JAMA Intern Med. 2020;180(5):718–726. doi:10.1001/jamainternmed.2020.0193.

38. Clark AL, Kalra PR, Petrie MC, Mark PB, Tomlinson LA, Tomson CR. Change in renal function associated with drug treatment in heart failure: national guidance. Heart. 2019 Jun;105(12):904-910. doi: 10.1136/heartjnl-2018-314158. PMID: 31118203; PMCID: PMC6582720.

39. Morales E, Cravedi P, Manrique J. Management of Chronic Hyperkalemia in Patients With Chronic Kidney Disease: An Old Problem With News Options. Front Med (Lausanne). 2021 Jun 4;8:653634. doi: 10.3389/fmed.2021.653634. PMID: 34150795; PMCID: PMC8213200.

40. Kim GH. Pharmacologic Treatment of Chronic Hyperkalemia in Patients with Chronic Kidney Disease. Electrolyte Blood Press. 2019 Jun;17(1):1-6. doi: 10.5049/EBP.2019.17.1.1. Epub 2019 Jun 30. PMID: 31338108; PMCID: PMC6629599.

41. Clase CM, Carrero JJ, Ellison DH, et al. Potassium homeostasis and management of dyskalemia in kidney diseases: conclusions from a Kidney Disease: Improving Global Outcomes (KDIGO) Controversies Conference. Kidney Int. 2020 Jan;97(1):42-61. doi: 10.1016/j.kint.2019.09.018. Epub 2019 Oct 10. PMID: 31706619.

42. Wang AY. Optimally managing hyperkalemia in patients with cardiorenal syndrome. Nephrol Dial Transplant. 2019 Dec 1;34(Suppl 3):iii36-iii44. Doi: 10.1093/ndt/gfz225. PMID: 31800079.

43. Silva-Cardoso J, Brito D, Frazão JM, et al. Management of RAASi-associated hyperkalemia in patients with cardiovascular disease. Heart Fail Rev. 2021 Jul;26(4):891-896. Doi: 10.1007/s10741-020-10069-3. Epub 2021 Feb 18. PMID: 33599908; PMCID: PMC8149346.

44. Epstein M, Reaven NL, Funk SE, McGaughey KJ, Oestreicher N, Knispel J. Evaluation of the treatment gap between clinical guidelines and the utilization of renin-angiotensin-aldosterone system inhibitors. Am J Manag Care. 2015 Sep;21(11 Suppl):S212-20. PMID: 26619183.

45. Trevisan M, de Deco P, Xu H, et al. Incidence, predictors, and clinical management of hyperkalaemia in new users of mineralocorticoid receptor antagonists. Eur J Heart Fail. 2018 Aug;20(8):1217-1226. Doi: 10.1002/ejhf.1199. Epub 2018 Apr 18. Erratum in: Eur J Heart Fail. 2019 Apr;21(4):540. PMID: 29667759; PMCID: PMC6607478.

46. Fraser SD, Roderick PJ, May CR, et al. The burden of comorbidity in people with chronic kidney disease stage 3: a cohort study. BMC Nephrol. 2015 Dec 1;16:193. doi: 10.1186/s12882-015-0189-z. Erratum in: BMC Nephrol. 2020 Dec 21;21(1):543. PMID: 26620131; PMCID: PMC4666158.

47. Lee WC, Lee YT, Li LC, et al. The Number of Comorbidities Predicts Renal Outcomes in Patients with Stage 3⁻5 Chronic Kidney Disease. J Clin Med. 2018 Nov 28;7(12):493. doi: 10.3390/jcm7120493. PMID: 30486496; PMCID: PMC6306906.

For peer review only - http://bmjopen.bmj.com/site/about/guidelines.xhtml

Appendix: Supplementary Data

Table S1: Proportion of responses for each agreement level by statement

<<Table S1>>

Figure S1: Proportion of responses for each agreement level by statement

<<Figure S1>>

Table S2: Anonymized Round 2 Response Data

<<Table S2>>

For peer review only

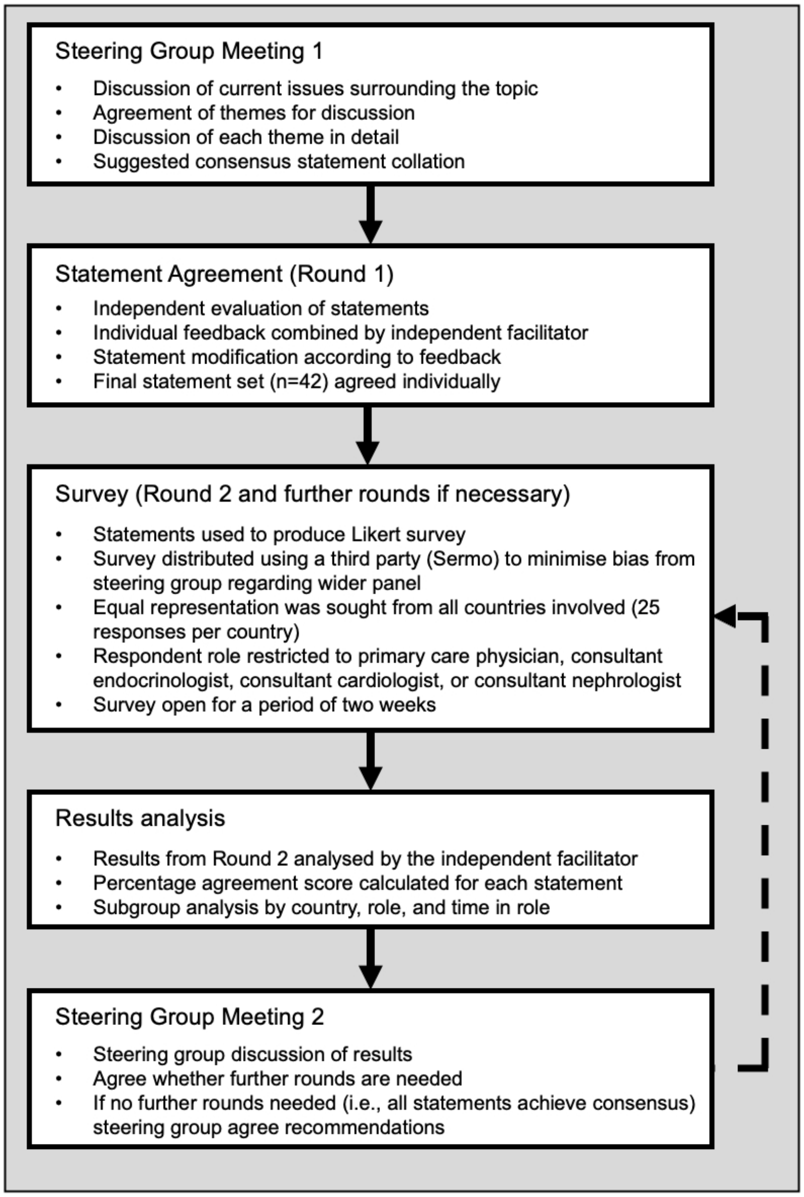

Figure 1: Study flow diagram

282x418mm (72 x 72 DPI)

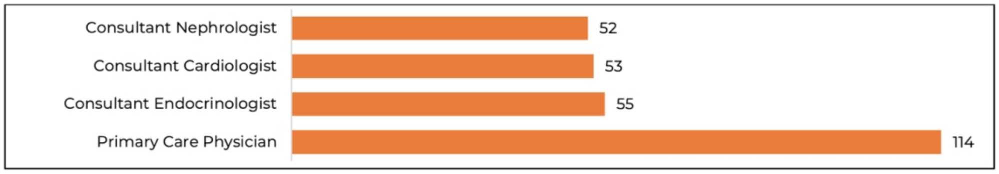

Figure 2: Respondents by role

525x91mm (72 x 72 DPI)

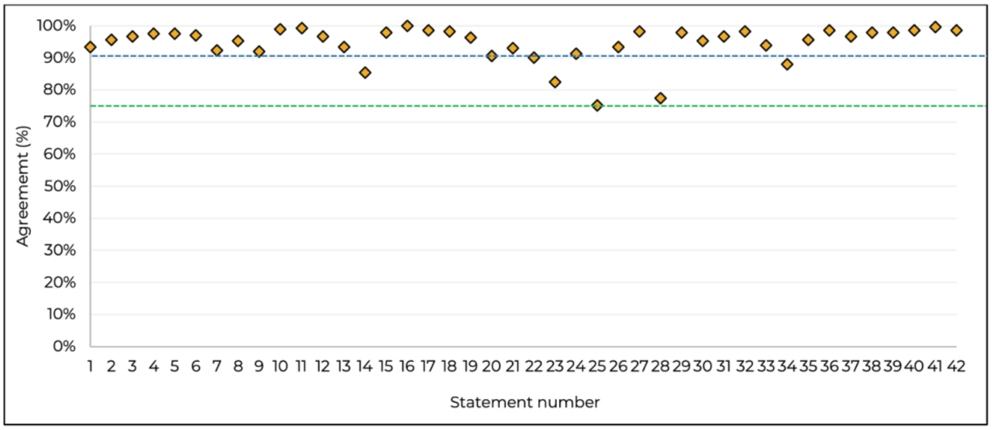

Figure 3: Overall consensus agreement levels by statementa

521x225mm (72 x 72 DPI)

No:	Statement:	Strongly agree	Tend to agree	Tend to disagree	Strongly disagree	Overall
**Earlier identification & screening of CKD**						
**1.**	Late diagnosis is a lost opportunity for early management of CKD which can slow disease progression	77%	16%	3%	4%	**93%**
**2.**	National screening and diagnostic programmes for CKD are essential to identifying patients at earlier stages of CKD	69%	27%	3%	2%	**96%**
**3.**	A simple and practical definition of 'high-risk' should be established to support cost-effective screening	65%	32%	2%	1%	**97%**
**4.**	Early screening for CKD in high-risk groups is cost-effective for the health system (where resources are in place to support intervention)	71%	27%	1%	1%	**97%**
**5.**	Primary care physicians and specialists (cardiologists, endocrinologists, nephrologists, diabetologists, etc.) should routinely screen for CKD in patients with risk factors	76%	21%	1%	1%	**97%**
**6.**	Patients with risk factors (e.g., elderly, diabetes, hypertension, dyslipidaemia, obesity, family history of CKD) should be screened at least annually for CKD	81%	16%	2%	1%	**97%**
**7.**	Patients with CKD should be screened for heart failure (including those with preserved ejection fraction (HFpEF))	46%	46%	6%	2%	**92%**
**8.**	GFR estimated by the CKD-EPI Creatinine Equation (2021) and albuminuria (using albumin-to-creatinine ratio) should be the screening method of choice for CKD	55%	41%	4%	1%	**95%**
**9.**	Confirmatory testing of abnormalities of kidney function or structure at 3 months is required to establish a CKD diagnosis	48%	44%	7%	1%	**92%**
**Risk factors for CKD in cardio-renal-metabolic patients**						
**10.**	A holistic view of cardio-renal-metabolic disease states is critical to provide integrated patient-centred care to individuals with CKD	74%	25%	0%	1%	**99%**
**11.**	CKD progressively increases the risk of CVD, including heart failure and overall mortality	75%	24%	0%	1%	**99%**
**12.**	Frequency of hyperkalaemia increases as CKD progresses	62%	34%	3%	1%	**97%**
**13.**	AKI is a risk factor for CKD and chronic heart failure	57%	37%	5%	1%	**93%**
**14.**	Infection, including COVID-19, can cause AKI and is therefore a risk factor for CKD	42%	43%	14%	1%	**85%**
**15.**	Nephrotoxic medications should be modified, and risk/benefit profiles should be assessed in patients with, or at risk of CKD	72%	26%	1%	1%	**98%**
**Holistic management of CKD in cardio-renal-metabolic patients**						
**16.**	Lifestyle factors such as diet, smoking, exercise must be optimised for CKD patients	83%	17%	0%	0%	**100%**
**17.**	Disease modifying therapies for CKD (e.g., SGLT2i, RAASi, MRA) should be used where indicated to manage progression of CKD	69%	29%	1%	0%	**99%**
**18.**	SGLT2i and RAASi have a complementary cardio-renal protective action	61%	37%	1%	0%	**98%**
**19.**	Early use of SGLT2i could prevent the development and progression of CKD patients with type 2 diabetes and heart failure	62%	34%	3%	0%	**96%**
**20.**	Early use of SGLT2i can slow progression of CKD in patients without diabetes	45%	45%	9%	1%	**91%**
**21.**	A small decrease of eGFR (~10%) may be expected on initiation of CKD disease-modifying therapies	39%	54%	7%	0%	**93%**
**22.**	De-escalation or discontinuation of RAASi therapy is associated with worse cardiovascular and renal outcomes in the CKD patient	33%	57%	9%	1%	**90%**
**23.**	Disease modifying therapies for CKD should only be stopped as a last resort	33%	50%	15%	3%	**82%**
**24.**	Novel K binders (i.e., patiromer and sodium zirconium cyclosilicate) are an option to manage hyperkalaemia and prevent de-escalation or downtitration of RAASi	27%	65%	8%	0%	**91%**
**25.**	Action to manage hyperkalaemia in the CKD patient should be taken when serum potassium level reaches 5.0mmol/L	22%	54%	20%	5%	**75%**
**26.**	Action to manage hyperkalaemia in the CKD patient should be taken when serum potassium level reaches 5.5 mmol/L	53%	40%	5%	1%	**93%**

For peer review only

**27.**	Treatment targets such as blood pressure, blood glucose, lipid levels should be maintained for both kidney and cardiac outcomes in CKD patients	81%	17%	1%	0%	**98%**
**Guidelines**						
**28.**	Implementation of evidence-based CKD guidelines is suboptimal and should be improved	29%	48%	20%	2%	**77%**
**29.**	Cardiology, nephrology, and endocrinology guidelines for CKD should be aligned	62%	36%	2%	0%	**98%**
**30.**	Primary care physicians have an important role in implementing guidelines	66%	30%	4%	1%	**95%**
**31.**	Guidelines should be practical with an executive summary / checklist to assist implementation by non-specialist HCPs	65%	31%	3%	0%	**97%**
**32.**	Guidelines should include clear criteria for when and how to refer to other specialists/MDT	69%	30%	2%	0%	**98%**
**33.**	Guidelines should reflect differences in population phenotypes and be locally adapted where needed	58%	35%	6%	0%	**94%**
**34.**	Patient perspective is crucial in developing guidelines	42%	46%	11%	1%	**88%**
**35.**	Investment is needed to shift the approach to primary prevention rather than end stage kidney disease management	69%	26%	3%	1%	**96%**
**Cross-specialty alignment (Cardiology, Nephrology, Endocrinology, Primary Care & Policy Makers)**						
**36.**	Patients with CKD should be managed by a multi-disciplinary team (MDT) where possible with an agreed management plan	67%	31%	1%	0%	**99%**
**37.**	The CKD multi-disciplinary team (MDT) should include the primary care physician to improve early intervention and decision making	65%	32%	3%	0%	**97%**
**38.**	The care pathway for CKD should be designed to minimise the impact of organisational barriers	58%	39%	2%	0%	**98%**
**39.**	Clinicians, professional associations, academic institutions, and patient representative organisations need to engage with policy makers to ensure appropriate plans and funding are in place to deliver optimal CKD care	67%	31%	2%	0%	**98%**
**Education of clinicians and patients**						
**40.**	Up-to-date healthcare professional (HCP) education on the management of patients with CKD and associated guidelines is needed	72%	27%	1%	0%	**99%**
**41.**	Structured education of primary care physicians on screening and early detection improves patient outcomes	76%	24%	0%	0%	**100%**
**42.**	Therapeutic patient education is key for them to understand the consequences of CKD and how to manage it through lifestyle modification and appropriate use of therapies	73%	26%	1%	0%	**99%**

For peer review only

ID	Role	Country	Experience	No. CKD patients every 3 months	Statement Number																																								
					1	2	3	4	5	6	7	8	9	10	11	12	13	14	15	16	17	18	19	20	21	22	23	24	25	26	27	28	29	30	31	32	33	34	35	36	37	38	39	40	41	42
1	Primary Care Physician	AUS	More than 10 years	10-19 patients	1	1	1	1	1	1	2	1	2	1	1	2	1	2	1	1	1	2	2	2	2	2	2	2	2	3	1	3	1	2	1	1	2	2	2	2	1	1	2	2	2	2
2	Primary Care Physician	AUS	More than 10 years	20-29 patients	1	1	1	1	1	1	1	1	1	1	1	2	1	1	1	1	1	1	1	2	2	2	2	2	2	1	1	1	1	1	1	1	1	1	1	1	1	1	1	1	1	1
3	Consultant Endocrinologist	AUS	More than 10 years	100+ patients	1	1	1	1	1	1	1	1	2	1	1	1	1	1	1	1	1	2	2	2	1	2	1	2	2	1	1	1	1	1	1	1	1	1	1	1	1	1	1	1	2	1
4	Primary Care Physician	AUS	More than 10 years	80-89 patients	1	2	2	1	2	1	2	2	2	2	1	2	2	1	1	2	2	2	2	2	2	3	2	2	2	3	1	3	2	1	2	1	1	2	1	1	1	1	1	1	1	1
5	Primary Care Physician	AUS	6 - 10 years	40-49 patients	2	2	2	2	2	1	2	3	2	2	1	1	3	3	2	1	2	3	3	3	2	1	2	3	1	1	1	3	2	1	1	1	1	1	1	1	1	1	1	2	1	2
6	Primary Care Physician	AUS	More than 10 years	80-89 patients	1	2	1	1	1	1	2	1	2	2	1	2	1	1	1	1	1	2	1	1	2	2	3	3	3	2	1	3	3	1	2	1	2	2	1	1	1	1	1	1	2	1
7	Primary Care Physician	AUS	More than 10 years	30-39 patients	1	2	1	2	2	3	3	3	2	2	2	1	3	2	3	2	2	2	3	1	1	4	3	2	3	1	1	1	2	2	1	3	2	3	4	1	1	2	1	2	2	2
8	Primary Care Physician	AUS	6 - 10 years	90-99 patients	1	1	1	1	1	1	1	1	2	1	1	2	2	2	1	1	1	2	1	2	2	2	2	2	2	1	1	2	1	1	1	1	2	2	1	1	1	1	1	1	1	1
9	Primary Care Physician	AUS	6 - 10 years	50-59 patients	1	2	2	2	1	1	1	2	2	1	1	1	1	1	1	1	2	1	1	2	2	3	1	3	2	3	1	2	1	1	1	1	1	2	1	1	1	1	1	2	1	1
10	Primary Care Physician	AUS	More than 10 years	40-49 patients	2	2	1	2	1	1	3	1	1	1	1	2	2	2	1	1	1	1	1	1	2	1	1	2	4	2	1	1	1	1	2	1	1	1	2	1	2	2	2	1	1	2
11	Primary Care Physician	AUS	6 - 10 years	40-49 patients	1	2	1	2	2	1	1	2	2	2	2	1	2	3	1	1	1	1	1	1	2	2	2	3	2	1	1	2	2	1	1	1	1	1	2	2	1	1	2	1	2	2
12	Consultant Cardiologist	AUS	6 - 10 years	50-59 patients	1	2	2	2	2	2	1	2	2	1	1	2	2	2	2	2	2	2	2	2	3	2	2	2	3	2	2	2	2	2	2	3	2	4	2	2	3	2	2	2	2	2
13	Consultant Cardiologist	AUS	6 - 10 years	50-59 patients	2	2	1	1	2	2	2	1	1	2	1	3	2	3	2	1	1	2	2	2	1	2	2	1	3	2	2	2	2	1	1	2	2	2	1	2	1	2	1	2	2	1
14	Consultant Cardiologist	AUS	More than 10 years	70-79 patients	2	2	1	2	1	2	1	2	2	1	2	1	1	1	2	1	1	2	1	2	2	2	2	2	1	2	1	2	2	2	2	2	2	2	1	2	1	2	2	2	2	2
15	Consultant Cardiologist	AUS	More than 10 years	100+ patients	1	1	1	1	1	1	1	1	1	1	1	2	1	2	1	2	1	2	1	2	2	1	2	2	2	2	1	2	1	2	1	2	2	3	1	2	2	1	2	2	2	2
16	Consultant Cardiologist	AUS	More than 10 years	100+ patients	1	1	1	1	1	1	1	1	1	1	1	1	1	1	1	1	1	1	1	1	1	1	1	2	1	2	1	2	1	1	1	1	1	1	2	1	1	1	1	1	1	1
17	Primary Care Physician	MEX	6 - 10 years	60-69 patients	1	1	1	1	1	1	2	2	1	1	1	1	2	2	1	1	1	1	1	2	2	2	2	1	2	2	1	2	1	1	1	2	1	2	1	1	1	1	1	1	1	1
18	Consultant Cardiologist	MEX	More than 10 years	30-39 patients	1	1	1	1	1	1	2	1	1	1	1	1	1	2	1	1	1	1	1	1	1	1	1	1	2	1	1	1	1	1	1	1	1	2	1	1	1	1	1	1	1	1
19	Primary Care Physician	TWN	More than 10 years	10-19 patients	1	1	1	1	1	1	1	1	1	1	1	1	2	2	1	1	1	1	1	1	1	1	1	1	2	1	1	1	1	1	1	1	1	1	1	1	1	1	1	1	1	1
20	Consultant Endocrinologist	MEX	More than 10 years	60-69 patients	2	1	2	1	1	1	2	1	2	1	2	3	3	2	2	1	1	2	2	1	2	3	2	2	3	2	1	1	1	2	2	1	1	3	2	2	1	2	2	2	1	2
21	Primary Care Physician	MEX	6 - 10 years	100+ patients	1	1	1	1	1	1	1	2	1	1	1	1	2	1	1	1	1	1	1	3	1	1	1	2	2	2	1	1	1	1	1	1	1	1	1	1	1	1	1	1	1	1
22	Primary Care Physician	TUR	6 - 10 years	60-69 patients	1	1	1	1	1	1	1	2	1	1	1	1	1	1	1	1	2	2	2	2	2	2	2	1	1	1	1	3	2	1	1	1	1	1	1	1	1	1	1	1	1	1
23	Primary Care Physician	TUR	Less than 5 Years	10-19 patients	1	1	1	1	2	1	2	2	2	1	1	1	1	2	1	1	1	1	1	2	2	2	1	2	3	2	1	3	1	3	1	1	1	1	1	1	1	1	1	1	1	1
24	Consultant Nephrologist	MEX	6 - 10 years	30-39 patients	1	1	1	1	2	1	1	1	1	1	1	1	1	1	1	1	1	1	1	1	1	2	3	2	2	2	1	3	2	2	2	2	1	2	2	2	2	2	2	1	1	1
25	Consultant Endocrinologist	BRA	More than 10 years	100+ patients	1	1	1	1	1	1	1	1	1	1	1	2	1	1	1	1	1	1	1	1	1	1	1	2	2	2	1	1	1	1	1	1	1	1	1	1	1	1	1	1	1	1
26	Primary Care Physician	TUR	Less than 5 Years	10-19 patients	2	1	2	2	2	1	1	1	2	2	2	2	2	2	2	2	2	2	2	2	2	2	2	2	2	2	2	2	2	2	2	2	2	2	2	2	2	2	2	2	2	2
27	Consultant Cardiologist	MEX	Less than 5 Years	50-59 patients	1	1	1	1	1	1	1	2	1	1	1	2	2	2	2	1	1	1	1	1	1	1	1	2	2	1	1	1	1	1	2	2	2	3	1	1	1	1	1	1	1	2
28	Consultant Nephrologist	TUR	Less than 5 Years	30-39 patients	2	1	2	4	3	2	2	1	1	1	1	1	1	2	1	2	1	1	2	1	3	2	2	2	1	1	1	2	1	1	1	1	1	2	1	1	2	1	1	1	1	1
29	Primary Care Physician	TUR	Less than 5 Years	10-19 patients	1	1	1	1	2	2	2	2	2	1	1	1	1	2	1	1	1	2	2	2	2	2	1	2	2	1	1	2	1	1	1	1	1	1	1	1	1	1	1	2	2	2
30	Primary Care Physician	TUR	Less than 5 Years	10-19 patients	4	2	4	1	3	1	3	4	4	1	1	4	4	4	2	1	4	4	4	4	1	4	4	4	4	2	4	4	4	4	4	1	3	4	4	3	4	4	1	4	4	3
31	Primary Care Physician	BRA	6 - 10 years	30-39 patients	2	1	2	1	1	1	3	1	2	1	2	1	2	3	1	1	2	1	2	2	3	2	2	3	1	3	1	2	2	1	1	1	1	2	1	1	1	1	1	1	1	1
32	Consultant Nephrologist	MEX	6 - 10 years	80-89 patients	1	1	2	1	1	1	1	1	1	1	1	1	1	1	1	1	2	1	1	2	1	1	2	2	3	1	1	2	1	2	1	1	1	1	1	1	2	2	2	1	1	1
33	Primary Care Physician	BRA	More than 10 years	100+ patients	1	3	2	2	1	1	1	1	2	1	2	1	2	2	2	1	2	2	2	2	2	2	3	2	3	2	1	2	2	1	1	2	3	2	2	1	1	1	1	2	1	1
34	Primary Care Physician	TUR	6 - 10 years	10-19 patients	1	1	1	1	1	1	1	1	1	1	1	1	1	1	1	1	1	1	1	1	1	1	1	1	4	1	1	2	2	3	2	1	1	1	1	1	1	1	1	1	1	1
35	Consultant Endocrinologist	BRA	More than 10 years	50-59 patients	1	1	1	1	1	1	1	1	1	1	1	1	1	1	1	1	1	1	1	2	1	1	2	2	2	1	1	1	1	2	1	1	1	1	1	1	2	1	2	1	1	1
36	Primary Care Physician	TUR	Less than 5 Years	10-19 patients	1	1	1	1	2	1	1	1	1	1	1	1	1	1	1	1	1	1	1	1	1	1	1	1	1	1	1	1	1	1	1	1	1	1	1	1	1	1	1	1	1	1
37	Primary Care Physician	BRA	6 - 10 years	80-89 patients	1	1	1	1	2	1	2	1	2	1	2	2	1	1	1	1	1	1	3	3	2	1	1	1	1	1	1	1	2	1	1	1	1	3	1	1	2	1	2	1	1	1
38	Consultant Endocrinologist	TUR	6 - 10 years	20-29 patients	1	1	2	2	2	1	2	2	1	2	2	1	1	1	1	1	2	2	2	2	2	2	2	2	1	1	1	2	2	2	2	2	2	2	2	2	2	2	2	2	2	2
39	Primary Care Physician	BRA	More than 10 years	100+ patients	1	1	1	1	1	1	1	1	2	1	1	1	1	1	1	1	1	1	1	1	1	1	1	1	1	1	1	1	1	1	1	1	1	1	1	1	1	1	1	1	1	1
40	Primary Care Physician	TUR	Less than 5 Years	100+ patients	2	2	1	1	1	1	1	2	3	1	2	1	1	1	1	1	2	1	2	2	1	2	3	2	2	1	1	2	1	1	1	1	1	2	1	1	1	1	1	1	1	1
41	Primary Care Physician	TUR	Less than 5 Years	10-19 patients	3	4	2	2	1	1	1	2	2	3	1	1	1	1	1	1	1	2	1	1	1	1	1	1	1	1	2	1	1	1	1	1	1	1	1	1	2	2	3	1	1	2
42	Primary Care Physician	BRA	6 - 10 years	70-79 patients	2	1	2	2	2	1	3	2	3	1	1	2	2	1	1	1	1	1	2	1	2	2	3	3	1	2	1	2	2	1	1	3	1	3	1	1	1	2	2	2	1	1
43	Consultant Cardiologist	TUR	6 - 10 years	20-29 patients	1	2	1	2	1	1	2	2	2	1	1	2	1	1	2	1	1	1	1	1	3	1	4	1	2	3	1	2	2	1	1	1	1	1	2	1	1	1	2	2	2	1
44	Consultant Cardiologist	TUR	More than 10 years	60-69 patients	1	2	1	1	1	1	1	2	1	1	1	2	2	2	1	1	1	1	1	2	2	2	2	2	2	1	1	2	1	3	2	1	1	2	1	1	2	2	1	1	1	1
45	Consultant Nephrologist	MEX	6 - 10 years	40-49 patients	2	1	1	1	1	1	2	1	2	2	2	1	3	3	2	1	1	1	1	3	2	2	2	2	3	2	1	2	1	1	1	1	2	3	1	2	2	2	1	1	1	1
46	Consultant Endocrinologist	BRA	More than 10 years	10-19 patients	1	2	2	1	1	1	1	1	1	1	1	2	2	1	1	2	1	1	2	2	2	1	2	2	3	1	1	2	1	2	1	1	2	2	2	1	1	2	2	1	1	1
47	Primary Care Physician	BRA	6 - 10 years	40-49 patients	1	1	2	2	2	1	2	3	2	2	1	2	1	2	2	1	1	1	2	2	2	2	1	3	2	2	1	1	1	1	1	1	1	1	1	1	1	2	2	1	1	1
48	Consultant Nephrologist	BRA	More than 10 years	100+ patients	1	2	2	2	2	2	2	2	2	2	2	2	2	2	1	2	2	2	1	1	2	2	2	2	2	2	2	2	2	2	2	2	2	2	2	2	2	2	2	1	2	2
49	Primary Care Physician	BRA	More than 10 years	80-89 patients	1	2	1	3	1	1	1	2	2	2	1	1	3	2	1	1	1	1	1	1	2	1	3	3	4	3	2	2	1	2	1	2	4	1	1	1	2	2	2	3	1	2
50	Primary Care Physician	MEX	More than 10 years	40-49 patients	1	2	1	1	1	1	1	1	2	2	1	1	1	1	1	1	1	1	1	1	2	2	2	2	2	1	1	1	1	1	1	2	1	2	1	2	2	1	2	1	1	2
51	Consultant Endocrinologist	MEX	More than 10 years	60-69 patients	1	1	2	1	1	1	2	2	1	1	1	1	2	2	1	1	1	1	2	1	1	2	2	2	2	1	1	1	1	2	2	1	2	1	1	1	2	1	1	1	2	1
52	Primary Care Physician	MEX	More than 10 years	60-69 patients	2	2	1	2	1	1	1	2	2	1	1	2	1	1	1	1	2	1	1	1	1	2	2	1	2	1	1	2	1	1	1	1	1	1	1	1	1	1	1	1	1	1
53	Consultant Cardiologist	BRA	6 - 10 years	40-49 patients	2	2	2	2	2	2	2	2	2	2	2	2	2	2	2	2	2	2	2	2	2	2	2	2	2	2	2	3	2	3	2	2	2	2	2	3	3	3	2	2	2	2
54	Primary Care Physician	BRA	Less than 5 Years	100+ patients	1	1	1	1	1	1	1	1	1	1	1	2	2	2	1	1	1	1	1	1	2	2	1	2	4	2	1	2	2	2	1	1	1	1	1	1	1	1	1	1	1	1
55	Primary Care Physician	BRA	More than 10 years	40-49 patients	1	1	1	1	1	1	1	1	1	1	1	1	1	1	1	1	1	1	1	1	1	1	2	2	2	1	2	2	2	1	1	1	2	1	2	1	2	2	1	1	2	2
56	Consultant Nephrologist	MEX	6 - 10 years	100+ patients	1	2	1	1	1	1	2	1	1	2	1	1	1	1	1	2	1	1	1	1	2	2	2	1	3	2	1	2	1	3	2	1	1	1	1	2	1	1	1	1	1	2
57	Consultant Endocrinologist	BRA	More than 10 years	90-99 patients	1	1	1	1	1	1	1	2	2	1	1	2	1	1	1	1	1	1	1	2	2	2	2	1	2	1	2	2	1	1	1	1	2	2	2	1	2	2	2	2	1	1
58	Primary Care Physician	MEX	More than 10 years	100+ patients	2	2	1	1	1	1	1	2	2	1	1	1	2	1	2	2	1	1	1	2	2	2	3	2	2	2	1	2	1	2	1	1	1	1	1	2	2	3	2	2	1	1
59	Consultant Cardiologist	TUR	6 - 10 years	90-99 patients	2	1	1	1	1	1	1	2	2	1	1	2	1	2	2	1	3	2	1	3	1	2	1	2	2	1	1	2	2	4	2	2	1	2	2	2	3	2	2	1	2	3
60	Consultant Nephrologist	MEX	More than 10 years	100+ patients	1	1	1	1	1	1	1	1	1	1	1	1	1	1	1	1	1	1	1	1	1	1	1	1	1	1	1	2	1	1	1	1	1	1	1	1	1	1	1	1	1	1
61	Primary Care Physician	MEX	6 - 10 years	100+ patients	2	2	3	2	3	3	2	3	3	2	1	2	2	3	3	2	2	2	3	3	2	2	3	3	2	2	2	2	2	2	2	2	2	2	2	2	2	2	2	2	2	2
62	Consultant Endocrinologist	MEX	6 - 10 years	100+ patients	1	1	1	1	1	1	1	1	1	2	1	1	1	1	1	1	1	1	1	1	1	1	3	2	2	1	1	2	1	1	1	1	1	2	1	1	1	1	1	1	1	1
63	Consultant Endocrinologist	MEX	More than 10 years	20-29 patients	1	1	1	1	1	1	1	1	1	1	1	1	1	1	1	1	1	1	1	1	2	2	1	2	1	2	1	3	1	2	1	1	1	1	1	1	1	1	1	1	1	1
64	Consultant Endocrinologist	MEX	More than 10 years	90-99 patients	1	2	2	1	1	2	3	3	3	1	2	2	2	3	3	1	3	2	2	2	2	1	2	2	3	2	3	2	2	1	3	2	2	3	3	1	2	2	3	1	2	3
65	Consultant Cardiologist	TUR	6 - 10 years	100+ patients	2	2	2	1	2	2	2	2	2	2	1	1	1	2	1	1	1	1	1	1	2	2	2	2	3	2	2	3	3	2	2	3	3	2	4	2	2	2	2	3	2	2
66	Consultant Endocrinologist	ARG	More than 10 years	50-59 patients	1	1	2	1	1	1	2	1	1	1	1	1	3	3	1	1	1	2	2	1	2	1	2	2	2	2	3	2	1	1	1	1	1	2	1	1	1	2	2	1	1	1
67	Consultant Endocrinologist	AUS	Less than 5 Years	70-79 patients	1	1	2	1	1	1	1	1	2	1	1	1	1	1	1	1	1	1	1	2	1	2	2	1	2	1	1	1	1	1	1	2	1	2	1	1	1	1	1	1	1	1
68	Consultant Cardiologist	BRA	More than 10 years	20-29 patients	1	2	2	2	1	1	1	1	1	2	1	2	1	2	1	1	1	2	2	2	2	2	2	2	3	2	2	2	2	2	1	1	2	2	2	2	2	2	2	2	1	1
69	Consultant Cardiologist	BRA	More than 10 years	50-59 patients	1	1	1	1	1	1	1	1	1	1	1	1	1	1	1	1	1	1	1	1	3	2	3	3	1	3	2	3	3	2	2	2	2	2	2	1	1	1	1	1	1	1
70	Consultant Cardiologist	MEX	More than 10 years	60-69 patients	3	4	4	4	4	4	4	3	3	4	4	4	4	4	4	1	1	1	1	2	2	2	2	3	2	1	1	1	1	2	2	1	2	3	1	1	2	1	2	1	1	1
71	Consultant Nephrologist	BRA	More than 10 years	70-79 patients	2	1	2	2	1	1	1	1	2	2	2	2	2	2	2	2	2	2	2	2	2	3	2	3	3	2	2	2	2	1	2	1	3	2	2	2	2	1	2	1	1	1
72	Consultant Cardiologist	BRA	More than 10 years	100+ patients	1	1	1	1	1	1	1	2	2	1	1	1	1	1	1	1	1	1	1	1	1	2	2	2	1	1	1	2	2	3	2	2	3	1	1	1	1	1	1	1	1	1
73	Consultant Nephrologist	TUR	6 - 10 years	100+ patients	1	1	1	1	1	1	1	1	1	1	1	1	1	1	1	1	1	1	2	1	1	1	3	1	1	1	1	4	1	1	1	1	1	1	1	1	1	1	1	1	1	1
74	Consultant Endocrinologist	TUR	More than 10 years	90-99 patients	1	1	1	1	1	1	1	1	1	1	1	1	1	1	1	1	1	1	1	1	1	1	1	1	1	1	1	2	1	2	1	1	1	2	1	1	1	1	1	1	1	1
75	Consultant Cardiologist	BRA	More than 10 years	20-29 patients	2	1	2	2	1	1	2	2	3	2	1	2	2	1	2	1	1	2	1	2	3	2	2	2	2	2	1	1	2	3	1	1	2	2	2	1	2	2	1	1	1	1
76	Primary Care Physician	KOR	6 - 10 years	40-49 patients	4	1	2	1	2	1	2	2	3	1	1	2	2	3	1	2	2	2	2	3	2	2	3	2	3	2	1	1	1	1	1	1	2	1	2	1	1	1	1	2	1	1
77	Consultant Cardiologist	TUR	Less than 5 Years	20-29 patients	1	1	1	1	1	1	1	1	2	1	1	1	1	2	2	1	1	1	1	1	2	1	1	2	2	1	1	2	1	1	2	2	2	1	2	2	1	1	1	1	1	1
78	Primary Care Physician	KOR	6 - 10 years	30-39 patients	4	1	2	1	1	1	1	1	1	1	1	1	1	1	1	1	1	2	1	2	2	1	1	2	1	1	1	1	2	1	1	1	1	1	1	1	1	1	1	1	1	1
79	Consultant Endocrinologist	BRA	More than 10 years	30-39 patients	1	1	2	1	1	1	1	1	2	1	1	1	2	1	1	1	1	1	1	1	1	2	4	2	1	4	1	1	1	1	1	1	2	2	1	1	1	1	1	1	1	1
80	Primary Care Physician	KOR	More than 10 years	100+ patients	1	2	2	1	1	2	2	2	1	1	2	2	1	1	1	2	2	1	1	1	2	1	1	2	2	2	2	1	1	2	2	1	1	1	1	2	2	2	2	2	1	1
81	Primary Care Physician	ARG	More than 10 years	10-19 patients	1	2	2	2	2	2	2	2	2	2	2	3	2	3	1	2	2	2	2	2	2	2	3	2	2	1	2	2	2	1	3	2	2	2	1	1	2	1	2	2	1	2
82	Consultant Cardiologist	EGY	Less than 5 Years	80-89 patients	1	2	2	2	2	2	2	2	2	1	1	1	1	2	2	1	1	1	1	2	2	2	2	2	2	1	1	3	2	2	1	2	2	2	2	2	3	2	2	2	1	2
83	Primary Care Physician	SGP	More than 10 years	100+ patients	1	1	1	1	1	1	2	2	1	2	1	1	2	1	2	1	2	1	1	2	2	2	2	2	2	1	1	3	1	2	1	2	2	2	1	1	1	1	2	1	1	1
84	Primary Care Physician	ARG	More than 10 years	30-39 patients	1	1	2	1	1	1	2	1	2	1	1	1	1	2	1	1	1	2	1	2	1	2	4	2	4	1	1	4	2	1	1	1	1	1	2	1	1	1	1	1	1	1
85	Primary Care Physician	SGP	More than 10 years	100+ patients	1	1	1	1	2	2	2	1	1	2	1	1	2	2	1	1	1	1	1	1	2	2	2	2	2	2	1	2	1	2	3	1	2	2	1	1	1	2	1	1	1	1
86	Primary Care Physician	KOR	More than 10 years	10-19 patients	3	3	4	3	2	3	4	3	4	2	1	2	3	2	2	2	2	3	2	2	2	2	2	3	1	2	2	2	2	2	3	2	2	1	2	2	2	3	2	2	2	2
87	Consultant Endocrinologist	ARG	6 - 10 years	50-59 patients	3	1	2	3	1	2	2	2	2	1	1	2	2	1	1	1	1	1	2	2	2	2	2	1	3	2	2	3	1	2	2	2	2	2	2	2	2	2	2	1	1	1
88	Primary Care Physician	ARG	More than 10 years	70-79 patients	2	1	1	1	1	1	2	2	2	2	1	2	1	2	2	1	2	2	2	3	3	2	3	2	3	2	1	2	2	1	2	1	2	2	2	1	1	1	2	2	1	2
89	Primary Care Physician	KOR	More than 10 years	40-49 patients	1	1	1	2	1	2	2	1	2	1	1	1	1	2	1	1	1	1	1	2	2	2	1	2	2	1	1	2	1	1	2	1	1	1	1	2	1	2	1	1	1	1
90	Consultant Endocrinologist	KOR	6 - 10 years	60-69 patients	2	2	1	2	2	2	2	2	1	2	2	2	1	1	3	2	2	1	1	2	1	2	3	2	2	2	2	2	1	1	2	2	2	3	1	2	2	1	1	2	2	2
91	Primary Care Physician	KOR	More than 10 years	30-39 patients	2	2	2	2	2	2	2	2	2	2	2	2	2	2	2	2	2	2	2	2	2	2	2	2	2	2	2	2	2	2	2	2	2	2	2	2	2	2	2	2	2	2
92	Primary Care Physician	KOR	More than 10 years	70-79 patients	1	1	1	1	1	1	2	2	1	2	1	1	2	1	1	2	2	1	1	1	2	2	1	1	1	1	1	2	2	2	1	1	1	2	2	2	1	2	2	2	1	2
93	Consultant Cardiologist	MEX	6 - 10 years	10-19 patients	1	1	1	1	2	2	2	2	2	1	1	3	2	3	2	1	2	2	2	3	3	2	2	1	2	2	2	2	1	1	1	1	2	2	2	1	1	1	1	1	2	2
94	Primary Care Physician	ARG	6 - 10 years	10-19 patients	2	2	2	1	1	1	1	1	2	1	1	1	1	2	1	1	1	2	2	2	2	2	4	2	3	2	2	2	2	2	3	2	2	2	2	2	2	2	2	2	2	2
95	Consultant Endocrinologist	AUS	More than 10 years	50-59 patients	1	2	2	1	1	1	2	1	1	2	1	1	1	2	1	1	1	1	1	1	2	1	1	2	2	1	1	2	1	1	2	1	1	1	1	2	2	2	2	2	2	2
96	Consultant Cardiologist	SGP	6 - 10 years	100+ patients	1	1	1	1	1	1	1	1	2	1	1	1	1	1	1	1	1	1	1	1	1	1	1	2	2	1	1	2	1	1	1	1	1	2	2	2	1	1	1	1	1	2
97	Consultant Endocrinologist	SGP	6 - 10 years	100+ patients	1	1	1	1	1	1	2	1	2	2	1	1	2	2	1	1	1	1	1	1	2	2	2	2	2	2	1	3	2	1	1	1	2	2	1	1	2	2	1	1	1	1
98	Consultant Nephrologist	SGP	6 - 10 years	100+ patients	1	1	1	1	1	2	2	1	1	1	1	1	1	2	1	1	1	1	1	2	1	1	1	2	2	1	1	2	1	1	1	2	3	2	1	1	1	1	1	1	1	1
99	Consultant Nephrologist	SGP	More than 10 years	100+ patients	1	2	2	2	2	1	3	2	2	1	1	1	2	3	2	2	1	1	1	2	1	2	2	1	2	1	2	2	2	1	2	2	2	1	1	1	1	1	1	1	1	1
100	Consultant Endocrinologist	SGP	More than 10 years	100+ patients	1	1	1	2	1	1	1	1	1	1	1	2	2	1	1	1	1	1	2	2	2	2	1	1	1	1	1	2	1	1	1	1	1	2	1	2	1	1	1	2	1	1
101	Primary Care Physician	KOR	More than 10 years	50-59 patients	1	1	1	1	1	2	2	2	2	1	1	1	1	1	1	1	1	1	1	1	2	2	2	2	2	1	1	3	2	2	2	2	2	2	3	2	2	2	2	2	2	2
102	Consultant Nephrologist	TUR	More than 10 years	40-49 patients	1	1	2	2	1	1	2	1	1	2	1	2	1	2	2	1	2	1	2	2	2	3	2	2	2	2	2	2	2	2	2	1	1	2	2	1	1	1	1	1	1	1
103	Consultant Nephrologist	TUR	More than 10 years	40-49 patients	1	1	2	1	1	1	2	1	1	1	1	1	1	1	1	1	1	2	1	2	2	2	2	1	2	2	1	2	1	1	1	1	1	1	1	1	1	1	1	1	1	1
104	Consultant Endocrinologist	AUS	More than 10 years	70-79 patients	1	1	1	1	1	1	2	2	2	1	1	2	2	3	2	1	1	2	2	2	2	2	2	2	3	2	1	2	2	1	1	2	2	2	2	2	2	2	2	2	2	2
105	Primary Care Physician	SGP	More than 10 years	100+ patients	1	1	1	1	1	1	2	1	2	1	1	2	2	2	1	1	1	1	1	2	2	1	1	2	4	1	1	2	1	1	1	1	2	3	2	2	1	1	1	1	1	1
106	Consultant Nephrologist	SGP	6 - 10 years	100+ patients	1	1	1	1	3	2	2	2	1	1	1	1	1	1	1	1	1	1	1	2	2	1	2	2	2	1	1	2	2	1	1	2	1	2	1	1	3	2	1	1	1	1
107	Consultant Nephrologist	TUR	More than 10 years	40-49 patients	1	1	1	1	2	2	1	1	1	1	1	1	2	1	2	1	2	1	2	1	1	2	1	2	1	1	1	1	1	1	2	1	1	2	1	1	1	1	1	1	1	1
108	Primary Care Physician	SGP	More than 10 years	10-19 patients	1	2	1	1	2	2	1	2	2	2	1	2	2	2	1	2	1	1	1	1	2	2	1	2	2	2	1	2	1	1	1	1	1	2	1	2	1	1	1	1	1	1
109	Consultant Nephrologist	BRA	More than 10 years	100+ patients	1	2	2	2	1	1	1	1	2	1	1	1	1	1	1	1	1	1	1	1	1	1	2	2	3	2	1	2	2	2	2	2	2	3	2	2	2	2	2	1	1	1
110	Primary Care Physician	ARG	Less than 5 Years	20-29 patients	2	1	2	2	1	1	2	2	2	2	1	1	1	2	1	1	2	2	2	2	3	2	2	2	3	4	2	2	2	1	1	1	2	1	1	1	1	2	2	1	2	2
111	Consultant Endocrinologist	TUR	More than 10 years	60-69 patients	1	1	1	1	1	1	1	1	1	1	1	1	1	1	1	1	1	2	2	2	3	2	2	1	1	1	1	2	2	2	2	1	1	1	2	1	2	1	1	1	2	2
112	Consultant Cardiologist	ARG	More than 10 years	60-69 patients	1	1	1	1	1	1	1	1	1	1	1	1	1	1	1	1	1	1	1	1	2	2	1	2	2	1	1	2	1	1	1	1	2	2	1	1	1	1	1	1	1	1
113	Consultant Endocrinologist	MEX	More than 10 years	50-59 patients	1	3	1	1	1	1	2	1	2	1	1	1	1	1	1	1	1	1	1	1	1	3	1	3	2	1	1	2	1	1	1	1	1	2	1	1	1	1	1	1	1	1
114	Consultant Cardiologist	SGP	6 - 10 years	90-99 patients	3	1	1	1	1	1	2	2	2	1	1	2	2	3	1	1	1	1	1	1	1	1	1	2	3	1	1	1	1	1	1	1	1	2	1	2	1	2	1	1	1	1
115	Primary Care Physician	SGP	More than 10 years	10-19 patients	1	2	2	1	2	2	2	1	3	2	1	1	2	2	1	1	2	2	2	2	2	3	3	3	1	1	1	2	2	1	1	1	2	2	2	2	1	1	1	1	1	1
116	Primary Care Physician	SGP	More than 10 years	100+ patients	1	1	1	1	2	1	1	1	2	1	1	1	1	2	2	1	1	2	1	1	1	1	2	2	2	1	1	1	2	1	2	1	2	2	1	1	1	1	2	1	1	1
117	Primary Care Physician	KOR	More than 10 years	80-89 patients	1	2	2	2	2	1	2	2	2	2	1	2	2	2	2	2	2	1	1	1	2	2	2	2	2	2	2	2	2	2	2	2	2	2	2	2	2	2	2	2	2	2
118	Consultant Cardiologist	MEX	More than 10 years	10-19 patients	1	1	1	1	1	1	1	1	1	1	1	1	1	1	1	1	1	1	1	1	1	1	1	1	1	1	1	1	1	1	1	1	1	1	1	1	1	1	1	1	1	1
119	Consultant Endocrinologist	SGP	More than 10 years	70-79 patients	1	1	1	2	1	2	2	1	1	1	1	2	1	2	1	1	1	1	1	2	2	2	2	2	2	1	1	2	1	2	1	1	1	3	1	2	2	2	2	1	1	1
120	Consultant Nephrologist	SGP	6 - 10 years	100+ patients	1	1	1	1	1	1	1	1	1	1	1	1	2	1	1	1	1	1	1	2	2	1	1	1	2	1	1	2	1	2	1	1	1	3	1	2	1	2	1	1	1	2
121	Consultant Cardiologist	SGP	More than 10 years	100+ patients	2	1	1	1	1	1	1	1	1	1	1	1	1	2	1	1	1	1	1	1	1	1	1	2	2	1	1	2	1	1	1	1	1	1	1	1	1	1	1	2	2	2
122	Consultant Cardiologist	SGP	More than 10 years	40-49 patients	1	2	1	1	1	1	3	1	2	1	1	2	2	2	1	1	1	2	1	1	1	1	2	2	2	1	1	1	1	1	1	1	2	2	1	2	2	2	2	1	1	1
123	Primary Care Physician	SGP	6 - 10 years	100+ patients	1	1	1	1	1	1	2	2	2	1	2	1	1	2	1	1	1	1	1	2	2	3	2	2	1	3	2	1	1	1	1	1	1	2	1	1	1	1	1	1	1	1
124	Consultant Nephrologist	TUR	6 - 10 years	100+ patients	1	1	1	1	1	1	1	1	1	1	1	1	2	1	1	1	1	1	1	1	1	2	3	2	1	1	1	1	1	3	1	1	2	1	2	1	2	2	1	1	1	1
125	Primary Care Physician	TWN	More than 10 years	40-49 patients	1	2	1	2	1	1	2	1	2	1	2	2	1	2	1	1	1	1	1	1	2	2	2	1	2	1	1	2	1	1	1	1	2	1	1	1	1	2	2	2	1	1
126	Primary Care Physician	TWN	More than 10 years	50-59 patients	1	2	2	2	1	1	2	2	2	1	2	2	1	2	1	1	2	1	1	1	2	2	2	2	2	1	1	3	2	1	2	2	1	2	2	1	1	2	2	1	2	1
127	Primary Care Physician	TWN	More than 10 years	100+ patients	1	1	2	2	1	1	2	2	2	1	1	2	1	1	2	2	2	2	1	1	2	2	3	2	2	1	1	2	1	1	2	2	2	1	2	2	1	2	2	2	1	2
128	Primary Care Physician	TWN	More than 10 years	80-89 patients	1	1	1	1	1	1	1	1	2	1	1	1	1	2	1	1	1	2	2	2	1	2	3	2	2	1	1	2	2	1	2	1	1	1	1	2	2	1	1	1	2	1
129	Primary Care Physician	TWN	More than 10 years	100+ patients	1	1	2	2	1	1	2	2	1	1	1	1	2	2	1	1	1	1	1	2	2	1	3	2	2	1	1	3	2	1	1	2	1	2	1	1	1	2	2	2	1	1
130	Primary Care Physician	TWN	More than 10 years	100+ patients	1	1	2	2	1	1	2	2	3	1	2	2	2	2	2	1	1	2	1	1	1	2	2	2	2	2	1	2	2	1	2	1	2	2	1	2	2	2	1	2	1	1
131	Primary Care Physician	TWN	More than 10 years	40-49 patients	1	1	1	1	1	2	2	1	2	1	1	1	2	2	1	1	1	1	1	2	2	2	2	1	2	1	2	2	1	1	2	1	2	2	1	1	1	1	1	1	2	1
132	Primary Care Physician	TWN	More than 10 years	10-19 patients	1	2	2	1	2	2	2	2	1	2	2	1	2	2	2	2	2	2	1	2	2	2	2	2	2	1	2	2	2	1	2	2	2	2	2	2	2	1	1	2	1	1
133	Consultant Endocrinologist	TWN	More than 10 years	100+ patients	1	1	2	1	2	1	3	2	2	1	2	2	1	2	2	2	1	2	1	2	2	3	2	2	3	1	2	2	2	1	1	1	3	2	2	2	1	1	2	2	1	2
134	Consultant Endocrinologist	TWN	More than 10 years	100+ patients	2	2	3	2	1	1	2	2	2	2	2	2	1	3	2	2	2	1	1	1	2	2	2	2	2	1	2	3	1	1	2	2	1	2	2	1	1	2	2	2	1	2
135	Consultant Endocrinologist	TWN	More than 10 years	100+ patients	1	1	1	1	1	1	4	2	1	1	2	1	1	3	1	1	1	1	1	1	1	3	2	2	4	2	1	1	3	1	2	2	2	4	1	1	3	1	1	1	1	1
136	Consultant Endocrinologist	TWN	More than 10 years	100+ patients	1	1	2	2	1	1	3	1	2	2	1	1	2	3	1	2	2	1	1	1	1	3	2	3	3	2	1	4	2	1	1	1	2	3	2	1	1	2	3	1	1	2

137	Consultant Endocrinologist	TWN	More than 10 years	100+ patients	1	2	1	1	2	1	2	2	2	1	2	2	2	3	1	1	2	1	1	1	2	2	1	2	3	2	1	3	2	2	2	1	3	3	1	2	1	2	1	1	1	2
138	Consultant Cardiologist	TWN	More than 10 years	80-89 patients	1	1	2	1	2	1	2	2	2	2	1	2	2	2	2	2	1	2	1	2	2	2	2	2	2	1	1	2	1	2	2	2	2	1	1	2	1	2	2	2	2	2
139	Consultant Cardiologist	TWN	More than 10 years	60-69 patients	1	1	2	1	1	2	1	2	2	1	1	1	1	3	2	1	1	1	1	1	2	1	2	2	2	2	1	2	1	2	2	2	2	3	1	2	1	1	1	1	2	2
140	Consultant Cardiologist	TWN	More than 10 years	100+ patients	1	1	1	1	1	1	1	1	1	1	1	1	1	1	1	1	1	1	1	1	1	1	2	2	1	1	1	2	1	1	1	1	1	2	1	1	1	1	1	1	1	1
141	Consultant Cardiologist	TWN	More than 10 years	50-59 patients	1	2	1	1	1	1	2	2	2	1	1	1	3	3	1	1	1	3	1	1	1	1	2	2	3	1	1	1	1	1	1	1	1	2	1	1	1	1	1	1	1	1
142	Consultant Cardiologist	TWN	More than 10 years	50-59 patients	1	2	1	2	1	1	3	2	3	1	1	2	1	2	2	1	2	1	1	1	1	2	2	2	2	1	1	2	2	1	1	2	2	1	3	3	2	1	2	1	2	1
143	Consultant Nephrologist	TWN	More than 10 years	100+ patients	1	3	1	1	1	1	1	1	1	1	1	2	2	3	1	1	1	2	1	1	1	2	2	3	4	2	1	2	2	1	1	2	1	2	1	2	2	2	1	1	1	1
144	Consultant Nephrologist	TWN	More than 10 years	100+ patients	1	2	2	2	1	1	1	2	1	1	1	1	2	2	1	2	2	2	2	2	2	2	2	2	3	2	1	1	2	1	2	1	2	2	2	2	3	2	2	3	2	2
145	Consultant Nephrologist	TWN	More than 10 years	100+ patients	2	1	1	1	1	1	2	1	1	1	1	1	1	2	1	1	1	1	1	2	1	2	2	1	2	1	1	2	2	1	1	2	1	2	3	1	2	2	1	2	1	1
146	Primary Care Physician	THA	More than 10 years	10-19 patients	1	1	1	1	1	1	1	1	2	2	1	1	1	2	1	1	1	2	2	2	2	2	3	1	1	1	1	3	1	1	1	1	1	1	1	1	1	1	1	1	1	1
147	Primary Care Physician	THA	6 - 10 years	40-49 patients	2	2	2	2	2	2	2	2	2	2	2	2	2	2	2	1	2	2	2	2	2	2	2	2	2	2	2	1	2	2	2	2	1	2	2	2	2	2	1	2	2	2
148	Primary Care Physician	THA	Less than 5 Years	40-49 patients	1	1	1	1	1	1	1	1	1	1	1	1	2	2	2	1	1	2	2	3	2	2	2	2	2	2	1	2	2	1	1	1	2	2	1	1	1	1	1	1	1	1
149	Primary Care Physician	TWN	More than 10 years	100+ patients	1	1	4	1	1	1	4	1	2	2	1	1	1	2	1	1	1	1	1	1	1	1	1	2	2	2	1	1	1	1	1	1	2	2	3	1	1	1	1	1	1	1
150	Primary Care Physician	SGP	More than 10 years	50-59 patients	1	1	1	1	1	1	2	1	2	1	1	1	2	2	1	1	1	1	1	1	1	1	2	1	2	1	1	3	1	1	1	2	1	2	1	2	1	1	1	1	1	1
151	Primary Care Physician	SGP	More than 10 years	30-39 patients	1	2	2	2	2	2	2	2	2	2	2	2	2	2	1	1	2	2	2	2	2	2	3	2	2	1	1	3	1	2	1	1	1	2	1	2	2	2	1	1	2	1
152	Consultant Endocrinologist	ARG	More than 10 years	40-49 patients	3	2	3	1	1	1	2	2	1	1	1	2	1	1	2	1	2	1	1	2	2	1	1	1	1	1	1	1	2	1	1	1	1	1	1	1	1	2	1	2	1	1
153	Consultant Endocrinologist	KOR	More than 10 years	20-29 patients	1	2	1	2	2	2	3	2	3	2	2	1	2	2	2	1	2	2	2	3	2	2	2	2	2	1	1	2	2	2	2	2	3	2	2	2	2	2	2	1	1	1
154	Consultant Cardiologist	KOR	More than 10 years	20-29 patients	1	1	1	1	1	1	1	1	1	1	1	1	1	1	1	1	1	1	1	1	3	1	3	2	2	1	1	3	2	1	1	1	1	2	1	1	1	1	1	1	1	1
155	Consultant Endocrinologist	KOR	More than 10 years	100+ patients	1	1	1	1	1	1	1	1	1	1	1	1	1	1	1	1	1	1	1	1	1	1	1	1	1	1	1	3	1	1	1	1	1	2	1	1	1	1	1	1	1	1
156	Consultant Endocrinologist	THA	More than 10 years	100+ patients	2	1	1	1	1	1	1	1	1	1	2	2	2	2	1	1	1	1	1	2	2	2	2	2	2	2	1	3	1	2	1	1	1	1	1	1	1	1	1	1	1	1
157	Consultant Nephrologist	KOR	More than 10 years	100+ patients	1	1	1	1	1	1	1	1	1	1	1	1	1	1	1	1	1	1	1	1	1	1	1	1	3	1	1	1	2	1	1	1	1	2	2	1	1	1	1	1	1	1
158	Primary Care Physician	KOR	Less than 5 Years	100+ patients	2	2	2	2	2	2	2	2	2	2	2	2	1	2	2	2	2	2	1	2	2	2	2	2	2	2	2	3	2	2	2	2	2	2	2	2	2	2	2	2	2	2
159	Consultant Endocrinologist	KOR	6 - 10 years	50-59 patients	1	2	2	1	1	1	2	1	1	1	1	1	1	1	1	1	1	1	1	3	2	3	2	2	2	1	3	2	2	2	2	2	3	2	2	2	2	2	2	1	1	1
160	Primary Care Physician	THA	Less than 5 Years	60-69 patients	1	2	2	1	1	1	2	1	2	1	1	1	2	3	2	1	3	2	1	2	1	2	2	2	3	1	1	3	1	1	1	1	1	1	1	1	1	2	1	1	1	1
161	Consultant Nephrologist	TUR	6 - 10 years	100+ patients	1	1	1	1	1	1	1	1	1	1	1	1	1	1	1	1	1	1	1	1	1	1	1	2	2	1	1	4	1	2	2	1	1	1	2	1	2	1	1	1	1	1
162	Consultant Endocrinologist	THA	More than 10 years	100+ patients	1	1	1	1	1	1	1	1	1	1	1	1	1	1	1	1	1	1	1	3	2	2	2	2	2	2	1	1	1	1	1	1	1	1	1	1	2	2	2	1	1	1
163	Primary Care Physician	THA	Less than 5 Years	40-49 patients	1	1	1	1	2	1	1	1	2	1	2	1	2	3	1	1	1	1	2	3	2	2	4	3	3	2	1	2	1	1	1	1	1	1	1	1	1	1	1	1	1	1
164	Consultant Nephrologist	THA	More than 10 years	100+ patients	1	1	1	1	1	1	1	2	1	1	1	1	1	1	1	1	1	1	1	1	2	2	1	2	1	2	1	3	1	1	1	1	1	1	1	2	2	1	1	1	1	1
165	Consultant Nephrologist	TWN	More than 10 years	100+ patients	2	1	1	1	1	1	2	2	1	1	1	1	2	3	1	1	1	1	1	1	1	1	1	2	2	1	1	2	1	1	1	1	1	1	1	1	2	1	1	1	1	1
166	Consultant Nephrologist	TWN	More than 10 years	100+ patients	1	1	1	1	1	1	1	1	1	2	2	1	1	1	2	1	1	1	1	1	2	2	1	2	2	1	1	3	2	2	2	1	2	2	2	2	2	1	1	2	2	1
167	Consultant Cardiologist	ARG	6 - 10 years	10-19 patients	2	1	1	1	1	1	1	2	2	1	1	2	2	3	2	2	2	2	1	2	3	2	3	2	1	1	1	3	1	2	1	2	3	4	2	1	1	2	2	2	1	1
168	Consultant Endocrinologist	THA	More than 10 years	50-59 patients	1	1	1	1	1	1	2	2	2	1	1	2	3	2	1	1	2	1	2	2	3	3	3	2	3	2	2	3	2	2	2	2	2	1	1	2	2	2	1	1	1	1
169	Consultant Cardiologist	THA	More than 10 years	90-99 patients	1	1	1	1	1	1	1	1	1	1	1	1	1	2	2	1	1	1	1	1	2	2	2	1	1	2	1	3	1	1	2	1	1	1	2	1	2	1	1	1	1	1
170	Consultant Cardiologist	THA	More than 10 years	20-29 patients	1	1	1	1	1	1	1	1	2	1	1	1	1	1	1	1	1	1	1	1	2	2	2	1	2	1	1	1	1	1	1	1	1	1	1	1	2	1	1	1	1	1
171	Consultant Nephrologist	MEX	More than 10 years	80-89 patients	4	1	1	1	1	1	1	2	2	1	1	1	1	1	1	1	1	1	1	1	1	1	1	1	2	1	1	1	1	1	1	1	1	2	1	1	1	1	1	1	2	1
172	Consultant Nephrologist	BRA	Less than 5 Years	100+ patients	1	1	1	1	1	1	1	1	1	1	1	1	1	1	1	1	1	1	1	1	1	1	1	1	3	2	3	2	1	1	1	1	1	1	1	1	1	1	1	1	1	1
173	Consultant Cardiologist	ARG	More than 10 years	50-59 patients	2	3	1	1	1	1	1	2	1	1	1	2	1	2	2	2	1	1	1	2	1	2	2	2	3	2	1	2	1	2	2	1	2	3	1	1	1	2	3	2	1	1
174	Consultant Nephrologist	ARG	6 - 10 years	100+ patients	1	1	1	1	1	1	2	1	1	1	1	1	1	1	1	1	1	1	1	1	1	1	1	1	2	1	1	1	1	1	1	1	1	1	1	1	1	1	1	1	1	1
175	Consultant Nephrologist	ARG	6 - 10 years	20-29 patients	4	4	1	2	1	1	1	1	1	1	1	2	2	2	1	1	1	2	2	2	2	2	1	1	2	1	1	3	1	2	1	1	2	2	1	1	1	2	1	1	1	1
176	Consultant Cardiologist	ARG	Less than 5 Years	10-19 patients	3	1	1	1	1	2	2	1	2	2	1	2	2	2	1	1	2	2	2	2	2	2	1	2	2	2	1	2	2	2	2	2	3	2	1	1	2	2	2	2	2	2
177	Primary Care Physician	ARG	Less than 5 Years	20-29 patients	1	1	1	1	1	2	2	2	2	1	1	1	1	2	1	1	1	2	2	2	2	2	3	2	3	2	1	2	1	1	1	1	1	1	1	1	1	1	1	1	1	1
178	Consultant Cardiologist	ARG	More than 10 years	60-69 patients	1	1	1	1	1	1	2	1	2	1	1	1	1	1	1	1	1	2	1	1	1	1	1	2	2	3	1	2	1	1	1	1	1	1	1	1	1	1	1	1	1	1
179	Primary Care Physician	GTM	More than 10 years	100+ patients	1	1	1	1	1	1	1	1	1	1	1	1	1	1	1	1	1	1	1	1	2	2	2	2	2	3	1	1	1	1	1	1	1	1	1	1	1	1	1	1	1	1
180	Consultant Endocrinologist	GTM	Less than 5 Years	20-29 patients	1	1	1	1	1	1	1	1	1	1	1	1	1	1	1	1	1	1	1	1	1	1	1	1	1	1	1	2	1	1	1	1	1	1	1	1	1	1	2	1	1	1
181	Consultant Nephrologist	ARG	6 - 10 years	100+ patients	1	1	1	1	1	1	1	2	1	1	1	1	1	1	1	1	1	1	1	1	1	3	1	3	1	4	1	1	1	1	1	1	1	1	1	1	1	1	1	1	1	1
182	Consultant Nephrologist	ARG	6 - 10 years	50-59 patients	1	2	2	2	1	1	1	3	2	1	1	1	2	2	2	1	1	2	2	2	1	1	2	2	3	2	2	2	2	1	2	2	2	2	1	1	1	2	2	1	1	2
183	Consultant Endocrinologist	ARG	6 - 10 years	80-89 patients	1	2	1	1	1	1	1	2	1	1	1	1	1	1	1	1	1	1	1	1	1	2	2	1	2	1	1	2	1	1	1	1	1	1	1	1	1	1	1	1	1	1
184	Consultant Nephrologist	KOR	More than 10 years	100+ patients	1	1	2	1	1	2	2	1	3	2	1	1	2	3	1	2	1	1	1	2	2	2	2	2	3	2	1	2	1	2	2	2	2	2	2	1	2	3	2	1	2	2
185	Consultant Endocrinologist	GTM	More than 10 years	30-39 patients	1	1	1	1	1	1	1	1	1	1	1	1	2	2	1	1	1	1	1	1	1	2	2	1	2	1	1	3	1	1	1	1	1	3	1	1	1	1	1	1	1	1
186	Consultant Endocrinologist	ARG	Less than 5 Years	30-39 patients	1	1	1	1	1	1	2	1	1	1	1	1	2	2	1	1	1	1	1	2	1	2	1	2	2	1	1	2	1	1	2	1	2	3	1	1	1	2	2	2	1	1
187	Consultant Endocrinologist	ARG	Less than 5 Years	50-59 patients	1	1	1	1	1	1	1	1	1	1	1	1	3	3	1	1	1	1	1	1	1	1	1	1	3	3	1	2	1	1	1	1	1	1	1	1	1	1	1	1	1	1
188	Consultant Cardiologist	KOR	More than 10 years	100+ patients	1	1	1	1	1	1	2	1	2	1	1	1	2	2	1	1	1	1	1	1	1	3	2	2	2	1	1	3	2	2	1	2	2	1	2	2	2	2	1	1	2	2
189	Primary Care Physician	ARG	More than 10 years	20-29 patients	1	1	1	1	1	1	2	2	2	1	2	2	2	3	1	1	2	2	1	2	2	2	2	2	2	1	1	2	1	1	1	1	1	1	1	1	1	1	1	1	1	1
190	Consultant Nephrologist	ARG	6 - 10 years	100+ patients	1	1	1	1	1	1	1	1	1	1	1	2	1	2	1	1	1	1	1	1	1	1	1	1	3	2	1	1	1	1	1	1	1	1	1	1	1	1	1	1	1	1
191	Primary Care Physician	ARG	More than 10 years	20-29 patients	1	1	1	1	1	1	2	1	1	1	2	1	2	2	1	1	2	2	2	2	2	2	2	2	2	1	1	1	1	1	1	1	1	2	1	2	1	2	1	1	1	1
192	Consultant Nephrologist	ARG	More than 10 years	100+ patients	1	1	2	1	1	2	2	2	2	1	1	1	3	3	1	1	1	1	1	2	1	1	2	2	2	2	1	2	1	3	3	1	2	2	1	1	3	2	3	1	2	1
193	Consultant Endocrinologist	SGP	More than 10 years	90-99 patients	1	1	1	1	1	1	1	1	2	1	1	2	2	2	1	1	1	1	1	1	1	3	2	2	2	2	1	2	1	1	1	1	1	1	1	1	1	1	1	1	1	1
194	Consultant Endocrinologist	SGP	More than 10 years	100+ patients	1	1	1	1	1	1	2	3	1	1	1	1	1	1	1	1	1	2	1	1	1	2	1	2	1	2	1	1	1	1	1	1	1	1	1	1	1	1	1	1	1	1
195	Consultant Cardiologist	SGP	More than 10 years	100+ patients	1	1	2	1	1	1	2	2	2	1	1	1	1	2	1	1	1	1	1	1	1	2	1	1	1	2	1	1	2	1	1	2	2	1	2	1	1	1	1	1	1	1
196	Primary Care Physician	KOR	More than 10 years	50-59 patients	1	2	2	2	1	1	2	1	2	2	1	1	2	2	1	2	1	1	1	2	2	2	2	2	2	1	1	2	2	1	2	2	2	2	1	2	2	2	1	2	1	1
197	Consultant Nephrologist	THA	More than 10 years	100+ patients	1	1	1	1	1	1	1	1	1	1	1	1	1	1	1	1	1	1	1	1	1	1	1	1	1	2	1	3	1	1	1	1	1	1	1	1	1	1	1	1	1	1
198	Consultant Nephrologist	THA	6 - 10 years	100+ patients	1	1	1	1	1	1	2	1	1	1	1	1	1	1	1	1	1	1	1	1	1	1	4	1	1	1	1	1	1	1	1	1	1	1	1	1	1	1	1	1	1	1
199	Consultant Nephrologist	KOR	More than 10 years	100+ patients	1	1	1	1	1	1	2	1	1	2	1	1	1	1	1	1	2	1	1	2	2	2	2	2	2	1	1	3	2	2	2	2	2	2	1	2	2	2	1	1	2	2
200	Primary Care Physician	SGP	More than 10 years	100+ patients	1	2	2	1	2	1	2	1	1	1	1	1	1	1	1	1	1	1	1	2	1	1	1	2	3	2	1	3	2	2	1	1	1	2	2	1	2	1	1	1	1	1
201	Consultant Cardiologist	THA	More than 10 years	80-89 patients	1	2	1	2	1	1	1	2	1	1	2	2	2	1	1	1	1	1	2	1	1	2	1	1	2	2	1	1	1	2	2	2	1	2	1	1	1	1	1	1	2	1
202	Primary Care Physician	GTM	More than 10 years	10-19 patients	2	3	2	2	2	1	2	2	2	2	1	2	2	2	1	1	2	2	2	2	2	2	2	2	2	3	1	2	2	2	1	1	2	2	1	1	1	1	1	1	1	1
203	Primary Care Physician	GTM	Less than 5 Years	10-19 patients	4	1	2	1	1	1	2	2	3	1	2	1	2	2	1	1	1	2	1	2	1	1	2	2	3	1	1	1	1	1	1	1	1	2	1	1	1	1	1	1	1	1
204	Primary Care Physician	GTM	6 - 10 years	20-29 patients	2	1	1	2	1	1	1	1	1	1	1	2	1	2	2	1	2	2	2	2	2	2	2	2	3	2	1	2	1	1	1	1	1	2	1	1	1	1	1	1	1	1
205	Primary Care Physician	GTM	6 - 10 years	30-39 patients	1	1	1	1	1	1	1	1	2	1	1	2	1	1	1	1	1	1	1	1	2	2	2	2	2	2	1	2	1	1	1	1	1	2	1	1	1	1	1	1	1	1
206	Primary Care Physician	GTM	More than 10 years	80-89 patients	1	1	1	1	1	1	1	1	1	1	1	1	1	1	1	1	1	1	1	1	1	1	1	1	3	1	1	1	1	1	1	1	1	1	1	1	1	1	1	1	1	1
207	Primary Care Physician	GTM	Less than 5 Years	10-19 patients	1	1	1	1	1	1	1	1	1	1	1	1	1	2	1	1	1	1	1	1	1	1	2	1	4	1	1	3	1	1	1	1	1	2	1	1	1	2	1	1	1	1
208	Primary Care Physician	GTM	6 - 10 years	60-69 patients	1	1	1	1	1	1	2	1	2	1	1	1	2	2	1	1	2	1	2	2	2	1	2	2	2	1	1	1	1	2	1	1	1	1	1	1	1	1	1	1	1	1
209	Consultant Nephrologist	KOR	6 - 10 years	100+ patients	2	1	2	2	2	2	2	3	2	2	2	2	2	3	2	2	2	2	2	2	2	2	2	2	3	2	2	2	3	2	2	3	3	3	2	3	2	3	3	2	2	2
210	Primary Care Physician	GTM	Less than 5 Years	10-19 patients	2	1	1	1	1	1	2	1	1	1	1	1	1	1	1	1	1	1	1	1	1	2	1	1	4	1	1	1	1	1	1	1	1	2	1	1	1	1	1	1	1	1
211	Consultant Nephrologist	MEX	More than 10 years	100+ patients	1	1	1	1	1	1	2	2	1	1	1	1	1	1	1	1	1	1	1	2	1	2	2	2	2	2	1	2	1	1	2	1	1	1	1	1	1	2	1	1	1	1
212	Primary Care Physician	GTM	6 - 10 years	10-19 patients	1	1	2	2	1	1	1	1	2	1	1	2	1	1	1	1	1	2	1	2	2	2	2	2	3	1	2	2	2	2	1	1	1	3	1	1	1	1	1	1	1	1
213	Primary Care Physician	GTM	More than 10 years	10-19 patients	1	1	1	2	1	1	1	2	2	1	2	2	3	1	1	1	1	1	1	3	2	1	2	1	2	1	1	1	1	1	1	1	1	2	1	1	1	1	1	1	1	1
214	Consultant Nephrologist	EGY	More than 10 years	80-89 patients	1	1	1	2	2	1	2	1	1	2	2	3	2	3	1	1	1	1	2	2	2	2	1	2	2	2	2	2	1	1	1	1	3	2	4	2	1	1	1	1	1	1
215	Primary Care Physician	GTM	More than 10 years	10-19 patients	1	1	2	1	1	1	2	2	3	1	2	1	2	2	1	1	2	2	2	3	3	2	1	1	3	2	2	3	1	1	1	2	2	2	1	1	1	1	1	1	1	1
216	Primary Care Physician	GTM	6 - 10 years	40-49 patients	1	1	1	1	1	1	1	1	1	1	1	1	1	1	1	1	1	1	1	1	1	1	2	2	2	2	1	3	1	1	1	1	1	1	1	1	1	1	1	1	1	1
217	Consultant Cardiologist	KOR	More than 10 years	100+ patients	1	1	1	1	1	2	1	2	3	1	1	1	1	2	2	1	1	2	2	2	2	1	2	3	2	1	1	3	2	2	1	2	1	3	1	2	2	2	1	2	2	1
218	Primary Care Physician	GTM	Less than 5 Years	10-19 patients	4	2	2	1	1	1	3	1	4	1	1	1	1	1	1	1	1	2	2	2	2	3	3	2	4	1	1	3	1	1	1	1	1	1	1	1	1	1	1	2	1	1
219	Primary Care Physician	GTM	More than 10 years	10-19 patients	1	1	2	1	1	3	2	1	1	1	1	2	1	3	2	1	2	2	2	4	2	1	4	2	1	1	1	1	1	2	1	1	1	2	1	2	1	1	1	1	1	1
220	Primary Care Physician	THA	More than 10 years	10-19 patients	1	1	1	1	1	1	2	2	2	1	1	2	2	2	2	2	2	2	2	2	2	2	3	2	3	2	1	2	2	2	2	2	2	2	1	1	1	2	2	1	1	1
221	Consultant Nephrologist	THA	6 - 10 years	100+ patients	1	1	1	1	1	1	2	2	1	1	1	1	1	1	1	1	1	1	1	1	1	1	1	2	1	1	1	2	1	1	1	1	1	1	1	1	1	1	1	1	1	1
222	Consultant Nephrologist	TUR	More than 10 years	80-89 patients	4	4	3	3	4	4	4	4	4	4	4	3	4	3	4	1	2	2	2	2	2	3	3	3	2	1	1	2	2	2	2	2	2	2	2	1	2	2	2	2	2	2
223	Consultant Nephrologist	THA	Less than 5 Years	100+ patients	1	1	1	1	1	1	1	1	1	1	1	1	1	1	1	1	1	1	1	1	2	1	1	1	1	2	1	3	1	1	1	1	1	1	1	1	1	1	1	1	1	1
224	Consultant Endocrinologist	THA	6 - 10 years	80-89 patients	2	1	1	1	1	1	2	1	1	1	2	1	1	1	1	1	1	1	2	1	2	2	2	2	2	1	1	3	1	2	1	1	1	1	1	1	1	1	1	1	1	1
225	Consultant Endocrinologist	THA	6 - 10 years	90-99 patients	1	1	1	1	1	1	2	2	1	1	1	1	2	2	1	1	2	1	1	2	2	2	3	2	2	2	1	3	1	1	2	1	1	1	1	2	2	2	1	1	1	1
226	Consultant Nephrologist	EGY	More than 10 years	80-89 patients	1	2	1	2	1	1	2	2	1	1	2	3	3	3	2	1	2	2	2	2	1	2	2	2	3	3	2	1	1	1	2	1	2	2	2	2	1	2	2	2	1	2
227	Consultant Nephrologist	SGP	6 - 10 years	100+ patients	1	1	2	1	1	1	2	1	1	1	1	1	1	2	1	1	1	1	2	1	1	2	2	1	1	1	1	2	2	1	1	1	1	1	1	2	2	2	1	1	1	1
228	Primary Care Physician	THA	More than 10 years	50-59 patients	1	1	1	1	1	1	2	1	1	1	1	1	1	2	1	1	1	1	2	1	2	1	3	2	2	2	1	2	1	1	1	1	1	1	1	1	1	1	1	1	1	1
229	Consultant Cardiologist	THA	More than 10 years	40-49 patients	2	2	1	2	1	1	3	2	2	2	1	2	2	3	1	1	2	2	2	3	2	2	2	2	3	1	2	2	2	2	2	2	1	1	1	2	2	1	2	2	2	1
230	Primary Care Physician	GTM	6 - 10 years	10-19 patients	4	4	3	4	4	4	3	3	2	1	2	2	3	3	1	1	2	2	2	2	2	2	2	2	2	1	1	3	1	2	1	1	1	2	1	1	1	1	1	1	1	1
231	Consultant Endocrinologist	GTM	More than 10 years	10-19 patients	1	1	1	1	1	1	1	1	1	1	1	1	1	2	1	1	1	1	1	1	1	2	1	2	1	1	1	1	1	1	1	1	1	1	1	1	1	1	1	1	1	1
232	Consultant Cardiologist	KOR	More than 10 years	100+ patients	1	2	1	2	1	1	3	2	2	2	1	1	1	1	2	2	2	2	3	3	3	3	2	2	2	1	2	2	2	2	2	1	3	3	2	2	2	2	2	2	1	3
233	Consultant Cardiologist	KOR	More than 10 years	90-99 patients	1	1	1	2	2	2	1	1	1	1	2	1	1	1	1	2	2	3	2	2	2	2	2	2	1	1	1	2	2	2	2	2	3	3	2	1	2	1	1	2	1	1
234	Primary Care Physician	THA	6 - 10 years	100+ patients	1	1	1	2	2	1	2	2	2	1	1	1	1	3	1	1	2	2	1	3	2	2	2	2	2	2	1	1	1	1	1	1	2	2	1	1	1	2	1	1	1	1
235	Consultant Cardiologist	GTM	6 - 10 years	50-59 patients	1	1	1	1	1	1	1	1	1	1	1	1	1	1	1	1	1	1	1	1	1	2	1	1	2	1	1	2	1	1	1	1	1	2	1	1	1	1	1	1	1	1
236	Consultant Cardiologist	THA	6 - 10 years	70-79 patients	1	1	1	2	1	1	1	2	1	1	2	2	1	1	2	1	1	1	2	1	1	2	2	1	2	2	1	3	1	2	2	2	1	2	1	1	2	2	1	1	1	2
237	Primary Care Physician	THA	6 - 10 years	50-59 patients	1	1	1	2	2	1	2	1	1	1	1	1	1	2	2	1	2	2	2	3	2	2	2	2	2	2	1	2	2	1	1	2	2	1	2	1	1	1	1	1	2	1
238	Primary Care Physician	AUS	More than 10 years	100+ patients	2	2	2	2	2	2	2	2	2	2	2	2	2	2	2	2	2	2	2	2	2	3	3	2	3	2	2	1	1	2	1	1	2	3	2	1	1	1	1	1	1	1
239	Primary Care Physician	AUS	6 - 10 years	30-39 patients	1	1	1	1	1	1	1	1	1	1	1	1	1	1	1	1	1	2	2	2	3	2	2	1	1	1	1	1	1	1	1	1	1	1	1	1	1	1	1	1	1	1
240	Primary Care Physician	AUS	More than 10 years	40-49 patients	4	1	1	1	1	1	2	2	2	1	1	2	1	1	1	1	2	2	2	2	2	2	3	2	3	2	1	2	1	1	1	1	1	1	1	1	1	1	1	1	1	1
241	Primary Care Physician	THA	Less than 5 Years	30-39 patients	1	1	2	1	1	1	2	1	2	2	1	1	2	2	2	1	1	2	2	2	2	2	3	2	1	1	1	3	1	1	1	1	1	2	1	1	1	2	1	1	1	1
242	Primary Care Physician	AUS	More than 10 years	80-89 patients	1	1	1	1	1	1	1	1	1	1	1	1	1	1	1	1	1	1	1	1	1	1	1	1	1	1	1	1	1	1	1	1	1	1	1	1	1	1	1	1	1	1
243	Consultant Cardiologist	GTM	More than 10 years	20-29 patients	1	1	1	1	1	1	1	1	1	1	1	1	2	2	1	1	2	1	1	1	1	1	1	1	3	3	1	1	1	1	1	1	1	3	1	1	2	1	1	1	1	1
244	Consultant Cardiologist	GTM	More than 10 years	20-29 patients	1	1	1	1	1	1	1	1	2	1	1	1	1	1	1	1	1	1	1	2	2	1	1	1	2	1	1	1	1	1	1	1	1	1	1	1	1	1	1	1	1	1
245	Consultant Nephrologist	GTM	6 - 10 years	100+ patients	1	1	1	1	1	1	1	1	2	1	1	1	1	1	1	1	1	1	1	1	1	1	1	1	4	1	1	4	1	1	1	1	1	1	1	1	1	1	1	1	1	1
246	Consultant Endocrinologist	GTM	6 - 10 years	30-39 patients	1	1	1	1	1	1	1	1	1	1	1	1	1	1	1	1	1	1	1	1	1	1	1	1	2	1	1	1	1	1	1	1	1	1	1	1	1	1	1	1	1	1
247	Consultant Endocrinologist	GTM	More than 10 years	20-29 patients	1	2	1	2	1	1	1	1	2	1	1	1	1	1	1	1	1	1	2	2	2	1	1	1	2	1	1	1	1	1	1	1	1	2	1	1	1	1	1	1	1	1
248	Consultant Cardiologist	AUS	6 - 10 years	20-29 patients	1	2	2	2	2	2	1	2	2	2	2	2	2	1	2	1	1	2	2	1	2	2	1	2	1	2	1	2	2	1	1	2	2	1	2	2	1	2	2	2	1	2
249	Consultant Endocrinologist	KOR	More than 10 years	60-69 patients	1	1	2	1	1	1	2	1	2	1	1	1	1	1	2	1	1	1	1	1	1	2	2	2	2	1	2	3	2	1	1	2	2	2	1	2	2	2	1	2	2	1
250	Consultant Cardiologist	AUS	More than 10 years	100+ patients	2	2	2	2	2	1	2	1	1	2	2	2	1	2	1	2	2	1	1	1	2	1	2	1	2	1	2	2	2	1	2	1	1	2	2	1	1	2	2	2	1	2
251	Primary Care Physician	EGY	More than 10 years	40-49 patients	1	1	2	2	2	1	2	2	2	2	2	1	1	2	2	1	2	2	2	2	1	2	2	1	1	2	1	1	2	1	2	2	2	2	2	2	1	2	2	1	2	2
252	Consultant Endocrinologist	EGY	More than 10 years	30-39 patients	1	1	2	1	1	1	2	2	1	1	2	1	2	3	2	1	1	2	1	2	2	2	1	2	1	1	1	1	1	1	1	1	2	1	2	1	1	2	1	1	1	1
253	Consultant Endocrinologist	EGY	More than 10 years	50-59 patients	1	1	2	1	1	1	1	1	1	1	1	1	1	2	1	1	1	1	1	1	1	1	1	1	1	1	1	1	1	1	1	1	1	1	1	1	1	1	1	1	1	1
254	Primary Care Physician	EGY	6 - 10 years	80-89 patients	2	1	1	1	1	1	1	2	2	1	2	2	2	2	2	1	1	2	2	2	3	2	2	2	1	1	2	1	1	1	1	1	1	1	1	2	2	1	1	2	2	1
255	Primary Care Physician	EGY	More than 10 years	90-99 patients	1	1	1	1	1	1	2	1	2	2	2	2	1	1	1	2	2	1	2	2	1	1	2	2	2	1	1	1	2	1	2	2	2	1	1	2	2	1	1	1	2	1
256	Primary Care Physician	EGY	Less than 5 Years	70-79 patients	2	1	2	1	1	2	1	1	1	2	1	2	1	1	2	1	2	1	2	2	2	1	2	2	1	1	1	2	1	1	2	2	1	2	1	1	2	1	2	1	1	2
257	Primary Care Physician	EGY	6 - 10 years	80-89 patients	2	1	2	2	1	1	2	2	1	1	2	2	1	2	2	2	1	1	1	2	1	2	1	1	1	2	2	2	1	1	1	2	2	1	1	2	1	2	1	2	1	1
258	Consultant Endocrinologist	EGY	6 - 10 years	100+ patients	3	1	1	1	1	1	1	2	1	1	2	1	1	2	1	1	2	2	2	2	3	2	1	2	2	3	1	2	2	3	3	2	2	1	1	1	1	2	1	1	1	1
259	Consultant Nephrologist	EGY	More than 10 years	90-99 patients	1	2	2	2	2	1	2	2	3	2	2	2	2	2	1	2	2	2	3	3	2	3	3	2	2	2	1	3	2	2	2	2	2	2	3	2	2	2	2	2	2	2
260	Consultant Nephrologist	EGY	6 - 10 years	80-89 patients	1	2	2	1	1	1	1	2	1	1	1	1	1	1	1	1	1	2	2	2	2	1	2	2	3	1	1	1	1	1	1	1	1	1	1	1	2	2	2	2	2	2
261	Primary Care Physician	EGY	Less than 5 Years	70-79 patients	1	2	2	2	2	1	2	2	3	2	2	2	2	2	1	2	2	2	3	3	2	3	3	2	2	2	1	3	2	2	2	2	2	2	3	2	2	2	2	2	2	2
262	Consultant Endocrinologist	EGY	More than 10 years	80-89 patients	2	2	1	2	2	1	1	2	1	1	2	1	1	2	2	1	2	2	3	3	2	3	3	2	2	2	1	3	2	2	2	2	2	2	3	1	2	2	1	2	2	2
263	Consultant Endocrinologist	MEX	More than 10 years	10-19 patients	1	3	1	1	1	1	1	1	2	1	1	2	2	2	1	1	1	1	1	1	1	3	3	3	3	2	1	3	1	3	3	1	2	3	1	1	2	1	2	1	1	2
264	Consultant Nephrologist	BRA	6 - 10 years	60-69 patients	1	1	2	1	1	1	2	1	2	1	2	1	2	2	1	1	2	2	2	2	3	2	2	2	2	2	1	1	1	1	2	2	1	2	1	1	1	2	2	1	1	1
265	Consultant Nephrologist	BRA	6 - 10 years	60-69 patients	1	2	1	1	1	1	1	2	2	1	1	2	1	2	1	1	1	1	2	1	1	1	1	2	2	2	1	1	1	1	1	1	1	1	1	1	2	2	1	1	1	1
266	Consultant Nephrologist	EGY	More than 10 years	100+ patients	1	2	1	2	2	1	1	2	3	2	2	2	2	2	1	2	2	1	3	3	2	3	3	2	2	2	1	3	2	2	2	2	2	2	3	1	1	2	2	2	1	2
267	Primary Care Physician	EGY	Less than 5 Years	70-79 patients	1	1	2	1	1	1	1	1	2	2	1	1	2	2	1	1	1	2	2	2	1	2	1	2	2	2	1	2	2	1	1	1	1	1	1	1	1	1	1	1	1	1
268	Primary Care Physician	EGY	6 - 10 years	80-89 patients	1	1	1	1	2	1	1	1	1	1	2	1	1	2	2	1	1	1	1	2	1	2	1	3	2	2	1	1	1	1	1	1	1	1	1	2	1	1	1	1	1	1
269	Primary Care Physician	EGY	Less than 5 Years	70-79 patients	1	1	1	1	2	3	2	1	1	1	2	2	3	2	2	1	1	1	1	1	2	1	1	2	1	1	1	2	1	1	1	1	1	1	1	1	1	1	1	1	1	1
270	Primary Care Physician	EGY	Less than 5 Years	80-89 patients	1	1	1	1	1	1	2	1	1	1	2	1	2	1	1	1	1	2	2	1	2	2	2	1	2	1	1	1	1	1	1	1	1	1	1	2	1	1	1	1	1	1
271	Primary Care Physician	EGY	6 - 10 years	70-79 patients	1	1	1	1	1	1	2	1	1	2	1	2	2	1	1	1	1	2	1	1	2	2	1	2	2	1	1	1	1	1	1	1	1	1	1	1	1	1	1	1	1	1
272	Consultant Endocrinologist	EGY	More than 10 years	70-79 patients	1	1	1	1	1	1	2	2	1	1	1	1	1	1	1	1	1	2	2	2	2	1	2	2	3	1	1	1	1	1	1	1	1	1	1	1	2	2	2	2	2	2
273	Consultant Cardiologist	EGY	6 - 10 years	40-49 patients	1	1	1	1	1	2	2	2	1	1	2	1	1	1	1	1	1	2	2	2	2	2	2	1	2	2	1	1	1	1	1	1	1	1	1	1	1	1	1	1	1	1
274	Consultant Cardiologist	EGY	6 - 10 years	50-59 patients	1	1	1	1	1	1	2	2	1	1	2	2	1	1	1	1	1	2	2	2	2	2	1	1	2	2	1	1	1	1	1	1	1	1	1	1	1	1	1	1	1	1

Key

1 - Strongly Agree, 2 - Tend to Agree, 3 - Tend to Disagree, 4 - Strongly Disagree

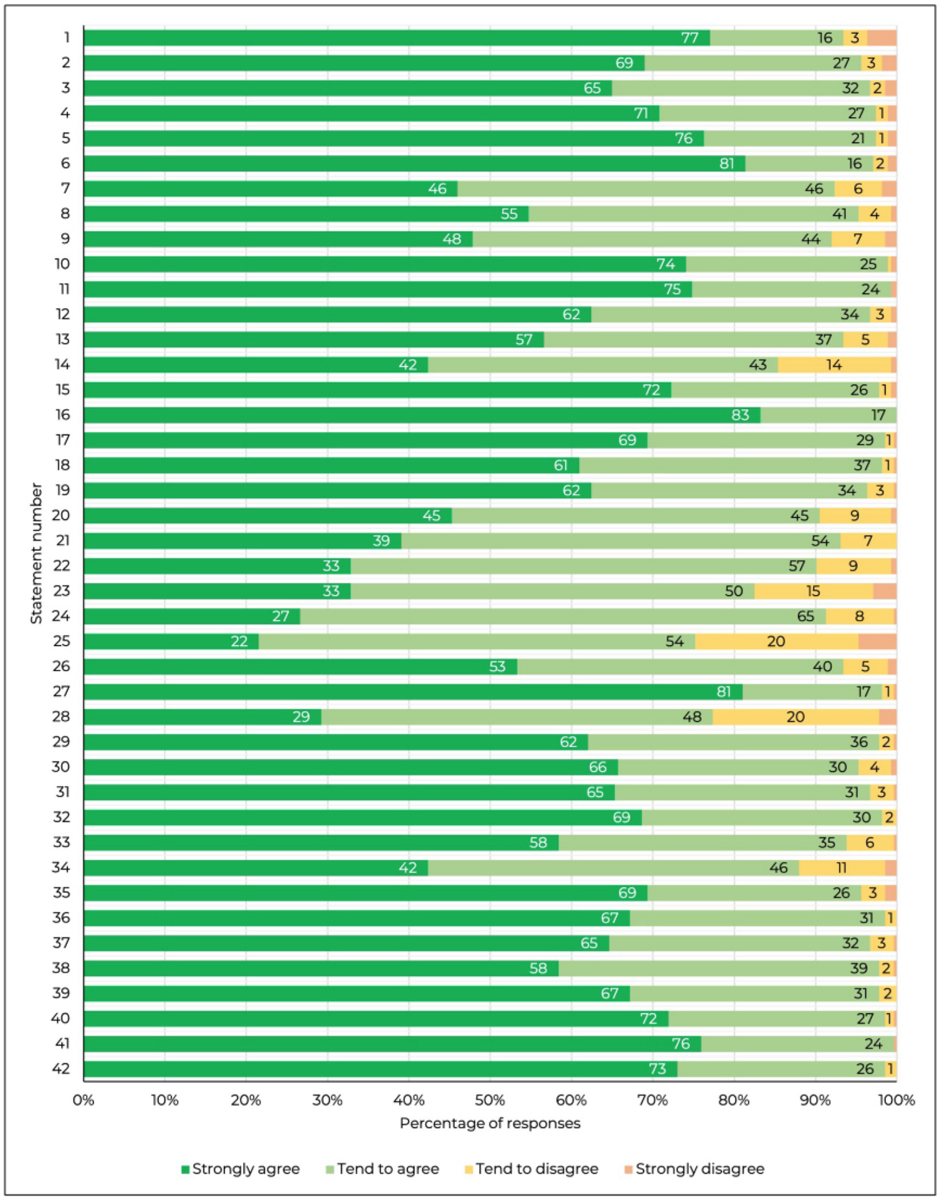

306x390mm (72 x 72 DPI)

CREDES Checklist:

Recommendations for the Conducting and REporting of DElphi Studies (CREDES)[1]

Results from a cross-specialty consensus on optimal management of the chronic kidney disease (CKD) patient: from screening to complications

Items of reporting	Reported on page
Purpose and rationale. The purpose of the study should be clearly defined and demonstrate the appropriateness of the use of the Delphi technique as a method to achieve the research aim. A rationale for the choice of the Delphi technique as the most suitable method needs to be provided.	5 - 6
Expert panel. Criteria for the selection of experts and transparent information on recruitment of the expert panel, sociodemographic details including information on expertise regarding the topic in question, (non)response and response rates over the ongoing iterations should be reported.	5 - 6
Description of the methods. The methods employed need to be comprehensible; this includes information on preparatory steps (How was available evidence on the topic in question synthesised?), piloting of material and survey instruments, design of the survey instrument(s), the number and design of survey rounds, methods of data analysis, processing and synthesis of experts' responses to inform the subsequent survey round and methodological decisions taken by the research team throughout the process.	5 - 7
Procedure. Flow chart to illustrate the stages of the Delphi process, including a preparatory phase, the actual 'Delphi rounds', interim steps of data processing and analysis, and concluding steps.	Figure 1
Definition and attainment of consensus. It needs to be comprehensible to the reader how consensus was achieved throughout the process, including strategies to deal with non-consensus.	7
Results. Reporting of results for each round separately is highly advisable in order to make the evolving of consensus over the rounds transparent. This includes figures showing the average group response, changes between rounds, as well as any modifications of the survey instrument such as deletion, addition or modification of survey items based on previous rounds.	8 - 9
Discussion of limitations. Reporting should include a critical reflection of potential limitations and their impact of the resulting guidance.	14 - 15
Adequacy of conclusions. The conclusions should adequately reflect the outcomes of the Delphi study with a view to the scope and applicability of the resulting practice guidance.	10 - 16
Publication and dissemination. The resulting guidance should be clearly identifiable from the publication, including recommendations for transfer into practice and implementation.	15 - 16

1. Jünger S, Payne SA, Brine J, Radbruch L, Brearley SG. Guidance on Conducting and REporting DElphi Studies (CREDES) in palliative care: Recommendations based on a methodological systematic review. Palliat Med. 2017;31: 684–706. doi:10.1177/0269216317690685

For peer review only
